# A taxonomic revision of the genus *Selaginella* (Selaginellaceae) from Nepal

**DOI:** 10.3897/phytokeys.133.37773

**Published:** 2019-10-07

**Authors:** Aleksandr Petrovich Shalimov, Yu-Dong Wu, Xian-Chun Zhang

**Affiliations:** 1 State Key Laboratory of Systematic and Evolutionary Botany, Institute of Botany, Chinese Academy of Sciences (CAS), Beijing 100093, China Institute of Botany, Chinese Academy of Sciences Beijing China; 2 University of Chinese Academy of Science, Beijing, 100049, China University of Chinese Academy of Science Beijing China

**Keywords:** Lycophytes, Nepalese flora, new records, taxonomy

## Abstract

The present paper deals with the taxonomy of *Selaginella* from Nepal based on the examination of herbarium collections housed in major herbaria of Europe and Asia (with additional collections from virtual herbaria). A total of 25 species are recognised here, while *Selaginella trichophylla* and *S. laxistrobila* are two new records for the flora of Nepal, India (Sikkim) and Bhutan; Selaginella monospora var. ciliolata is synonymised to *S. trichophylla*; detailed descriptions, distribution and ecology and IUCN conservation status assessments (based on literature) are presented. For most of the species, illustrations of the leaves and strobili are provided for identification of the morphologically similar taxa.

## Introduction

*Selaginella* P. Beauv. is the largest genus of lycophytes, with more than 700 species distributed all over the world but with highest diversity in the tropics (Jermy 1986, Zhou et al. 2015, PPG I 2016, Weststrand and Korall 2016). The Himalaya is one of the diversity centers of ferns and lycophytes of the World. Alston (1945) published the first account of Indian (including Nepalese) *Selaginella* P. Beauv. Earlier Don (1824), in his “Prodromus Florae Nepalensis”, described seven species in *Lycopodium* L., including species of heterosporus *Selaginella*. Thapa (2002) listed 23 species of *Selaginella*. Fraser-Jenkins et al.’s (2015) revision of Nepalese *Selaginella* also recognised 23 species.

The purpose of our study was to revise the taxonomy of the genus *Selaginella* of Nepal, providing a taxonomic treatment with diagnostic keys by macro-morphological characters.

## Material and methods

This study was based on materials deposited at the following herbaria: AAU, B, BM, E, GH, K, KATH, KUN, KYO, L, P, PE, TI and US (herbarium acronyms follow Thiers 2019). Images of type specimens of all species from Nepal and neighboring countries were studied by accessing those at E (http://data.rbge.org.uk/herb), K (http://apps.kew.org/herbcat/gotoHomePage.do), P (https://science.mnhn.fr/all/search), B (http://ww2.bgbm.org/herbarium/default.cfm), GH (https://huh.harvard.edu/collections/gray.html), US (http://collections.si.edu/search/results.htm), L (https://bioportal.naturalis.nl) and JSTOR Global Plants project database (https://plants.jstor.org). It is worth noting that part of the collections from Edinburgh Botanic Garden (E) and British Natural History Museum (BM) were borrowed and carefully examined in the Herbarium PE (Beijing). About 350 herbarium specimens, including types for most species associated with taxonomy of *Selaginella* from Nepal, and many photos of the herbarium collections from KYO and TI provided by Mr. C.R. Fraser-Jenkins, were checked.

Morphological characters, such as ventral (lateral), dorsal (median) and axillary leaves were carefully observed. The morpho-photographs of the plants were taken with a Nikon DXM 1200F camera connected to a stereomicroscope (Nikon SMZ 1000) and computer and measurement was done by D 3.10 (http://www.nikoninstruments.com).

Descriptions of the species follow the form of Zhang et al. (2013), with minor changes, and were prepared based on examined dried herbarium specimens from Nepal and neighboring countries. IUCN categories (IUCN 2001) are based on published data/assessments following Fraser-Jenkins et al. (2015).

The distribution information was gathered from herbarium specimens, and literature.

## Taxonomic treatment

### Key to species of *Selaginella* from Nepal

**Table:** 

1	Sporophylls monomorphic	**2**
–	Sporophylls dimorphic	**11**
2	Leaves dimorphic or slightly dimorphic	**3**
–	Leaves monomorphic, spirally arranged on all sides of stem and branches, linear-lanceolate	**1. *S. indica***
3	Rhizophores restricted to base of stem, forming thick massive rootstock	**4**
–	Rhizophores at intervals throughout creeping stem and branches or in basal part	**5**
4	Main stems branched near and above base, rosette plants, xerophytic	**2. *S. pulvinata***
–	Main stems branched from near middle part, not rosette plants, xerophytic	**3. *S. bryopteris***
5	Stems and branches cylindrical, often reddish, sterile leaves not obviously dimorphic, or almost monomorphic, adpressed to stems and branches	**6**
–	Stems and branches cylindrical, not reddish, sterile leaves dimorphic	**7**
6	Leaves ciliolate at margin	**4. *S. adunca***
–	Leaves entire or slightly denticulate at margin	**5. *S. aitchisonii***
7	Plants with creeping subterranean rhizome and stolons	**8**
–	Plants with rhizophores at intervals throughout length of main stem, borne on ventral or dorsal side in axils of branches	**9**
8	Plants 50–100 cm long, main stem erect, leaves ciliate at base	**6. *S. fulcrata***
–	Plants up to 16–65 cm, leaves denticulate with false vein on each side of midvein	**7. *S. involvens***
9	Rhizophores borne on ventral side in axils of branches, ventral and dorsal leaves ovate, margin dentate-serrulate	**8. *S. pallida***
–	Rhizophores borne on dorsal side in axils of branches	**10**
10	Stem articulate, ventral leaves ovate-lanceolate	**9. *S. remotifolia***
–	Stem not articulate, ventral leaves oblong-lanceolate	**10. *S. semicordata***
11	Strobili cylindrical or rather lax	**12**
–	Strobili dorsiventrally complanate	**14**
12	Strobili cylindrical, sporophylls monomorphic	**11. *S. helvetica***
–	Strobili not cylindrical, sporophylls rather lax, often forked	**13**
13	Plants to 25 cm long, ventral leaves ovate, ovate-triangular or ovate-lanceolate, margin denticulate	**12. *S. pallidissima***
–	Plants to 6 cm high, ventral leaves ovate-triangular, margin ciliolate	**13. *S. laxistrobila***
14	Apex of dorsal leaves mucronate or aristate, arista curved	**15**
–	Apex of dorsal leaves acuminate or aristate	**16**
15	Apex of dorsal leaf aristate, arista curved, up to 1/2–4/5 as long as leaf, margin sparsely ciliolate; ventral leaves oblong, apex apiculate, margin ciliolate or denticulate	**14. *S. bisulcata***
–	Apex of dorsal leaf arista, up to 1/2–3/4 as long as leaf; ventral leaves oblong or oblong-ovate, apex acute or apiculate, margin sparsely shortly ciliolate	**15. *S. pennata***
16	Main stems tuberous at base	**16. *S. chrysocaulos***
–	Main stems not tuberous at base	**17**
17	Sporophylls at margin long ciliate	**17. *S. ciliaris***
–	Sporophylls at margin dentate, or not long ciliate	**18**
18	Plants creeping	**19**
–	Plants sub-erect or creeping	**20**
19	Plants long creeping, ventral leaves ovate-triangular or oblong-falcate, margin denticulate; dorsal leaves ovate-lanceolate or elliptic, margin denticulate, apex acuminate or shortly aristate	**18. *S. monospora***
–	Plants long creeping, ventral leaves ovate-triangular, margin ciliolate, dorsal leaves ovate, margin ciliolate, apex aristate	**19. *S. trichophylla***
20	Plants creeping	**21**
–	Plants sub-erect or ascending	**22**
21	Plants ascending from decumbent base, leaves on main stems rather approximate, base of ventral leaves long ciliolate; axillary leaves ovate or ovate-lanceolate, margin shortly ciliolate	**20. *S. repanda***
–	Plants up to 10 cm, creeping, fertile stems erect, leaves on main stems and branches distant, margin denticulate in basal half, elsewhere subentire, or very ciliolate at base; axillary leaves ovate-triangular, margin ciliolate in basal half, elsewhere subentire	**21. *S. vaginata***
22	Plants c. 15 cm long, acroscopic base of ventral leaves dentate	**23**
–	Plants more 15 cm long, acroscopic base of ventral leaves dentate or dentate-ciliolate	**24**
23	Apex of dorsal leaves shortly cuspidate	**22. *S. chrysorrhizos***
–	Apes of dorsal leaves not cuspidate	**23. *S. reticulata***
24	Ventral leaves ovate to ovate-lanceolate, acroscopic base ciliate-dentate, auriculate at base; dorsal leaves ovate, base obtuse or slightly subcordate, margin ciliolate to denticulate	**24. *S. subdiaphana***
–	Ventral leaves ovate, acroscopic base denticulate, base cordate, dorsal leaves ovate, base subcordate, margin minutely dentate	**25. *S. tenuifolia***

### Selaginella indica

Taxon classificationPlantaeSelaginellalesSelaginellaceae

(Milde) R.M. Tryon

2FDF6425-9C50-548D-A379-44880A3AB096

**Figure 1.**
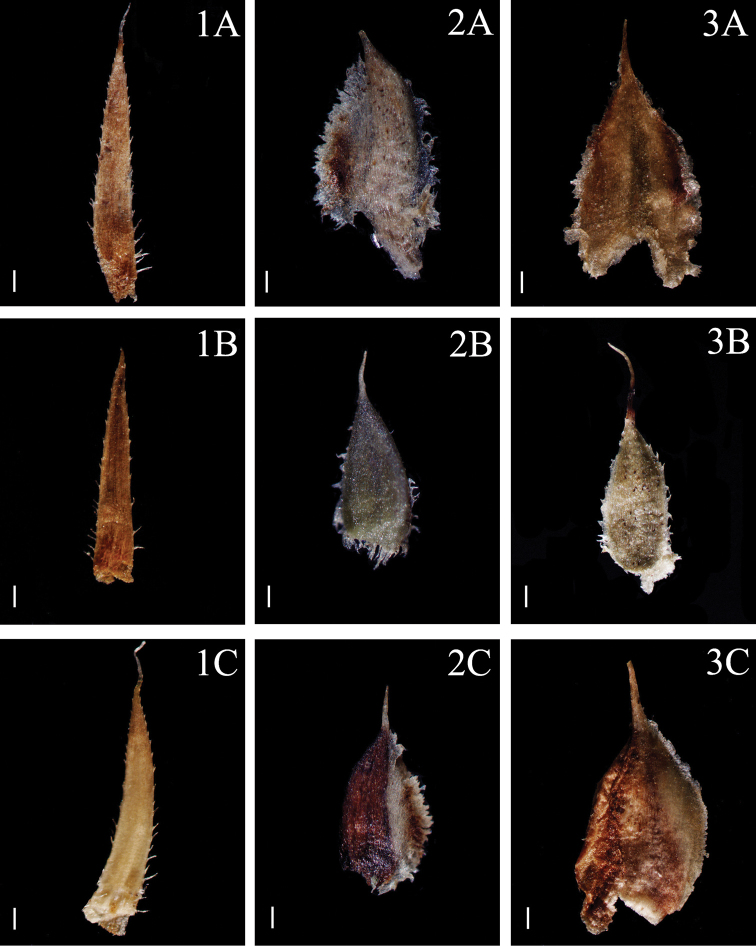
Morphological diversity of the leaves of Nepalese *Sealginella* species: **1A–C***S. indica* (*Nakaike 1325*, PE) **2A–C***S. pulvinata* (*Tabata* et al. *3520*, PE) **3A–C***S. bryopteris* (*Tabata* et al. *11989*, PE). A – Axillary leaves, B – Dorsal leaves, C – Ventral leaves. Scale bars: 0.2 mm.

Figs 1 (1A–C), 9A , 12

Selaginella indica R.M. Tryon, Ann. Missouri Bot. Gard. 42: 52, f. 23, map 32. 1955; Dixit 1992; Zhang 2001; Thapa 2002; Zhang 2004; Zhang et al. 2013; Fraser-Jenkins et al. 2018.
(Selaginella rupestris f. indica Milde, Fil. Eur. Atlan.: 262. 1867, nom. nud.).
≡ Bryodesma indicum (R.M. Tryon) Soják, Preslia 64(2): 154. 1992. **Type.** INDIA. Khasia 5000 ft., *Hooker fil. & Thomson* (holotype: GH [00022087]; isotypes: NY [00127400]; P [00279924]; YU [000626]).
= Selaginella emodi Fraser-Jenk., Ferns Fern-Allies Nepal 1: 67. 2015. **Type.** NEPAL. Central Nepal, Rasuwa District, on path leading up from Dhunche to Chandanbari and Gossainkund, c. 3 km above and E. of Dhunche, N. of Trisuli Bazaar, Rasuwa District, N. of Kathmandu, rocky pathside tussocks of grasses etc. beneath cliff, 2 XII 2004. *C. R. Fraser-Jenkins 30915* (holotype: TAIF).
– Selaginella wightii auct. non Hieron.: Panigrahi and Dixit 1968.
– Selaginella vardei auct. non H. Levl.: Ching & Wu, Fl. Xizang. 1: 19, p.p. excl. figure. 1983; H.S. Kung 1988; K.H. Shing 1993.
– Selaginella longipila auct. non Hieron; Alston 1945; Tryon 1955; Dixit 1992.

#### Description.

Stems 5–15 cm, creeping. Rhizophores at intervals throughout the length of the creeping stem and branches, borne on dorsal side in axils of branches, densely hairy. Main stems anisotomously branched throughout, strongly dorsi-ventral in position, glabrous. Lateral branches arranged on main stem 0.5–1 cm apart, second branches simple or forked. Leaves spirally arranged on all sides stem and branches, more or less isomorphic, long linear-lanceolate, 0.8–2.3 mm excluding seta, 0.3–0.5 mm wide, margin shortly ciliolate, apex acuminate, in apex with long apical seta c. 1/5 as long as leaves. Strobili solitary on erect branchlets, tetragonal, 5–25 × 1.5–2 mm. Sporophylls monomorphic, ovate-triangular or ovate-lanceolate, margin ciliolate, apex acuminate. Megaspores pale-orange, surface rugose; microspores deep yellow, surface rugose to reticulate.

#### Ecology.

Epilithic, xerophytic, summer-green, in dry areas, forming clumps on moss covered rocks. Alt. 1350–2800 m.

#### Distribution in Nepal.

W, C, E.

Nepalese threatened status: NT (Fraser-Jenkins et al. 2015).

#### General distribution.

CHINA (Sichuan, Xizang, Yunnan), INDIA (Andhra Pradesh, Chhattisgarh, Jharkhand, Karnataka, Madhya Pradesh, Meghalaya, Odisha, Tamil Nadu, Uttarakhand, West Bengal).

#### Chromosome number.

not available data.

Selected specimens examined:

**W Nepal: DARCHULA**: “Nakarigad-Khandeswori, on mossy slope in open pine forest, alt. 1650 m, 18 Jul 1984, *P.R. Shakya*, *M.K. Adhikari*, *M.N. Subedi 7882*” (KATH).

**C Nepal: RASUWA**: “between Lama Hotel and Sharpugaon, alt. 2600–2800 m, 3 Sep 1986, *T. Nakaike 1325*” (PE).

**E Nepal: TAPLEJUNG**: “Takhtem to Chautara, 1350 m, 12 May 1992, *N. Acharya 9255056*”, (KATH).

### Selaginella pulvinata

Taxon classificationPlantaeSelaginellalesSelaginellaceae

(Hook. & Grev.) Maxim.

99EF9ADD-3939-5278-A429-FF3862668D09

Figs 1 (2A–C), 13

Selaginella pulvinata (Hook. & Grev.)Maxim., Mém. Acad. Imp. Sci. Saint Pétersb. (Sér. 7) 9: 335. 1859; Dixit 1992; Thapa 2002; Zhang 2004; Zhang et al. 2013; Fraser-Jenkins et al. 2015; Fraser-Jenkins et al. 2017.
≡ Lycopodium pulvinatum Hook. & Grev., Hooker’s J. Bot. Kew Gard. Misc. 2: 381. 1831.
≡ Selaginella tamariscina var. pulvinata (Hook. & Grev.) Alston, Bull. Fan Mem. Inst. Biol., Bot. 5(6): 271. 1934.
(Selaginella pulvinata (Hook. & Grev.) Hand.-Mazz., Symb. Sin. 6: 5. 1929, later isonym).
≡ Lycopodioides pulvinata (Hook. & Grev.) H.S. Kung, Fl. Sichuanica 6: 64, t. 18, f. 1–3. 1988. **Type.** INDIA. E. India, Kamoon. *Dr. Wallich* s.n. (holotype: K).

#### Description.

Stems 2–15(–20) cm, many forming rosette at top of thick rootstock, branched from base. Main stems branched near and above base, primary branches pinnately branched, second branches 2–3 forked, stramineous or brown, main stem c. 1 mm in diam. at lower part. Axillary leaves ovate to triangular, c. 2.5 × 1 mm, base exauriculate, margin lacerate-ciliolate, apex acute. Ventral leaves ovate, 2.9–3.2 × 1.4–1.5 mm, rotundate-cordate at base, margin lacerate, apex acuminate. Dorsal leaves ovate, 2.8–3.1 × 0.9–1.2 mm, base truncate, entire to obscurely denticulate, posterior side thickened, apex aristate. Strobili solitary, terminal, compact, tetragonal, 7–15(–20) × 1.5–2 mm. Sporophylls monomorphic, ovate, at base cordate, margin slight denticulate, apex acuminate. Megaspores white-yellow, surface verrucate; microspores yellow, surface irregularly papillate.

#### Ecology.

Terrestrial or epilithic, xerophytic. Alt. 1800–4400 m.

#### Distribution in Nepal.

W.

Nepalese threatened status: EN (Fraser-Jenkins et al. 2015).

#### General distribution.

CHINA (Chongqing, S Gansu, Guangxi, Guizhou, Hebei, Henan, Liaoning, Shaanxi, Shanxi, Sichuan, Xizang), INDIA (Uttarakhand), KOREA, MONGOLIA, RUSSIA (Siberia), THAILAND, VIETNAM.

#### Chromosome number.

not available data.

Selected specimens examined:

**W Nepal: BAJHANG**: “Bauligad, on open rock, rooting on crevices, alt. 1830 m, 6 Jul 1980, *P.R. Shakya*, *L.R. Sharma*, *K.R. Amatya 6328*” (KATH).

**DOLPA**: “between Besagad and Shahartara, 14 Sep 1976, *H. Tabata*, *K.R. Rajbhandari*, *Y. Shimizu 3520*” (PE).

### Selaginella bryopteris

Taxon classificationPlantaeSelaginellalesSelaginellaceae

(L.) Baker

37BFCFB2-6370-5F20-9912-66BBB8173AD0

Figs 1 (3A–C), 9B , 14

Selaginella bryopteris (L.) Baker, J. Bot. 22(Za): 376. 1884; Iwatsuki 1988; Dixit 1992; Thapa 2002; Fraser-Jenkins et al. 2015; Fraser-Jenkins et al. 2017.
≡ Lycopodium bryopteris L., Sp. Pl. 2: 1103. 1753.
≡ Lycopodioides bryopteris (L.) Kuntze, Revis. Gen. Pl. 1: 825. 1891. **Type.** (lectotype, designated by Mazumdar 2017). Dillenius (1741), Historia Muscorum. P. 472, t. 66, f. 11 [icon.] (fig. 1). **Epitype.** (designated by Mazumdar 2017): INDIA. Jharkhand: Deoghar, Trikut Hills, 365.76 m. 11 X 1999. *J. Mazumdar 72* (CAL).
= Lycopodium circinale L., in Murray, Syst. Veg. ed 13: 794. 1774.
= Lycopodium imbricatum Roxb., in Griff., Calc. J. Nat. Hist. 4: 475. 1844; non Forssk. 1775; Thapa 2002.
≡ Selaginella imbricata (Roxb.) J. Scott, J. Agr. Hort. Soc. India, n. s. 2: 260. 1868.
(Lycopodium imbricatum Roxb., Hort. Bengal.: 75. 1814, nom. nud.).

#### Description.

Stems 5–25 cm, suberect to erect. Rhizophores at lower and basal part stem. Main stems branched from near middle part, in basal part main stem 1.5–2.5 mm in diam. Axillary leaves slightly similar with ventral leaves, base cuneate, margin irregular, finely denticulate, 2.0–3.5 × 1.3–2.0 mm. Ventral leaves ovate, 1.5–2.0 × 1.0–1.5 mm, oblique at base, imbricate, margin denticulate, acute to acuminate at apex. Dorsal leaves ovate, 1.4–1.8 × 1.2–1.5 mm, slightly asymmetric, oblique at base, margin entire to minutely denticulate, apex acute to acuminate. Strobili rare, solitary, terminal, compact, 3–5 × 1–2.5 mm. Sporophylls monomorphic, ovate, margin entire to minutely denticulate, apex acuminate. Megaspores dull-yellow, surface verrucate, microspore yellow, surface granulate.

#### Ecology.

On rocks in dense forests at lower elevation. Alt. 250–1700 m.

#### Distribution in Nepal.

W, C, E.

Nepalese threatened status: not available data.

#### General distribution.

BHUTAN, INDIA (Assam, Darjeeling, Uttarakhand, NE, C and S India), ARABIA, N AFRICA.

#### Chromosome number.

2n=20 (Fabbri 1965; Jermy et al. 1967).

Selected specimens examined:

#### Nepal.

“De la Banaoüra Khola à Balauta, alt. 400 m. 6 XI 1954. *A. Zimmernann* 2077” (KYO, photo).

**W Nepal: HUMLA**: “Between Danna and Sali Salla (the junction of Loti Gad and Humla Karnali River), dry rock on SW-facing slope, alt. 1740 m, 26 Sep 1983, *H. Tabata* et al. *23629*” (KYO, photo).

**HUMLA, MUGU**: “Between Tirthasain, Humla Dist., and Huanglu, Mugu Distr., on the trail in grassland on W-facing slope, alt. 1400 m, 5 Sep 1983, *H. Tabata* et al. *24648*” (KYO, photo).

**SALYAN**: “Salyana, alt. 5000 ft., dry earth bank beside track, 29 Mar 1952, *O. Polunin*, *W.R. Sykes* & *L.H.J. Williams 667*” (US, photo; KYO, photo).

**C Nepal: CHITAWAN**: “damp, rocky sides of small steam-gully at Lambola Khola, c. ½ km S of Beldas (Satara Kilo) village, 18 km S. of Mugling on road to Narayanghat, S. W. of Kathmandu, alt. 350 m, 20 Jan 2000, *C.R. Fraser-Jenkins 38395 (FN 4370)*” (US, photo).

**E Nepal: TEHRATHUM**: “en route from Iwa to Majhi, on dry slope along the river, Shorea-Shima forest, 610 m, 29 Jun 1978, *H. Tabata*, *K.R. Rajbhandari*, *Y. Shimizu 11989*” (PE).

**DHANKUTA**: “Dhankuta, 26°50'N, 87°20'E, alt. 400 m, 11 Oct 1971, *J.F. Dobremez* DBR NEP *1373*” (E00670572); “by River Tamur, near Suspension Bridge, alt. 1000 ft, 18 Sep 1961, *A.H. Norkett 5094*” (BM001022383).

**TAPLEJUNG**: “Tamur Bridge, alt. 250–300 m. 4 Sep 1977, *H. Ohashi* et al. *773141*” (TI, photo).

### Selaginella adunca

Taxon classificationPlantaeSelaginellalesSelaginellaceae

A. Braun ex Hieron.

C6A1312D-ABB5-5029-927E-8F7993E7A2BF

Figs 2 (1A–C), 15

Selaginella adunca A. Braun ex Hieron., in Engl. and Prantl, Nat. Pflanzenfam. 1(4): 674. 1901; Dixit 1992; Fraser-Jenkins et al. 2015; Fraser-Jenkins et al. 2017. **Type.** INDIA. *Falconer 34* et *1230* p.p. (syntypes: B).

#### Description.

Stems 10–25 cm, erect. Rhizophores restricted in basal part of stems, located on ventral side. Main stems branched from middle upward, decumbent, simple in basal region, branches decompound, close together, flabellate, in basal part main stem 1.2–1.5 mm in diam. Main stems terete, bright and sometimes stramineous-red. Axillary leaves symmetrical, oblong, base exauriculate, margin ciliolate. Ventral leaves asymmetrical, oblong, 0.9–1.4 × 0.6–1 mm, subfalcate, acroscopic base dilated, ciliate at base, rest dentate to denticulate above, apex cuspidate. Dorsal leaves small, elliptic, 0.7–1.2 × 0.4–0.8 mm, base cuneate truncate, margin dentate, apex cuspidate. Strobili solitary, terminal, compact, tetrahedral, 3.0–5.0 × 1.0–2.2 mm, slightly wider branches. Sporophylls monomorphic, deltoid, cuspidate, margin dentate, strongly keeled. Megaspores reddish-brown, surface verrucate; microspores orange, surface verrucate

#### Ecology.

Terrestrial or epilithic, xerophytic, on open semi-dry stony areas. Alt. 330–2500 m.

#### Distribution in Nepal.

W.

Nepalese threatened status: NT (Fraser-Jenkins et al. 2015). Endemic NW Himalaya, rare.

#### General distribution.

INDIA (Uttarakhand, Himachal Pradesh).

#### Chromosome number.

not available data.

Selected specimens examined:

**W Nepal: KALIKOT**: “Between Kairkot and Lapha, Karnali Valley, crevices of dry cliff., alt. 4500 ft, 26 Apr 1952, *O. Polunin*, *W.R. Sykes & L.H.J. Williams 3984*” (E, photo; US, photo; KYO, photo); “Kiurithanu, Karnali River, growing on vertical rocks, alt. 4000 ft, 21 Apr 1952, *O. Polunin*, *W.R. Sykes & L.H.J. Williams 797*” (E, photo; US, photo; KYO, photo).

**DOLPA**: “Between Phulchangi and Chong, near Tibrikot, growing among stones on dry hot open slopes, alt. 8000 ft, 11 Nov 1952, *O. Polunin*, *W.R. Sykes & L.H.J. Williams 3323*” (AAU; E, photo; US, photo; KYO, photo).

**DANG**: “Between Kurpani and Ghorai, growing on damp sheltered earth banks, alt. 4000 ft, 4 Sep 1952, *O. Polunin*, *W.R. Sykes & L.H.J. Williams 1332*” (E, photo);

**SURKHET**: “Near Kuepani Siwalik Hills, alt. 1000 ft, 27 Oct 1952, *O. Polunin*, *W.R. Sykes & L.H.J. Williams 5920*” (E, photo).

**KALIKOT**: “Taelou, 28°53'N, 82°30'E, 1100 m, 22 Apr 1984, *J.F. Dobremez* DBR NEP *2689*” (E00670605); “Bodi Khola, 1700 m, 25 Apr 1974, *J.F. Dobremez* DBR NEP s.n.” (E00670564).

### Selaginella aitchisonii

Taxon classificationPlantaeSelaginellalesSelaginellaceae

Hieron.

A2E72B26-C125-58B7-B2D4-73386870EC9B

Selaginella aitchisonii Hieron., Nat. Pflanzenfam. 1(4): 674. 1902; Dixit 1992; Thapa 2002; Fraser-Jenkins et al. 2015; Fraser-Jenkins et al. 2017.
≡ Selaginella sanguinolenta f. aitchisonii (Hieron.) Alston, Proc. Nat. Inst. Sci. India 11(3): 215. 1945. **Type.** PAKISTAN. Kurram Valley, Shend Toi, *J.E.T. Aitchison 369* (syntype: B [20 0121871]; K, CAL); KYRGYZSTAN. Turkestan, Akburtaseh, *A. Regel 1878* (syntype: B [20 0121870]); KYRGYZSTAN. Turkestan, Musart-Thal., *A. Regel 1877* (syntype: B [20 0121869]).

#### Description.

Stems 10–25 cm, erect, slender. Rhizophores restricted to the basal part of stems, located on ventral side. Main stems branches from near bases, lateral branches dichotomously compound. Main stems terete, reddish, in basal part main stem 1.0–1.1 mm in diam. Axillary leaves symmetrical, ovate-oblong, carinate, at base obtuse, margin hyaline, at base denticulate, in middle and upper part entire, apex acuminate. Leaves isomorphic, slightly asymmetrical, ovate-lanceolate, uniauriculate at base, obtuse, peltate, margin hyaline, denticulate at base, entire in middle and upper part, apex mucronate to acuminate. Strobili solitary, terminal, compact, tetrahedral, 6.0–10.0 × 1.0–2.0 mm, slightly wider branches. Sporophylls monomorphic, ovate, truncate at base, strongly keeled, apex mucronate to acute. Megaspores yellow, irregular verrucate. Microspores deep yellow, surface rugulose-tuberculate, with perispore on surface.

#### Ecology.

On mossy rocks. Alt. 2200–3400 m.

#### Distribution in Nepal.

W.

Nepalese threatened status: VU, globally threatened (Fraser-Jenkins et al. 2015).

#### General distribution.

AFGHANISTAN, INDIA (Jammu and Kashmir), KYRGYZSTAN, PAKISTAN.

#### Chromosome number.

not available data.

Selected specimens examined:

**W Nepal**: **HUMLA**: “Phal Ko Odar to Pipling, on mossy stone. 2600 m, 6 Jun 1980, *P.R. Shakya & B. Roy 5514*” (KATH).

#### Note.

*Selaginella aitchisonii* is morphologically closely related to the widespread *S. sanguinolenta*, a species complex consists of several morphological variable forms which might be recognised as distinct species pending our molecular phylogentic analysis (data not published).

### Selaginella fulcrata

Taxon classificationPlantaeSelaginellalesSelaginellaceae

(Buch.-Ham. ex D. Don) Spring

DE3E660A-E6D5-52E2-B606-1E8C67DB12FC

**Figure 2.**
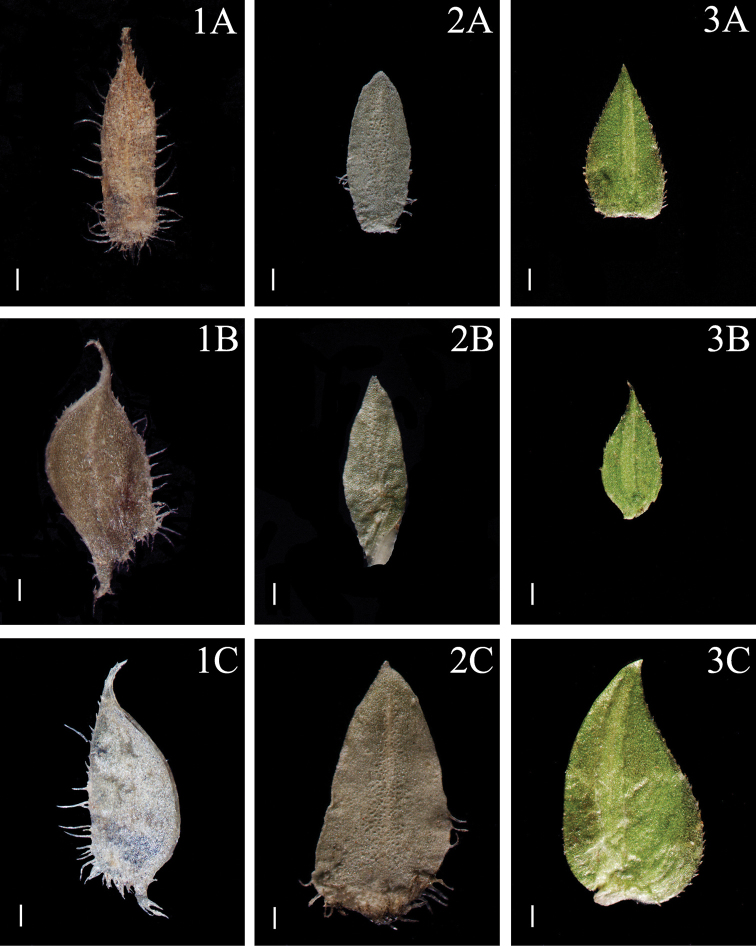
Morphological diversity of the leaves of Nepalese *Sealginella* species **1A–C***S. adunca* (*Strachey* & *Winterbottom 5*, PE) **2A–C***S. fulcrata* (*Nakaike 1923*, PE) **3A–C***S. involvens* (*Zhang 345*, PE). A – Axillary leaves, B – Dorsal leaves, C – Ventral leaves. Scale bars: 0.2 mm.

Figs 2 (2A–C), 9C , 16

Selaginella fulcrata (Buch.-Ham. ex D. Don) Spring, Bull. Acad. Brux. 10: 231, no.138. 1843; Iwatsuki 1988; Dixit 1992; Thapa 2002; Fraser-Jenkins et al. 2015; Fraser-Jenkins et al. 2017.
≡ Lycopodium fulcratum Buch.-Ham. ex D. Don, Prodr. Fl. Nepal. 17. 1824.
≡ Lycopodioides fulcrata (Buch.-Ham. ex D. Don) Kuntze, Revis. Gen. Pl. 1: 826. 1891. **Type.** (lectotype, designated by Fraser-Jenkins et al. 2015) NEPAL. Lycopodium fulcratum Ham. ex D. Don, Prod Fl. Nep. p. 17. Napaul. *Dr. Buchanan* (BM, top of sheet).

#### Description.

Stems glabrous, 50–110 cm, erect. Main stems simple at base, branched from middle part of stem, unequally angular, drying stramineous-brown, 1.3–3.0 mm in diam. in lower part, primary branched copiously pinnate, elongate-deltoid. Axillary leaves ovate-elliptic, 1.2–1.5 × 0.5–0.7 mm, base obtuse, margin ciliate up to middle, rest entire. Ventral leaves ovate-oblong, 1.4–2 × 0.5–0.7 mm, base obtuse, acroscopic base ciliate, rest entire and revolute, basiscopic base with few cilia, apex acute. Dorsal leaves decurrent, 0.8–1.3 × 0.3–0.6 mm, unequally attenuate, subfalcate, subacute, only older leaves ciliate at base, rest entire and younger ones entire throughout. Strobili solitary, terminal, compact, 8–12 × 1–2 mm. Sporophylls monomorphic, ovate, cordate at base, margin denticulate or entie, apex abruptly acute. Megaspores reddish-brown, surface papillate; microspore reddish-brown, surface papillate.

#### Ecology.

On damp sheltered earth banks at lower elevation. Alt. 200–1200 m.

#### Distribution in Nepal.

W, C, E.

Nepalese threatened status: LC (Fraser-Jenkins et al. 2015).

#### General distribution.

INDIA (Bihar).

#### Chromosome number.

not available data.

Selected specimens examined:

“Nepal n. 1397, Herb. Geheed, Oktbr. 1909” (B); “Nepalia, *Wallich* s.n.”(K); “Mountain Nepalia, *E.J.C*. n. *125*” (E, photo); “Nepalia s.n.” (E00670612, photo); “Nepalia, 1823, *Wallich 125*” (K, photo); “Nepalia, 1829, *Wallich 125*” (K, photo); “Belsot a Sogaret, 130 m, 9 Nov 1954, *A. Zimmermann 2153*” (KYO, photo).

**W Nepal: DANG**: “Budamar, on moist and chedy place, alt. 310 m. 29 Sep 1982, *N.P. Manandhar 8577*” (KATH); “Kwera Panii, Dang, on shady and rocky places, alt. 600 m, 10 Mar 1976, *N.P. Manandhar*, *P.M. Regmi 204*” (KATH); “Between Kurpani and Ghorai, growing on damp shelered earth banks, alt. 4000 ft, 4 Sep 1952, *O. Polunin*, *W.R. Sykes & L.H.J. Williams 1332*” (US, photo; KYO, photo; E00670606).

**SURKHET**: “Near Kuepani, Siwalik Hills, growing on shady banks, alt. 1000 ft, 27 Oct 1952, *O. Polunin*, *W.R. Sykes & L.H.J. Williams 5920*” (KYO, photo; US).

**C Nepal: GULMI**: “Gundi Khola, Kali Gandaki River, alt. 2500 ft, 13 Oct 1954. *Stainton*, *Sykes*, *L.H.J. Williams 8929*” (E, photo).

**SYANGJA**: “Roadbank at Galyang village, S of Waling, N of Tansen, on road between Pokhara and Butwal. 25 Sep 1997. *C.R. Fraser-Jenkins* et al. *25578* (*FN 1556*)” (US, photo).

**PALPA**: “Argali 833275, on moist slope strobilus green, alt. 800 m, 27 Nov 1973, *D.P. Joshi*, *M.M. Amatya 73/105a*” (KATH).

**CHITAWAN**: “Chitawan, Churia hills, Shorea forest undergrowth, 2000 ft., 14 Jun 1975, *Laurie 77*” (K); “North-East face of Narayani Ghat, alt. 213–360 m, 3 Jan 1977, s.n., *500*” (KATH); “Damp, rocky sides of small stream-gulley at Lambola Khola, c. ½ km S of Belbas (Satara Kilo) village, 18 km S. of Mugling on road to Narayanghat, S. W. of Kathmandu, alt. 350 m, 20 Jan 2000, *C.R. Fraser-Jenkins 28396* (*FN 4371*)” (US, photo).

**MAKAWANPUR**: “Forested ridges and cliffs of the Churiya Ghats, N. of Bagmati Bridge (c. 13 km E. of Chandranigarpur on main road), on path to Bagar, W. side of Baginati River, E. of Hetauda, Makawanpur District, Narayani Zone, E.C. Nepal, 21 Oct 1997. *C.R. Fraser-Jenkins* et al. *25724* (*FN 1702*)” (KATH); “Above Liot village, Basmari, c. 5 km. W of Hetauda, off Narayanghat road. Densely Sal-forested and rocky stream-gully on S. slope of first range of foothills beyond and N of the Churiya Ghats, 24 Oct 1997, *C.R. Fraser-Jenkins* et al. *25760* (*FN 1738*)” (BM001022482).

### Selaginella involvens

Taxon classificationPlantaeSelaginellalesSelaginellaceae

(Sw.) Spring

8018A7CD-C4F7-548F-AE65-021EE923CB31

Figs 2 (3A–C), 9D , 17

Selaginella involvens (Sw.) Spring, Bull. Acad. Roy. Sci. Bruxelles 10(1): 136, no. 6. 1843; Iwatsuki 1975; Iwatsuki 1988; Dixit 1992; Thapa 2002; Zhang 2004; Zhang et al. 2013; Fraser-Jenkins et al. 2015; Fraser-Jenkins et al. 2017.
≡ Lycopodium involvens Sw., Syn. Fil. 182. 1806.
≡ Lycopodioides involvens (Sw.) Kuntze, Revis. Gen. Pl. 1: 826. 1891. **Type.** JAPAN. *Thunberg* in Herb. Swartz (holotype: S; isotype: B [20 0147264]).
= Lycopodium caulescens Wall. ex Hook. & Grev., Bot. Misc. 2: 382. 1831.
≡ Selaginella caulescens (Wall. ex Hook. & Grev.) Spring, Bull. Acad. Roy. Sci. Bruxelles 10(1): 137, no. 12. 1843. **Type.** NEPAL. At the River Rapty, Nepal, *Dr. Wallich 137* (holotype: K [001109362]; isotype BM?).

#### Description.

Plants 15–45(–65) cm, with creeping subterranean rhizome and stolons; leaves on rhizome and stolons scale-like, pale yellow. Rhizophores restricted to basal part. Main stems branched from middle upward, pinnately branched, stramineous, unbranched main stem 3–20 cm tall, 1–1.5 mm in diam. in lower part, terete, not sulcate, glabrous; primary leafy branches 7–12 pairs, 2 or 3 times pinnately branched, secondary branches 1 or 2 times pinnately branched. Axillary leaves ovate to triangular, 1.1–1.6 × 0.4–1.1 mm, base exauriculate, margin denticulate in basal to middle part, to upper entire, apex acute. Ventral leaves ovate to triangular, 1.4–2.4 × 0.4–1.4 mm, basiscopic base rounded, margin entire, acroscopic base enlarged, broader, overlapping stem and branches, margin denticulate, falsely two nerved, apex subacute or apiculate. Dorsal leaves ovate-triangular or ovate-elliptic, 0.6–1.2 × 0.2–0.5 mm, slightly carinate, base cuneate, margin denticulate, apex long acuminate to shortly aristate. Strobili solitary, terminal, compact, tetragonal, 5–15 × 1–1.4 mm. Sporophylls monomorphic, ovate-triangular, margin denticulate, apex acuminate. Megaspores whitish or brown, with equatorial flange, surface with spinulose microsculptures; microspores yellowish orange, surface verrucate with blunt spines.

#### Ecology.

Epilithic or xerophytic, in damp forests or on moss covered boulders and cliffs, evergreen or seasonally green. Alt. 650–3000 m.

#### Distribution in Nepal.

W, C, E.

Nepalese threatened status: not available data.

#### General distribution.

BHUTAN, CHINA (Anhui, Chongqing, Fujian, Gansu, Guangdong, Guangxi, Guizhou, Hainan, Henan, Hubei, Hunan, Jiangxi, Shaanxi, Sichuan, Taiwan, Xizang, Yunnan, Zhejiang), INDIA (N, E, C and S), JAPAN, KOREA, LAOS, MALAYSIA, MYANMAR, PHILIPPINES, SRI LANKA, THAILAND, VIETNAM.

#### Chromosome number.

x=9 (Kuriachan 1963); 2n=18 (Jermy et al. 1967).

Selected specimens examined:

**W Nepal: MUGU**: “Mugu Karnali Valley, between Mangri and Lumsa, growing on wet vertical cliff face in shade, alt. 7000 ft, 26 Aug 1952, *O. Polunin*, *W.R. Sykes & L.H. Williams 3045*” (US, photo; KYO, photo).

**HUMLA**: “Between Ripa and Sunakhada, 29 Aug 1983, *H. Tabata* et al. *23939*” (KYO, photo); “Between Surkegad and Ripa, rock cliff facing east, alt. 1700 m, 27 Aug 1983, *H. Tabata* et al. *23757*” (KYO, photo).

**BAJHANG**: “Bajhang, Agara, 11 Sep 2017, *S.R. Zhang 345*” (PE no. 2525761).

**C Nepal: NUWAKOT, RASUWA**: “Between Betrawati, Nuwakot Dist. and Ramche, Rasuwa Distr., on the rock in the open place, alt. 1450 m, 11 Jun 1983, *H. Tabata* et al. *18029*” (KYO, photo); “Bagmati Zone, beyond Dhunche, in gorge of Trisuli River (coming down from Goissaikund), Northeast facing slopes, on mossy rocks in deep shade, alt. 1700 m, 17 Sep 1966, *D.H. Nicolson 2507*” (US).

**RAMECHAP**: “enroute from Those to Shibalaya, Ramechap District. Along the trail, on the rock, Pinus wallichiana zone, alt. 1750–1780 m, 2 Jun 1978, *H. Tabata*, *K.R. Rajbhandari*, *Y. Shimizu 10211*” (PE 00244240); “Jiri (1860m)–Kune (1860m)–Kattike (2000m)–Those (1740m)–Shivalaya (1800m), alt. 1740–2000 m, 27°37'N, 86°14'E –27°36'N, 86°17'E, 16 Aug 1985, *M. Suzuki*, *N. Kurosaki*, *S.K. Wu 8580885*” (TI, photo).

**DOLAKHA**: “Shemma-Yakuwa-Lamobagar, alt. 1100–1500 m, 1 Aug 1977. *H. Ohashi* et al. *771897*” (TI, photo); “Jire-Those, shade place, alt. 6300 ft, 23 Sep 1964. *Banerjee*, *Shrestha*, *Upadhyay 2884*” (US, photo); “Chumro, alt. 2200 m, 26 Sep 1976, *Y. Suehiro 2326*” (KYO, photo); “Près de Gongar, alt. 1280 m, 14 Sep 1956, *A. Zimmermann 1256*” (KYO, photo).

**KASKI**: “Tamage (1730m)–Banjan (2035m), alt. 2100 m, 28°15'22"–13'03"N, 83°49'56"–48'44"E, 9 Aug 1999, *M. Mikage* et al. *9965056*” (TI, photo); “Pokhara to Hyenda, alt. 1000–1100 m, 20 Sep 1976, *Y. Suehiro 84*” (PE); “Pokhara to Hyenda, alt. 1000–1100 m, 20 Sep 1976, *Y. Suehiro 82*” (TI, photo); “l.c. *Y. Suchiro* 83” (KYO, photo).

**E Nepal: TERHATHUM**: “Basantapur-Chitre, on mossy tree trunk in forest, alt. 2300–2400 m, 7 Jun 1972, *H. Kanai* et al. *725490*” (TI, photo; KYO, photo); “Kunja, 27°13'N, 87°52'E, alt. 2100 m, *J.F. Dobremez* DBR NEP *1315*” (E00754781, E00668259).

**SANKHUWASABHA**: “Seduwa to Kasuwa Khola, prostrate under rock, alt. 3500 ft, 6 May 1965, *Banerjee*, *Upadhyay*, *Baskola 3406*” (US, photo); “Seduwa, in shade under big rock, alt. 5500 ft, 8 Aug 1965, *Banerjee*, *Upadhyay*, *Baskola 3366*” (US, photo); “Senduwa 2100 m – Bhaluhhop 2400 m, 5 Jun 1972, *H. Kanai* et al. *725089*” (KYO, photo).

**TAPLEJUNG**: “Shewaden 2600 m – Mewa Khola 2100 m, on mossy rock in light shade, c. 2200 m, 29 Jun 1972, *H. Kanai* et al. *725353*” (KYO, photo); “Zongi-Iladanda, 12 Nov 1963, *H. Hara* et al.” (TI, photo).

**SOLUKHUMBHU**: “near Namche, in shade andmoist place, alt. 8000 ft, 9 May 1965, *Banerjee* et al. *3415*” (US, photo).

**ILAM**: “Mai Majuwa-Mai Pokhari-Dhara Pani, 4 Dec 1963, *H. Hara* et al.” (TI, photo).

### Selaginella pallida

Taxon classificationPlantaeSelaginellalesSelaginellaceae

Spring

01F469CC-86B4-5B34-9AF2-5A8D72DA1AB5

**Figure 3.**
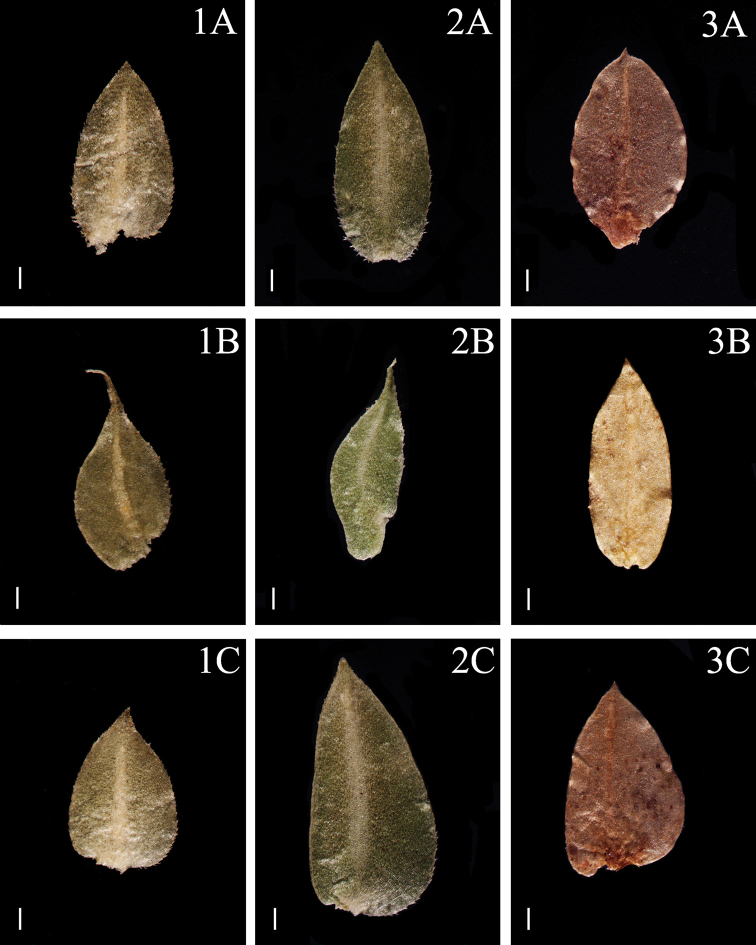
Morphological diversity of the leaves of Nepalese *Sealginella* species **1A–C***S. pallida* (*Nakaike 3740*, PE) **2A–C***S. remotifolia* (*Nakaike 3522*, PE) **3A–C***S. semicordata* (*Jenkins* s.n., PE). A – Axillary leaves, B – Dorsal leaves, C – Ventral leaves. Scale bars: 0.2 mm.

Figs 3 (1A–C), 9E , 18

Selaginella pallida Spring, Bull. Acad. Roy. Bel. 10: 234. 1843; Iwatsuki 1988; Thapa 2002; Fraser-Jenkins et al. 2015; Fraser-Jenkins et al. 2017; Zhang 2018.
≡ Lycopodium pallidum Hook. & Grev., Bot. Misc. 2: 389. 1831, nom. illeg.
≡ Selaginella plumosa var. pallida (Spring) Baker, J. Bot. 21: 145. 1883. **Type.** (lectotype, designated by Fraser-Jenkins et al. 2017) NEPAL. Nepal, [The Hon. E. Gardner for] *N. Wallich*, [c. 1817], Herb. Rudge (BM).
= Lycopodium pallidum Beyr. ex Gaudich., Voy. Uranie, Bot. pt. 7: 285. 1828, nom. illeg.
= Lycopodium tenellum D. Don, Prodr. Fl. Nepal.: 18 (1824), non (P. Beauv) Desv. ex Poir. (1814). **Type.** NEPAL. Nepalia, *Wallich* (?).
= Selaginella nepalensis Spring, Bull. Acad. Roy. Sci. Bruxelles 10(1): 234. 1843; Dixit 1992. **Type.** NEPAL. *Wallich* (?).
= Selaginella bomiensis Ching & S.K. Wu, Fl. Xizang. 1: 25. 1983. **Type.** CHINA. Xizang, Bomi (Tungmai), in rupibus, alt. 2000 m., *Y.T. Chang* et al. *881* (holotype: PE).

#### Description.

Stems 30–50 cm, long creeping. Rhizophores at intervals throughout length of main stem, borne on ventral side in axils of branches. Main stems branched throughout, pinnately branched, 1.2–2.0 mm in diam. in lower part. Axillary leaves ovate, 1.2–1.5 × 0.6–0.8 mm, dilated at base, dentate-serrulate, margin entire or upper part subentire, apex acuminate. Ventral leaves ovate, 2.0–2.8 × 1.1–1.3 mm, basiscoic base ovate, margin slightly dentate, aroscopic base dilated at base, margin dentate-serrulate, apex acuminate. Dorsal leaves ovate, 1.6–2.1 × 1.0–1.2 mm, in base cordate, margin serrulate-denticulate, falcate, apex acuminate-aristate. Strobili solitary, terminal, compact, 5–12 × 1–2 mm. Sporophylls monomorphic, ovate, acuminate, margin denticulate, sub-pellucid, apex acuminate. Megaspores light yellow, surface verrucate; microspores red, surface smooth, with echinae.

#### Ecology.

Forming dense mats on vertical banks in shade. Alt. 500–2200 m.

#### Distribution in Nepal.

C, E.

Nepalese threatened status: not available data.

#### General distribution.

BHUTAN, CHINA (Xizang). INDIA (Himachal Pradesh, Manipur, Meghalaya, Uttarakhand).

#### Chromosome number.

x=10, 2n=c. 20 (Fraser-Jenkins and Matsumoto 2015).

Selected specimens examined:

**C Nepal: NUWAKOT**: “Dhunche-Singum Compa, alt. c. 2200–3000 m, 18 Oct 1979, *T. Nakaike 238*” (PE).

**DHADING**: “Rithey Pani (between Mugling and Pokhara), alt. c. 500 m, 27 Sep 1986, *T. Nakaike 1948*” (PE 01622281);

**KASKI**: “Dhampus, Pokhara, alt. 1000–1500 m, 10 Nov 1988, *T. Nakaike 3781*” (PE); Karanha, near Pokhara, alt. c. 900 m, 9 Nov 1988, *T. Nakaike 3740*” (PE); “Pokhara, alt. 900 m, 18 Jun 1967, *H. Kanai* et al. *26128*” (KYO, photo).

**TANAHUN**: “Tanahun District, c. 6 km S of Damauli, E of Pokhara, W of Mugling and Anbu Khaireni, Rocks and forested slope below waterfall, next gorge, c. ½ km above Chowti Bara Temple gorge, 23 Mar 1997, *C.R. Fraser-Jenkins 25327* (*FN 1306*)” (US, photo).

**PALPA**: “Bategora, between Butwal and Pokhara, alt. c. 700 m, 9 Nov 1988, *T. Nakaike 3737*” (PE).

**KATHMANDU**: “Phulchoki, south of Kathmandu, on bank along path in shade, alt. c. 2200 m, alt. 2200–2700 m, 15 Jul 1972, *H. Hara*, *K. Iwatsuki* et al. *725556*” (KYO, photo); “Sundarijal-Mulkharka, alt. c. 1600 m, 4 Nov 1979. *T. Nakaike 418*” (PE); “Gokarna Ban, alt. c. 1370 m, 12 Oct 1979, *T. Nakaike 130*” (PE); “Tare Bhir, alt. c. 1400–1900 m, 4 Oct 1979, *T. Nakaike 59*” (PE); “Jamachowk, alt. c. 1500 m, 1 Oct 1986, *T. Nakaike 2169*” (PE); “Nagarjun, alt. c. 1400 m, 26 Aug 1986, *T. Nakaike 1092*” (PE); “l.c. *T. Nakaike 1124*” (PE); “l.c. *T. Nakaike* 1138” (PE); “Dakshin Kali, alt. c. 1500 m. 13 Sep 1986. *T. Nakaike 1438*” (PE); “Chandragiri, alt. 1600–2000 m, 9 Oct 1986, *T. Nakaike 2491*” (PE); “Gokarna Ban, Kathmandu, alt. c. 1350 m, 29 Oct 1988, *T. Nakaike 3587*” (PE); “first forested damp stream gully above road, 6 km N from Pharping Bazar, N of Bansbari, 1 km S of Chalankhel, NE side of Neipane Dara (hill), on W side of Bagmati River, c. 10 km S of Kathmandu on road to Dakshin Kali temple, 20 Jul 1996, *C.R. Fraser-Jenkins* et al. *24100* (*FN 78*)” (US, photo).

**BHAKTAPUR**: “Changu Narayan, alt. 1400–1500 m, 22 Sep 1986, *T. Nakaike 1801*” (PE); “Sankhu, alt. c. 1400 m, 24 Aug 1986, *T. Nakaike 1061*” (PE 01622270); “Nagarkot, alt. c. 1800 m, 16 Sep 1986, *T. Nakaike 1526*” (PE);

**KAVREPALANCHOK**: “Panauti, alt. c. 1400 m, 4 Oct 1986, *T. Nakaike 2373*” (PE).

**LALITPUR**: “Phulchoki, south of Kathmandu, alt. 2200–2700 m, on bank along path in shade, alt. c. 2200 m, 15 Jul 1972, *H. Hara* et al. *725556*” (TI); “Godawari (1600)–Phulchauki (2500m), alt. 1600–2500 m, 26 Jun 1967, *H. Hara* et al. s.n.” (TI, photo).

**RAMECHAP**: “Between Bhandar and Kenja, alt. 1700–2100 m, 7 Oct 1988, *T. Nakaike 3166*, *3167*” (PE);

**DOLAKHA**: “between Jiri and Sivalaya, alt. 1800–2000 m, 5 Oct 1988, *T. Nakaike 3088*” (PE); “Between Sivalaya and Jiri, Dolakha, alt. 1800–2000 m, 24 Oct 1988, *T. Nakaike 3526*” (PE).

**E Nepal: BHOJPUR**: “en route from Phedi to Sagangma, Along Irkhua khola, subtropical semievergreen forest zone, on the mossy rock, alt. 1160 m, 27 Jun 1978, *H. Tabata* et al. *10989*” (PE); Dingla 1000 m-Doban 800 m, on rather dry bank of part in shade, alt. 800–1000 m, 2 Jul 1972, *H. Kanai* et al. *725457*” (TI, photo; KYO, photo);

**SANKHUWASABHA**: “Tumulingtar-Khandbari, alt. 450–1150 m, 26 Jul 1977, *H. Ohashi* et al. *771532*” (TI, photo).

**PANCHTAR**: “Yatkin-Akasay-Batasay, 30 Nov 1963, *H. Hara* et al.” (TI).

### Selaginella remotifolia

Taxon classificationPlantaeSelaginellalesSelaginellaceae

Spring

FC4BAD81-6D4D-596E-817E-1370815BFD8C

Figs 3 (2A–C), 9F , 19

Selaginella remotifolia Spring, in Miq. Pl. Jungh. 3: 276. 1854; Zhang 2004; Zhang et al. 2013; Fraser-Jenkins et al. 2015; Fraser-Jenkins et al. 2017.
≡ Lycopodioides remotifolia (Spring) H.S. Kung, Fl. Sichuanica 6: 65, pl. 19. 1988 **Type.** INDONESIA. Sumatrae regionem sylvaticum prov. Angkolae superioris, alt. 1–3000 ft., *F.W. Junghuhn* (holotype: L).
= Selaginella involucrata Warb., Monsonia 1: 113, n. 28. 1900. **Type.** INDONESIA. Java. *Forbes n. 1034* (syntype: B [20 0154220]), INDONESIA. Preanger, Mt. Tilu bei Pentalengan. *Warburg 3484* (syntype: B [20 0154212]).
= Selaginella japonica Miq., Ann. Mus. Bot. Lugduno-Batavi 3(6): 185. 1867. **Type.** JAPAN. Detexit Keiske in prov. Owari (syntype: L [0059796]), *Siebold* et *Textor* etiam legerunt (syntypes: L [0052424], [0052430]).

#### Description.

Plants 15–45 cm, creeping, fertile branches erect. Rhizophores at intervals throughout length of creeping stem and branches, on dorsal side in axils of stem branches. Main stems branched above at base, 0.5–1.5 mm in diam. in lower part. Stems oval or terete, sulcate, glabrous, with single vascular bundle. Axillary leaves ovate-lanceolate or elliptic, 1.4–2.4 × 0.5–1.2 mm, base cuneate, margin slightly denticulate, apex slightly obtuse. Ventral leaves spreading, ovate-lanceolate, 1.8–3(–3.6) × 0.8–1.4(–1.7) mm, base rounded, acroscopic base not overlapping on stem and branches, margin minutely denticulate or subentire, apex acute. Dorsal leaves elliptic-lanceolate or ovate-elliptic, 1.4–2(–2.8) × 0.4–0.9(–1.2) mm, base uniauriculate, margin subentire or minutely denticulate, apex long acuminate. Strobili solitary, terminal and lateral to branches, compact, tetragonal, 3.5–6 × 1–3 mm. Sporophylls monomorphic, ovate-lanceolate, carinate, margin denticulate, apex acuminate. Megaspores gray-white, surface irregular reticulum; microspores pale yellow, surface with triangular and striped spines.

#### Ecology.

Terrestrial, evergreen, sub-open forest banks, previously overlooked or on slopes in shade. Alt. 1800–2650 m.

#### Distribution in Nepal.

C, E.

Nepalese threatened status: EN (Fraser-Jenkins et al. 2015).

#### General distribution.

CHINA (Chongqing, Fujian, Guangxi, Guizhou, Hubei, Hunan, Jiangxi, Sichuan, Taiwan, Yunnan, Zhejiang), NE INDIA, INDONESIA (Sumatra), JAPAN, PHILIPPINES.

#### Chromosome number.

Not available data.

Selected specimens examined:

**C Nepal: DOLAKHA**: “between Kenja and Sivalaya, Dolakha, alt. c. 2300 m, 23 Oct 1988, *T. Nakaike 3522*” (PE01722894).

**KASKI**: “Between Landrung and Potana, alt. 1900 m, 3 Aug 1983, *H. Tabata* et al. *19149*” (KYO, photo).

**RAMECHAP**: “between Sivalaya and Bhandar, alt. 1800–2500 m, 6 Oct 1988, *T. Nakaike 3137*” (PE).

**E Nepal: TEHRATHUM**: “Chauki (2650 m)–Tute (2480m)–Basantapur (2300m), 27°12'35"N, 87°28'01"E–27°07'00"N, 87°26'00"E, 17 Aug 1999, *K. Fujikawa* et al.” (PE01722895); “Tinjure-Chauke, alt. 2700 m, 7 Aug 1972, *H. Kanai* et al. *725131*” (E00659376; KYO, photo); “Chauki (2650 m)–Tute (2480 m)–Basantapur (2300 m), Tinjure, alt. 2800 m, 27°12'35"N, 87°28'01"E; 27°07'00"N, 87°26'00"E, 17 Aug 1999, *M. Tateno* et al. *9955140*” (KATH).

**SOLUKHUMBHU**: “Dorange, Junbesi, Solukhumbu, alt. c. 2500 m, 20 Oct 1988, *T. Nakaike 3472*” (PE).

**ILAM**: “Mai Pokhari, 27°00'N, 87°57'E, alt. 2000 m, 28 Sep 1971, *J.F. Dobremez* DBR NEP *1233*” (E00670678).

### Selaginella semicordata

Taxon classificationPlantaeSelaginellalesSelaginellaceae

(Wall. ex Hook. & Grev.) Spring

EFBCBB56-B1CE-57E7-BB80-AAAC0C74810E

Figs 3 (3A–C), 9G , 20

Selaginella semicordata (Wall. ex Hook. & Grev.) Spring, Fl. Bras. 1(2): 122. 1840; Dixit 1992; Thapa 2002; Singh and Panigrahi 2005; Fraser-Jenkins et al. 2015; Fraser-Jenkins et al. 2017.
≡ Lycopodium semicordatum Wall. ex Hook. & Grev., Bot. Misc. 2: 396. 1831.
(Lycopodium semicordatum Wall., Cat. n. 126, p. p. 1821, nom. nud.).
≡ Lycopodioides semicordata (Wall. ex Hook. & Grev.) Kuntze, Revis. Gen. Pl. 1: 827. 1891. **Type.** (lectotype, designated by Fraser-Jenkins et al. 2017) INDIA. N. E. India, Meghalaya: montes Sylhet vicinae. *M.R. S[mith*]. [*Wallich List no*.] *126.3* (E; isolectotype: K–W).
(Selaginella burghallii R. Sim., Priced Cat. Ferns 6: 61. 1859, nom. nud.)

#### Description.

Stems to 150 cm, creeping, slender. Rhizophores at intervals throughout length of creeping stem and branches, on dorsal side in axils of stem branches. Stem slender, sulcate. Main stems, branched throughout, arise alternately from base of plant, branches short, pinnately, flabellate, distant located. Axillary leaves oblong-lanceolate, 2.1–2.6 × 1.3–1.8 mm, base rounded-cuneate, apex truncated; on branches, obovate, 1.3–2.0 × 0.6–0.9 mm. Ventral leaves oblong-lanceolate, 1.9–2.5 × 0.7–1.2 mm, rounded at base, acroscopic base slightly enlarged, not overlapping stem and branches, margin in apex part suberose, apex subobtuse to subacute. Dorsal leaves oblong, 1.5–1.8 × 0.5–0.7 mm, imbricate, in basal part oblique, margin entire to minutely denticulate at apex, apex shortly cuspidate. Strobili solitary, terminal, tetragonal, compact, 5.0–15 × 1.5–2.3 mm. Sporophylls monomorphic, 1.6–2 × 0.8–1 mm, ovate, keeled, margin entire, apex acute. Megaspores dark-brown, surface verrucate; microspores pale, surface exine with white, translucent wing-like perispore supported with hook-like structure.

#### Ecology.

Terrestrial, growing in paddy field or marshland or shady areas among the grasses with abundant water content. Restricted low altitude species of wet. Alt. 100–150 m.

#### Distribution in Nepal.

C, E.

Nepalese threatened status: EN (Fraser-Jenkins et al. 2015).

#### General distribution.

BANGLADESH, INDIA (Assam State, Bihar, Meghalaya, Mizoram, Nagaland, Tripura, West Bengal), MYANMAR.

#### Chromosome number.

Not available data.

Selected specimens examined:

**E Nepal: JHAPA**: “Range Danda, common on shady banks, alt. 100 m, 24 Jan 2003, *N. Thapa* et al. *2016*” (KATH).

### Selaginella helvetica

Taxon classificationPlantaeSelaginellalesSelaginellaceae

(L.) Spring

3356465B-609E-5494-8B25-21E9B5BE377F

**Figure 4.**
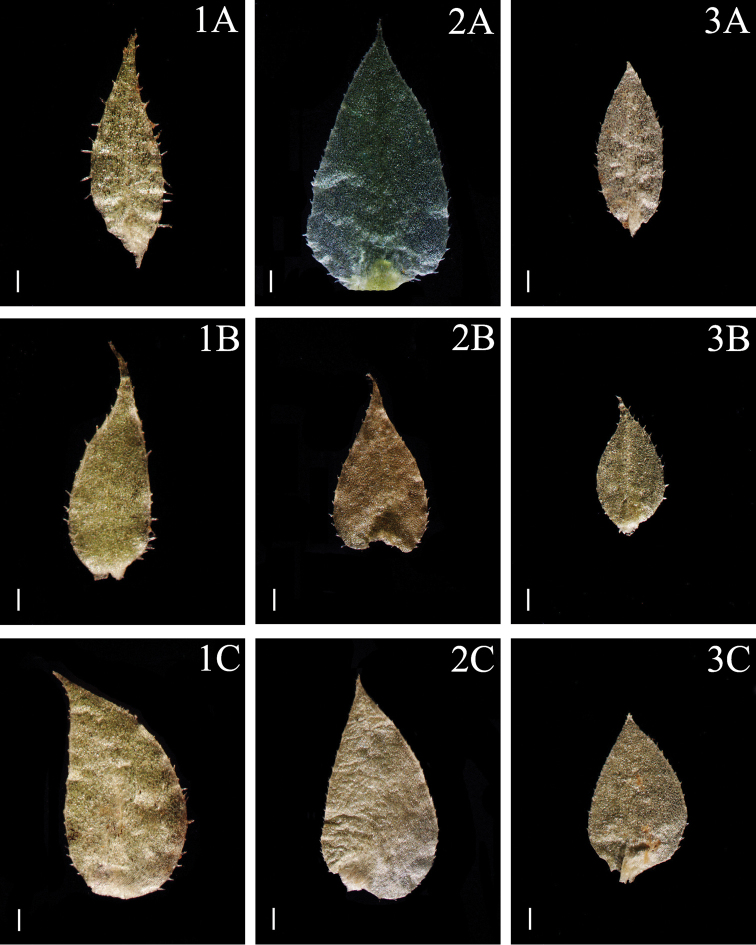
Morphological diversity of the leaves of Nepalese *Sealginella* species **1A–C***S. helvetica* (*Zhang 0638*, PE) **2A–C***S. pallidissima* (*Zhang 2746*, PE) **3A–C***S. laxistrobila* (*Nakaike 1319*, PE). A – Axillary leaves, B – Dorsal leaves, C – Ventral leaves. Scale bars: 0.2 mm.

Figs 4 (1A–C), 9H , 21

Selaginella helvetica (L.) Spring, Flora 21(1): 149. 1838; Zhang 2004; Zhang et al. 2013; Fraser-Jenkins et al. 2015; Fraser-Jenkins et al. 2017.
≡ Lycopodium helveticum L., Sp. Pl. 2: 1104. 1753.
≡ Bernhardia helvetica (L.) Gray, Nat. Arr. Brit. Pl. 2: 23. 1821.
≡ Diplostachyum helveticum (L.) P. Beauv., Prodr. Aethéogam.: 107. 1805.
≡ Heterophyllum helveticum (L.) Hieron. ex Börner, Fl. Deut. Volk 110, f. 29. 1912.
≡ Lycopodioides helvetica (L.) Kuntze, Rev. Gen. Pl. 1: 826. 1891.
≡ Selaginella helvetica (L.) Link, Fil. Spec. 159. 1841, later isonym.
≡ Stachygynandrum helveticum (L.) P. Beauv. ex J. St.-Hil., Expos. Fam. Nat. 1: 39. 1805. **Type.** (lectotype, designated by Troia and Greuter 2015) Florentiae, et in Taurero Rastadiensi, Herb. Burser XX: 46 (UPS).

#### Description.

Stems 5–15(–25) cm, short-creeping, fertile stems erect. Rhizophores at intervals throughout length of creeping stem and branches, borne on ventral side in axils of branches.Main stems branched throughout, 0.2–0.4 mm in diam. in lower part. Stems stramineous, stem angulate, sulcate, primary leafy branches 2–5 pairs, simple, forked, or once pinnately branched, branchlets sparse, branches arranged on main stem 2–3 cm apart. Axillary leaves ovate-lanceolate or elliptic, 1.4–1.6 × 0.4–0.8 mm, base exauriculate, margin ciliolate. Ventral leaves oblong-ovate or broadly ovate, 1.6–2 × 0.8–1.2 mm, leaves on branches spreading or slightly deflexed, basiscopic margin ciliolate, acroscopic base enlarged, broader, overlapping stem and branches, margin ciliolate, apex acute or aristate, often bent upward. Dorsal leaves symmetrical or not, ovate or ovate-lanceolate, 1.2–1.6 × 0.5–0.8 mm, base obtuse, margin ciliolate, apex long acuminate or aristate, often reflexed. Fertile branches erect, 3–6 cm. Strobili solitary or forked, terminal, lax or lax in lower partion, and compact in upper part, cylindric, 12–35 × 2–4 mm. Sporophylls unlike sterile leaves or similar, margin ciliolate, apex long acuminate. Megaspores orange or yellowish orange, surface verrucate; microspores orange or orange-red, surface verrucate.

#### Ecology.

On moss-covered cliffs, in rock crevices, on damp shaded banks in mixed forests, to mossy areas. Alt. 2300–4000 m.

#### Distribution in Nepal.

W, C.

Nepalese threatened status: NT (Fraser-Jenkins et al. 2015).

#### General distribution.

EUROPE, RUSSIA, JAPAN, KOREA, MONGOLIA, CHINA (S Gansu, Hebei, Heilongjiang, Jilin, Liaoning, Nei Mongol, Qinghai, Shaanxi, Shandong, Sichuan, Xizang, Yunnan), INDIA (Himachal Pradesh, Uttarakhand, West Bengal).

#### Chromosome number.

2n=18 (Manton 1950; Jermy et al. 1967).

Selected specimens examined:

**W Nepal: DOLPA**: “between Rohagaon and Lulo Khola, Suli Gad, moist shady bank in mixed forest, growing among moss, alt. 10,000 ft, 15 Sep 1952, *O. Polunin*, *W.R. Sykes & L.H.J. Williams 3403*” (PE; TI, photo; E, photo; US, photo; KYO, photo).

### Selaginella pallidissima

Taxon classificationPlantaeSelaginellalesSelaginellaceae

Spring

4803B23F-4383-52A2-B067-7C43CFA502AA

Figs 4 (2A–C), 9I , 22

Selaginella pallidissima Spring, Bull. Acad. Roy. Sci. Bruxelles 10: 231. 1843; Alston 1945; Iwatsuki 1975; Iwatsuki 1988; Dixit 1992; Thapa 2002; Zhang 2004; Fraser-Jenkins et al. 2015; Fraser-Jenkins et al. 2017. **Type.** INDIA. *V. Jacquemont 2331* (holotype: P [00523060]), Himalaya, alt. 3000 m. *V. Jacquemont 2331* (isotype: K [001067411]).
– Selaginella integerrima sensu Strachey, Gaz. North-West Prov.: 66. 1882, non Spring, 1850.

#### Description.

Stems 15–35 cm, creeping. Rhizophores at intervals throughout length of main stem, borne on ventral side in axils of branches. Main stems branched throughout, pinnately branched, 0.3–0.5 mm in diam. in lower part. Main stems stramineous or reddish, angulate, sulcate. Axillary leaves ovate, 2–3 × 1–1.5 mm, base subcordate, margin minutely denticulate, apex acuminate. Ventral leaves ovate or ovate-triangular, 1.8–3.2 × 1.1–1.8 mm, basiscopic base rounded, margin denticulate, acroscopic base enlarged, overlapping stem and branches, margin denticulate or ciliolate in basal portion, apex acute. Dorsal leaves ovate or ovate-lanceolate, 1.5–2.2 × 0.6–1.3 mm, base subcordate, margin minutely denticulate or ciliolate, apex acuminate. Strobili solitary or rarely paired, terminal, 6–10 × 1–2 mm. Sporophylls dimorphic, dorsal sporophylls ovate, oblique, in base subcordate, margin shortly ciliolate or denticulate, apex acute, ventral sporophylls ovate or oblong-ovate, not carinate, margin denticulate. Megaspores sulfur colored or yellowish orange, surface verrucate, microspores orange-red, surface covered with spinulose microsculpture.

#### Ecology.

Terrestrial or epilithic, on steep, open, rather dry banks among grasses, seasonally green. Alt. 2700–3300 m.

#### Distribution Nepal.

W, C, E.

Nepalese threatened status: LC (Fraser-Jenkins et al. 2015).

#### General distribution.

CHINA (Sichuan, Yunnan, Xizang), INDIA (Uttar Pradesh, Himachal Pradesh).

#### Chromosome number.

Not available data.

Selected specimens examined:

Nepal: “Manglui Banjuang, alt. 2800 m, 28 Jul 1972, *A. Maire* AMA *443*” (E00670584).

**W Nepal: JUMLA**: “Ghurchi Lekh, near Chautha, alt. 10,000 ft, 28 Aug 1952, *O. Polunin*, *W.R. Sykes & L.H.J. Williams 3068*” (E, photo; US, photo).

**DOLPA**: “Rohagaon, Suli Gad, alt. 9500 ft, 13 Sep 1952, *O. Polunin*, *W.R. Sykes & L.H.J. Williams 3364*” (E, photo; US, photo; KYO, photo); “l.c. *3365*” (KYO, photo); “Near Hurta, Bhalu Lekh, alt. 9000 ft, 5 Aug 1952, *O. Polunin*, *W.R. Sykes & L.H.J. Williams 3178*” (E; KYO, photo).

**DOTI**: “Dotu-Siligarhi, foliage red brown, in rocky stream bed, alt. 4500 ft, 1 Apr 1967, *N. Ecker-Racz*” (US, photo).

**C Nepal: MUSTANG**: “Tukucha, Kali Gandaki, alt. 10500 ft, 13 Jun 1954, *J.D.A. Stainton*, *W.R. Sykes* and *L.H.J. Williams 1110*” (E, photo).

### Selaginella laxistrobila

Taxon classificationPlantaeSelaginellalesSelaginellaceae

K.H. Shing

346FE698-46C1-554B-B631-4DD516AFA39F

Figs 4 (3A–C), 10A , 23

Selaginella laxistrobila K.H. Shing, Acta Phytotax. Sin. 31: 569. 1993; Zhang 2004; Zhang et al. 2013. **Type.** CHINA. Sichuan: Kanding, jieba, Sewuroug, alt. 3350 m., ad clivum australem in sylvis Quercorum, 2 Aug 1981. *S.S. Kung 6067* (holotype: PE).

#### Description.

Stems 1–6 cm, with creeping main stems and few upright stems over a short distance. Rhizophores restricted to lower part of stem. Main stems branched from near base upward, 0.2–0.4 mm in diam. in lower part. Stems stramineous, angulate, sulcate. Axillary leaves elliptic, 1–1.8 × 0.3–0.7 mm, base exauriculate, margin slightly denticulate, apex acute, not aristate. Ventral leaves ovate-triangular, 1.8–2.3 × 0.8–1.2 mm, acroscopic base enlarged, broader, slightly overlapping stem and branches, margin ciliolate, apex acute. Dorsal leaves ovate, 1.2–1.8 × 0.6–0.8 mm, base subcordate or obtuse, margin ciliolate, apex acuminate. Sporophylls dimorphic, similar to sterile leaves in form and arrangement. Strobili solitary or forked, terminal, lax, dorsiventrally complanate, 10–20 × 3–5 mm. Ventral sporophylls ovate, margin shortly ciliolate, apex acuminate; dorsal sporophylls ovate-lanceolate, margin shortly ciliolate, apex acuminate. Megaspores orange or yellowish orange, surface verrucate; microspores orange, surface verrucate.

#### Ecology.

Terrestrial, evergreen, under shrubs in damp places mixed forests, on rocks, soil banks. Alt. 2650–3200 m.

#### Distribution in Nepal.

C, E.

Nepalese threatened status: not available data.

#### General distribution.

CHINA (Sichuan, Yunnan), INDIA.

#### Chromosome number.

Not available data.

Selected specimens examined:

**C Nepal: RASUWA**: “between Ghora Tabela and Lama Hotel. 2 Sep 1986, *T. Nakaike 1319*” (PE).

**E Nepal: SOLUKHUMBU**: “between Goem and Junbesi, Solukhumbu, alt. 2650–3200 m, 9 Oct 1988, *T. Nakaike 3256*” (PE).

### Selaginella bisulcata

Taxon classificationPlantaeSelaginellalesSelaginellaceae

Spring

D22FB298-7D6D-576E-855C-88AAD1BA1BD8

**Figure 5.**
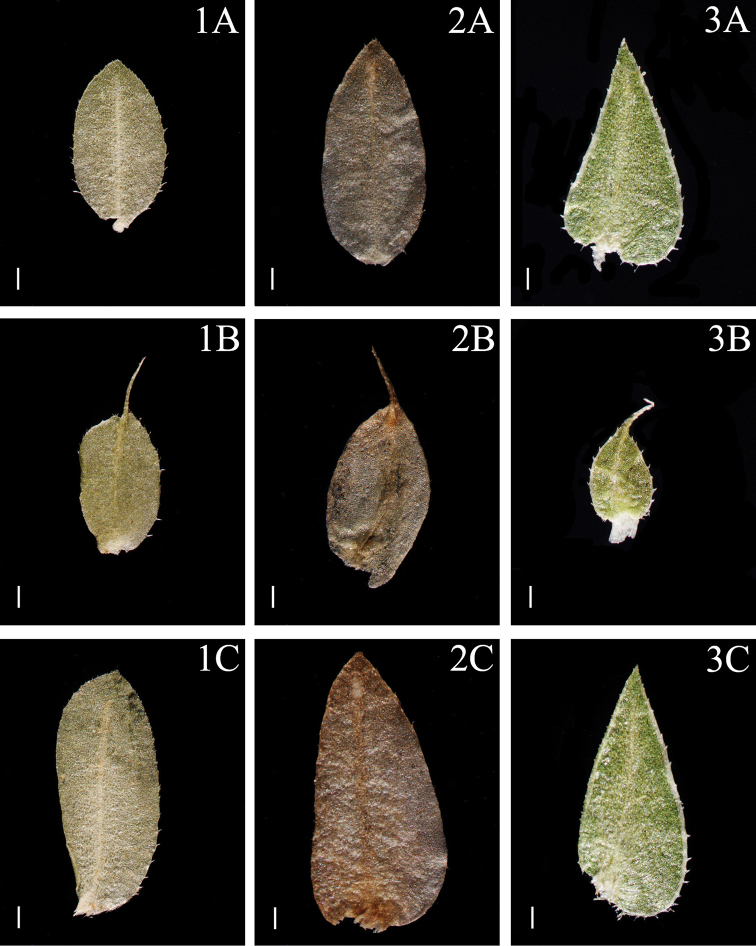
Morphological diversity of the leaves of Nepalese *Sealginella* species **1A–C***S. bisulcata* (*Nakaike 3786*, PE) **2A–C***S. pennata* (*Nakaike 3507*, PE) **3A–C***S. chrysocaulos* (*Nakaike 1058*, PE). A – Axillary leaves, B – Dorsal leaves, C – Ventral leaves. Scale bars: 0.2 mm.

Figs 5 (1A–C), 10B , 24

Selaginella bisulcata Spring, Mém. Acad. Roy. Sci. Belgique 24: 259. 1850; Iwatsuki 1975; Iwatsuki 1988; Dixit 1992; Thapa 2002; Zhang 2004; Zhang et al. 2013; Fraser-Jenkins et al. 2015; Fraser-Jenkins et al. 2017.
≡ Lycopodioides bisulcata (Spring) Kuntze, Revis. Gen. Pl. 2: 826. 1891, as “*bisulcatum*”. **Type.** INDIA. N.E. India, Meghalaya, Khasia, *W. Griffith* (mislabelled as “Gorval”, i.e. Garhwal, Uttarakhand) (holotype: K).
= Selaginella bisulcata var. spinulosa Spring, Mém. Acad. Roy. Sci. Belgique 24(2): 260. 1850. **Type.** INDIA. Assam, *Griffith* s.n. (holotype: K).

#### Description.

Stems 20–45 cm, creeping. Rhizophores at intervals throughout stems, located on ventral side in axis branches. Main stems branched from near base upwards, in basal part main stem 1.2–1.8 mm in diam. Main stems subquadrangular, sulcate, branched throughout their length, primary leaves branches arranged 5–8 pairs. Axillary leaves elliptic, 3–4.6 × 1.1–1.6 mm, base exauriculate, margin denticulate or sparsely ciliolate. Ventral leaves asymmetrical, slightly ascending or spreading or deflexed, oblong, 3.2–5 × 1.2–2 mm, apex apiculate, in base margin entire or subentire, denticulate at apex, leaves not overlapping stem and branches, margin ciliolate or denticulate in basal and apical portions, entire in middle. Dorsal leaves asymmetrical, 1–2.4 × 0.6–1.5 mm, base obliquely cuneate, margin sparsely ciliolate, apex mucronate or aristate with arista curved, up to 1/2–4/5 as long as leaf, 0.4–0.8 mm. Strobili solitary, terminal, compact, 6–10 × 3.5–5.5 mm. Sporophylls dimorphic, ventral sporophylls ovate-lanceolate or oblong-ovate, in base dilated, margin ciliolate or lacerate-ciliolate; dorsal sporophylls oblong-lanceolate, carinate, margin ciliolate, apex acuminate or aristate, with sporophyll-pteryx incomplete and ciliolate. Megaspores white-brown, surface smooth; microspores orange, surface verrucate.

#### Ecology.

Evergreen, often in open dry slope areas, or in a little shade in light forest. Alt. 1500–2700 m.

#### Distribution in Nepal.

W, C, E.

Nepalese threatened status: not available data.

#### General distribution.

BHUTAN, CHINA (Sichuan, Yunnan), INDIA (Assam State, Sikkim, Manipur, Meghalaya, Nagaland, West Bengal), INDONESIA, MYANMAR, THAILAND, VIETNAM.

#### Chromosome number.

not available data.

Selected specimens examined:

**C Nepal: NUWAKOT**: “Chandragiri, near Thankot, Kathmandu, Nuwakot, c. 2000 m, 19 Nov 1986, *T. Nakaike 3855*” (PE 01622152).

**KATHMANDU**: “Bagmati Zone, Kathmandu Distr., Second khola from the W. side of valley, at edge of low forest, c. 150 m. above and ¼ km SE of Bhangeri, above Gagal Phedim, N.W. of Sankhu, N. E. Kathmandu, 2 Oct 2001, *C.R. Fraser-Jenkins*, *G.B. Tamang 29346* (*FN 5321*)” (US, photo); “Bhangeri, c. 1800–2100 m, 2 Oct 1986, *T. Nakaike 2267*” (PE 01622219); “Bhangeri, c. 1800–2100 m, 2 Oct 1986, *T. Nakaike 2306*” (PE 01622220).

**KASKI**: “Dhampus, Pokhara, 1000–1500 m. 10 Nov 1988, *T. Nakaike 3786*” (PE 01622148); “Panchase Lekh (Kaski Distr.), n. 834282, alt. 2350 m, 12 Dec 1973, *D.P. Joshi*, *M.M. Amatya 73/1170*” (KATH); “Pathana (Dhampus)–Tolka, alt. 1850–2050 m, 8 Jul 1983. *H. Ohba* et al. *8330208*” (TI, photo); “Banjan (2035m)–Mt. Panchase (2500m), alt. 2120 m, 28°13'03"–15°12'N, 83°49'56"–47°54'E. 10 Sept 1999, *M. Mikage* et al. *9965087*” (TI, photo).

**DOLAKHA**: “Tandi, alt. 1500 m. 9 Sep 1954, *A. Zimmermann 1132*” (KYO, photo).

**E Nepal: ILAM**: “near Ilam, Ilam, c. 1500–2000 m, 5 Nov 1986, *T. Nakaike 3646*” (PE 01622149); “Ilam District, wooded slopes with *Cryptomeria* trees, c. ½ km W of Pashupatinagar, above main road to Ilam, ENE of Ilam, 8 Sep 2001, *C.R. Fraser-Jenkins*, *G.B. Tamang 5405*” (US, photo); “Ilam Distr.: In Oak-forest on slopes above streams, between Chitregaon and Manebhanjyang, c. 4–5 km NE of Pashupatinagar, on footpath to Manebhanjyang, near Indian border, NE of Ilam, 23 Oct 2001, *C.R. Fraser-Jenkins 29608* (*FN 5583*)” (US, photo); “Mai Pokhari, 27°00'N, 87°57'E, alt. 2000 m, 28 Sep 1971, *J.F. Dobremez* DBR NEP *1233*” (E00670678); “Mai Majuwa-Mai Pokhari-Dhara Pani, 4 Dec 1963, *H. Hara* et al.” (TI, photo; KYO, photo); “Ilam, 26°57'N, 87°57'E, alt. 1450 m, 28 Sep 1971, *J.F. Dobremez* DBR NEP *1202*” (E00670573); “Ilam District, Pashupatrinagar, alt. 2300 m, 8 Oct 2001. *C.R. Fraser-Jenkins* & *G.B. Tamang 29430* (*FN 5405*)” (KATH).

**TAPLEJUNG**: “Dumhan, by the Tamur River, alt. 700 m, 31 Sep 1963, *G. Murata*, *M. Togashi*, *T. Tuya*” (TI, photo).

**SOLUKHUMBU**: “(Solukhumbu Distr.)–Janakpur Zone (Ramechhap Distr.), Namikhil (2300 m)–Chamare (1900 m)–Likhu (a bridge) (1550 m)–Bhandar (2300 m), alt. 1550–2300 m, 27°33'N, 86°23'E –27°34'N, 86°20'E, 10 Sep 1985, *H. Ohba* et al. *8581505*” (TI, photo).

### Selaginella pennata

Taxon classificationPlantaeSelaginellalesSelaginellaceae

(D. Don) Spring

6D670225-0B3D-506C-AEFB-179EAEADC9A6

Figs 5 (2A–C), 10C , 25

Selaginella pennata (D. Don) Spring, Bull. Acad. Roy. Sci. Bruxelles 10: 232. 1843; Alston 1945; Iwatsuki 1975; Iwatsuki 1988; Dixit 1992; Thapa 2002; Zhang 2004; Zhang et al. 2013; Fraser-Jenkins et al. 2015; Fraser-Jenkins et al. 2017.
≡ Lycopodium pennatum D. Don, Prodr. Fl. Nepal.: 18. 1824.
≡ Lycopodioides pennata (D. Don) Kuntze, Revis. Gen. Pl. 1: 827. 1891. **Type.** (lectotype, designated by Alston 1945) NEPAL. «Lycopodium pennatum Don Prod. Fl. Nep., Napaul, *Dr. Buchanan*, type specimen of Lycopodium pennatum D. Don, det. A.H.G. Alston (BM).
= Selaginella suberosa Spring, Monogr. Lycop. 1: 253, no. 191. 1850. **Type.** INDIA. Hindustania superiori, Gorval [error for the Khasi Hills]. *Griffith* s.n. (holotype: K; isotype: P?).

#### Description.

Stems 15–35 cm, suberect. Rhizophores long, thick, restricted to basal and lower part of main stem. Main stems branched slightly above bottom of stem, not very regularly pinnately branched. Stems terete, not sulcate or sulcate in upper part. Axillary leaves ovate, 1.4–2.3 × 0.6–1.2 mm, base exauriculate, margin ciliolate in basal part, upward subentire, or with rarely cilia. Ventral leaves oblong or oblong-ovate, 1.6–3 × 0.7–1.4 mm, base rotundate, acroscopic base rounded, not overlapping stem and branches, margin sparsely shortly ciliolate, apex acute or apiculate. Dorsal leaves elliptic, sub-falcate, oblique, entire, margin rarely ciliolate, apex aristate with arista curved. Strobili solitary or pairs, terminal, compact, 4–10(–12) × 2.5–5 mm. Sporophylls dimorphic, dorsal sporophylls ovate-oblong, margin denticulate, apex acute, ventral sporophylls ovate, margin entire or denticulate, apex acute. Megaspores whitish, gray or dark brown, surface globose; microspores pale yellow, surface verrucate.

#### Ecology.

Terrestrial, seasonally green, in mixed forests on rather dry mountain slopes. Alt. 500–2400 m.

#### Distribution Nepal.

C, E.

Nepalese threatened status: not available data.

#### General distribution.

CHINA (Yunnan), INDIA (Assam, Manipur, Meghalaya, Sikkim, West Bengal), MYANMAR, THAILAND.

#### Chromosome number.

Not available data.

Selected specimens examined:

**C Nepal: SINDHUPALCHOK**: “Patibhanjyang to Talangmarang, terrestial growing on moist sandy cliff frequent, alt. 7010 to 7800 ft. (2135–2377 m), 22 Oct 1978, *V.L. Gurung*, *M. Gorkhali 78/680*” (KATH); “Manichur to Patibhanjyang, terrestial, growing on the shady moist place, common, alt. 7010–7800 ft. (2135–2377 m), 21 Oct 1978, *V.L. Gurung*, *M. Gorkhali 78/604 (a)*” (KATH).

**DHADING**: “Birjet, alt. 1620 m, on mossy rock. 4 Nov 1989. *N.P. Manandhar* 12962” (KATH).

**RASUWA**: “Mani gaon (on way to Ramche), terrestrial, grown on sandy and wetty slope by the way side abundant, alt. 1230 m, 29 Sep 1977, *Mrs. V.L. Gurung* et al. *77/600*” (KATH); “Mani gaon (on way to Ramche), terrestrial, grown on sandy and wetty slope by the way side abundant, alt. 1230 m, 29 Sep 1977, *Mrs. V.L. Gurung* et al. *77/601*” (KATH); “Rasuwa Distr.: in forest, Domen to Bompu, S. side of Langtang River, between Syabrubensi and bridge below Lama Hotel, lower Langtang Valley, alt. 1600–2200 m. 21 Aug 2001. *C.R. Fraser-Jenkins* & *G.B. Tamang* 29198 *(FN 5173)*” (US, photo).

**KATHMANDU**: “second Khola from the W. side of valley, at edge of low forest, c. 1500 m above and ¼ km S. E. of Bhangeri, above Gagal Phedim, N.W. of Sankhu, N. E. of Kathmandu, 2 Oct 2001, to *C.R. Fraser-Jenkins* & *G.B. Tamang 29345 (FN 5320)*” (US, photo).

**LAMJUNG**: “Phelingsanku, 28°13'N, 84°24'E, alt. 650 m, 25 Nov 1970, *J.F. Dobremez* DBR NEP *646*” (E00754783).

**KASKI**: “Dhampus, Pokhara, alt. 1000–1500 m, 10 Nov 1988, *T. Nakaike 3776*” (PE01622289); “Pokhara, on S side of Phewa Tal, c. 3–4 km. W of «Fishtail Lodge» Hotel, opposite Pokhara «Lakeside» town (Baidam). “Up forested khola at and shortly west of Anadu village. 24 Sep 1997. *C.R. Fraser-Jenkins 25566* (*FN 1544*)” (US, photo); “Pokhara to Hyenda, alt. 1000–1100 m, 20 Sep 1976, *Y. Suehiro 93*, *94*” (KYO, photo); “Naudanda, alt. 1300 m, 21 Sep 1976, *Y. Suehiro 2040*” (KYO, photo).

**TANAHUN**: “among boulders by stream in forest on W. side of Khane khola valley, between Dumrekharka village and W. part of Chimkeshwori Darrah(mountain) S. of Khanekhola village, c. 3 km W of Anbu Khaireni, W. of Mugling on Damauli and Pokhara, 7 Oct 2000, *C.R. Fraser-Jenkins 28630* (*FN 4605*)” (US, photo).

**KATHMANDU**: “Jarkini, alt. 1600–1700 m. 29 Sep 1986, *T. Nakaike 2041*” (PE 01622288); **RAMECHAP**: “between Bhandar and Kenja, alt. 2100–1700 m. 7 Oct 1988, *T. Nakaike 3201*” (PE 01622286);

**UDAYAPUR**: “vers le col de Sukhchauri, boises exposés vers l’est, 1000 m, 7 Nov 1954, *A. Zimmermann 2100*” (KYO, photo).

E Nepal:

**SANKHUWASABHA** “Simbu, 27°22'N, 87°47'E, alt. 1800 m, 05 Oct 1971, *J.F. Dobremez* DBR NEP *1337*” (E00754787).

**SOLUKHUMBU**: “Karodo, near Kenja, Solukumbu, alt. c. 1750 m, 22 Oct 1988, *T. Nakaike 3507*” (PE);

**PANCHTAR**: “Ektin, 27°12'N, 87°53'E, alt. 1500 m, 2 Oct 1971, *J.F. Dobremez* DBR NEP *1300 B*” (E00670574).

**MORANG**: “Chisapani, on the moist place, stem red rhizome long, alt. 600 m, 26 Sep 1971, *D.P. Joshi 28*” (KATH); “Chisapani, alt. 500 m, 26°50'N, 87°55'E, 26 Sep 1971, *J.F. Dobremez* DBR NEP *1169*” (E00670671).

### Selaginella chrysocaulos

Taxon classificationPlantaeSelaginellalesSelaginellaceae

(Hook. & Grev.) Spring

37705999-9582-5C52-A84B-97182C251BFB

Figs 5 (3A–C), 10D , 26

Selaginella chrysocaulos (Hook. & Grev.) Spring, Bull. Acad. Roy. Sci. Bruxelles 10(1): 232, no. 141. 1843; Iwatsuki 1975; Iwatsuki 1988; Dixit 1992; Thapa 2002; Zhang 2004; Zhang et al. 2013; Fraser-Jenkins et al. 2015; Fraser-Jenkins et al. 2017.
≡ Hook. & Grev., Bot. Misc. 2: 401. 1831.
≡ Lycopodioides chrysocaulos (Hook. & Grev.) H.S. Kung, Fl. Sichuanica 6: 78, pl. 24. 1988. **Type.** NEPAL. *N. Wallich List* n. *127* (holotype: K; isotype: E).
= Selaginella hypnoides Spring, Mém. Acad. Roy. Sci. Belgique 24(2): 101. 1850. **Type.** INDIA. Himalaya, *Jacquemont* n. *1041* (holotype: P [00523047]).
= Selaginella philippina var. khasiensis Baker, J. Bot. 22: 298. 1884. **Type.** INDIA. Mt. Khasia, *Griffith* s.n. (holotype: K?).
= Selaginella rosenstockii Hieron., Hedwigia 43: 22. 1904. **Type.** INDIA. India Orientalis: Simla in via Kangra, and Jammu and Kashmir, alt. s. m. inter 1000 et 3000 m. Jun.–Sept. 1856. *Schlagintweit* n. *13256* (syntypes: S [S–P–17948], B [200154162]); Himalaya loco accuratius non indicato, *Warburg 1005* (syntype: B [20 0154160]); Ny nee Jal, s.m. c. 3000 m., *Strachey*, *Winterbottom 9* (syntypes: B [20 0154159], [20 0154157]; Mussoorie, *Jameson* n. *582* partim (syntype: B [20 0154156]; Simla, Regio Temp., *T. Thomson* s.n. (syntype: B [20 0154158]).

#### Description.

Stems 5–25 cm, evergreen or seasonally green, erect, with elongate tuber at base. Rhizophores restricted to base of stem or borne in lower part. Main stems branched from near base or from lower part upward, in basal part main stem 0.5–1 mm in diam. Stems stramineous, terete or subquadrangular, primary leafy branches 6–12 pairs, forked or once or twice pinnately branched, branchlets sparse. Axillary leaves asymmetrical, narrowly ovate or narrowly elliptic, 2–3 × 1–1.4 mm, base exauriculate, in base margin ciliolate, apex blunt-acute. Ventral leaves asymmetrical, ovate-lanceolate, 1.4–2 × 0.8–1.4 mm, leaves on branches slightly ascending or spreading, margin sparsely minutely denticulate or ciliolate at base, apex acute. Dorsal leaves asymmetrical, narrowly ovate, 0.6–1 × 0.3–0.5 mm, base subcordate or obliquely cordate, carinate or not carinate, in basal part margin denticulate or ciliolate, apex acuminate or aristate. Strobili solitary, terminal, compact, 3–5 × 1–1.5 mm. Sporophylls slightly or strongly dimorphic, ventral sporophylls ovate, margin denticulate; dorsal sporophylls with sporophyll-pteryx incomplete and ciliolate, margin ciliolate. Megaspores yellowish, surface verrucate; microspores orange, surface verrucate.

#### Ecology.

On clay soil or on damp shaded banks in forest. Alt. 1400–2900 m.

#### Distribution in Nepal.

W, C, E.

Nepalese threatened status: not available data.

#### General distribution.

BHUTAN, CHINA (Guizhou, Sichuan, Xizang, Yunnan), INDIA (Darjeeling, Himachal Pradesh, Jharkhand, Jammu and Kashmir, Manipur, Meghalaya, Sikkim, Nagaland, Uttarakhand, West Bengal), MALAYSIA (Peninsular), MYANMAR, PAKISTAN, VIETNAM.

#### Chromosome number.

2n=24 (Loyal 1976; Loyal and Kumar 1984).

Selected specimens examined:

Nepal: “Ghunre, alt. 2400 m, 9 Jul 1972, *A. Maire* AMA *9*” (E00670585); “9 Jul 1972, *A. Maire*, AMA *8*” (E00754794).

**W Nepal: MUGU**: “Dalupata, Carpinus faginea forest, aspect N 40°W, alt. 2220 m, Incination 35° (S8301), 1 Oct 1983, *H. Tabata* et al. *20718*” (KYO, photo); “Between Toli and Rara, *Aesculus indica* forest along Khatyar Khola river, alt. 2400 m, 8 Sep 1983, *H. Tabata* et al. *24936*” (KYO, photo).

**C Nepal KATHMANDU**: “Chandragiri, near Thankot, Kathmandu, Nuwakot, c. 2000 m, 19 Nov 1988, *T. Nakaike 3855*” (PE 01622152), “l.c. *3856*” (PE 01593958), “l.c. *3860*” (PE 01634004); “Siwapuri, Kathmandu, Nuwakot, alt. 2500 m, 23 Nov 1988, *T. Nakaike 3877*” (PE 01593956); “Between Siwapuri and Burhanilkanth, Kathmandu, alt. 2000–2550 m, 24 Nov 1988, *T. Nakaike 3890*” (PE 01593968); “Gokarna Ban, Kathmandu, alt. 1350 m, 29 Oct 1988, *T. Nakaike 3551*” (PE 0162223).

**NUWAKOT**: “Nuwakot: Tare Pati-Gul Bhanjyang, alt. c. 2100–3000 m, 25 Oct 1979, *T. Nakaike 324*” (PE).

**DOLAKHA**: “Jarsa-jiri above Sikri, Bagmati, alt. 8000 ft, 21 Sep 1968, *Banerjee*, *S. Shrestha 2850*” (US, photo); “En route from Thore Pati, alt. 3560 m to Kutumsang, alt. 2500 m and Bhanjang, alt. 2150 m, alt. 2400 m, 9 Jun 1983, *H. Tabata* et al. *18475*” (KYO, photo); “Rolwaling Khola, Simigaon (1950m)–Sekpa (2300m)–Kyalche (2700m), alt. 1950–2700 m, 31 Aug 1983, *H. Ohba* et al. *8331658*” (TI, photo; KYO, photo); “near Manga decorah, alt. 7500 ft, 13 Sep 1964, *M.L. Banerjee*, *T.B. Shrestha*, *A.V. Upadhyaya 2739*” (US, photo); “Khare Khola, Phedi Kharka (2100m)–Koplang (2100m)–Khanigaon (1700m), 14 Sep 1983, *M. Wakabayashi*, *M. Suzuki*, *A. Akiyama 8351514* [*862275*]” (KYO, photo); “Near Jiri, Dolakha, c. 1800 m, 25 Oct 1988, *T. Nakaike 3546*” (PE 01634006); Jiri, Dolakha, alt. 2000–2500 m, 3 Oct 1988, *T. Nakaike 3003*” (PE 01593980); “Between Sivalaya and Jiri, Dolakha, alt. 1800–2000 m, 24 Oct 1988, *T. Nakaike 3527*” (PE 01634003); “Between Jiri and Sivalaya, alt. 1800–2000 m, 5 Oct 1988, *T. Nakaike 3089*” (PE 01593984); “Bhote Kosi, vers Simigaon, alt. 1450 m, 14 Sep 1954, *A. Zimmernann 1295*” (KYO, photo).

**SYANGJA**: “En route from Kare to Chandrakot, alt. 1350–1400 m, 22 Sep 1976, *Y. Suehiro 322*” (KYO, photo).

**SINDHUPALCHOK**: “above Golu, alt. 2588 m. 27°54'23"N, 85°49'39"E, 11 Sep 2011, *M.F. Watson* et al. EKSIN *74*” (E00576125).

**RASUWA**: “Between Dhunche and Bharku, c. 2000 m, 29 Aug 1986, *T. Nakaike 1156*” (PE 01634001); “l.c. *1157*” (PE 01634002); “Between Lama Hotel and Sharpugaon, c. 2600–2800 m, 3 Sep 1986, *T. Nakaike 1334*” (PE 01593983), “l.c. *1333*” (PE 01593977), “l.c. *1274*” (PE 01593976)”; “Near Shabru, c. 2400 m, 6 Sep 1986, *T. Nakaike 1387*” (PE 01593998); “Between Bharku and Syabru, c. 2000–2400 m, 29 Aug 1986, *T. Nakaike 1178*” (PE 01593961).

**KASKI**: “en route from Kare to Chandrakot, alt. 1350–1400 m, 22 Sep 1976, *Y. Suehiro 32* (*III-1/1*)” (PE); “Between Potana and Dhumpus, on the stonehedge, alt. 1850 m, 3 Aug 1983, *H. Tabata* et al. *19164*” (KYO, photo).

**KATHMANDU**: “Chandragiri, alt. 1600–2000 m, 9 Oct 1986, *T. Nak*aike *2474*” (PE 01593957); “Jarkini, 1600–1700 m, 29 Sep 1986, *T. Nakaike 2005*” (PE 01593964); “Tare Bhir, alt. 1400–1900 m, 4 Oct 1979, *T. Nakaike 56*” (PE 01593970); “Bhangeri, alt. 1800–2100 m, 2 Oct 1986, *T. Nakaike 2284*” (PE 01593972); “Sankhu, alt. 1400 m, 24 Aug 1986, *T. Nakaike 1058*” (PE 01593962); “Tare Bhir, alt. c. 1400–1900 m, 4 Oct 1979, *T. Nakaike 105*” (PE 01593965); “Tare Bhir, alt. 1500–2100 m, 30 Sep 1986, *T. Nakaike 2067*” (PE 01593966); “Mulkharka, alt. c. 1700 m, 2 Oct 1986, *T. Nakaike 2329*” (PE 01634008);

**LALITPUR**: “Mt. Phulcoki, alt. 1800–2600 m, 17 Sep 1986, *T. Nakaike 1556*” (PE 01634005); “Bajrajogini, alt. 1600 m, 2 Oct 1986, *T. Nakaike 2194*” (PE 01593963); “Phulchoki, south of Kathmandu, on rather dry: ground in light shade, 1500 m, 15 Jun 1972, *H. Hara* et al. *852274*” (TI photo; KYO; photo).

**DHADING**: “Jamachok, alt. 1500–1800 m, 11 Oct 1986, *T. Nakaike 2518*” (PE 01593979); “Jamachok, alt. 1500 m, 1 Oct 1986, *T. Nakaike 2150*” (PE 01593971); “Kakani, alt. c. 2000 m, 29 Sep 1986, *T. Nakaike 1975*” (PE 01593991); “l.c. *T. Nakaike 1972*” (PE 01709395).

**BHAKTAPUR**: “Nagarkot, alt. c. 1800 m, 16 Sep 1986, *T. Nakaike 1533*” (PE 01593992).

**MAKAWANPUR**: “Daman (between Naubise and Hetauda), c. 2400 m. 23 Sep 1986. *T. Nakaike 1861*” (PE 01593973).

**RAMECHAP**: “Between Bhandar and Kenja, alt. 1700–2100 m. 7 Oct 1988. *T. Nakaike* 3203 (PE 01593974), “l.c. *3168*” (PE 01593999), “l.c. *3207*” (PE 01593975); “Bhandar (2300m)–Deorali (2700m)–Khasrubus (2400m)–Shivalaya (1800m), 27°34'N, 86°20'E–27°36'N, 86°17'E, 6 Aug 1985, *H. Ohba* et al. *8580836*” (TI, photo); “Between Sivalaya and Bhandar, alt. 1800–2500 m, 6 Oct 1988, *T. Nakaike 3125*” (PE 01594000).

**E Nepal: DHANKUTA**: “Dhankuta-Hilay-Murhay-Sinduwa, 22 Oct 1963, *M. Togashi*, *T. Tuyama s. n.*” (TI, photo).

**SANKHUWASABHA**: “Khandbari (1150m)–Mani Bhanjyang (1150m)–Sekaha (1450m)–Botebus (1800m), alt. 1150–1800 m, 1954, *H. Ohashi*, *H. Kanai*” (KYO, photo); “Papung-Bir Gaon, along path in light shade, alt. 1600–2000 m, 30 Jun 1972, *H. Kanai* et al. *7253393*” (KYO, photo); “Rive gauche de la Sun Kosi, en montaut a Chyaubaz, 1850 m, 7 Sep 1954, *A. Zimmermann* 1082a” (KYO, photo; PE); “Above Shinbun-Hatia Gola, alt. 1600–2100 m, 3 Aug 1977, *H. Ohashi* et al. *771973*” (TI, photo).

**DHANKUTA**: “Dhankuta 1300 m – Nigale 1600 m, 4 Jun 1972, *K. Kanai* et al. *725057* [*872266*, *872271*]” (KYO, photo); “Sinduwa, alt. 1100 m, 24 Oct 1963, *H. Hara* et al.” (KYO, photo); “Sinduwa, 27°04'N, 87°23'E, alt. 2400 m, 1 Aug 1973, *J.F. Dobremez* DBR NEP *1763*” (E00670592), “l.c. *1750*” (E00670593).

**ILAM**: “Near Ilam, Ilam, alt. 1500–2000 m, 5 Nov 1988, *T. Nakaike 3671*” (PE); “Mai Pokhari, 27°00'N, 87°57'E, alt. 2000 m, *J.F. Dobremez* DBR NEP *1227*” (E00754786); “l.c. *1229*” (E00670679, E00764780); “Partia Darjeling: Phalut 3600 m – Ratho Chu 2100 m – Ramam 2400 m, along path in dense forest, c. 2100 m, 4 Aug 1972, *K. Kanai* et al. *725717*” (KYO, photo).

**TAPLEJUNG**: “Ghatte-Khebang, 19 Nov 1963, *H. Hara*, *H. Kanai*, *S. Kurosawa*, *G. Murata*, *M. Togashi*, *T. Tuyama*” (KYO, photo); “Shewaden (2600 m)–Mewa Khola (2100 m)–Papung (2000 m), along path in light shade, alt. c. 2200 m, *H. Kanai* et al. *725350–C*” (KYO, photo); “Taplejung, 27°21'N, 87°41'E, alt. 2000 m, 06 Oct 1971, *J.F. Dobremez* DBR NEP *1344*” (E00670571, E00754795).

**TEHRATHUM**: “Dor 2600 m – Tute 2300 m, Jun 1972. *H. Kanai* et al. *725494*” (KYO, photo); “Chittre, alt. 2200 m. 27°06'N, 87°25'E, 16 Aug 1972, *J.F. Dobremez* DBR NEP *1495*” (E00670575), “l.c. *1507*” (E00670576), “l.c. *1484*” (E00670577).

**SOLUKHUMBU**: “Between Basa and Junbesi, Solukhumbu, alt. 2600–3500 m, 16 Oct 1988, *T. Nakaike 3356*” (PE 01593989); “Between Goem and Junbesi, Solukhumbu, alt. 3200–2650 m, 9 Oct 1988, *T. Nakaike 3243*” (PE 016344007); “De Namche Bazar en direction de la Dudh Khosi (Monjo), alt. 2900 m, 17 Oct 1954, *A. Zimmermann 1735*” (KYO, photo).

**OKHALDHUNG**A: “Tarki a Okhaldunga, alt. 2000 m, 2 Nov 1954, *A. Zimmermann 1991*” (KYO, photo).

### Selaginella ciliaris

Taxon classificationPlantaeSelaginellalesSelaginellaceae

(Retz.) Spring

E267EFE0-1F59-57F3-86B0-03591CCB2D78

**Figure 6.**
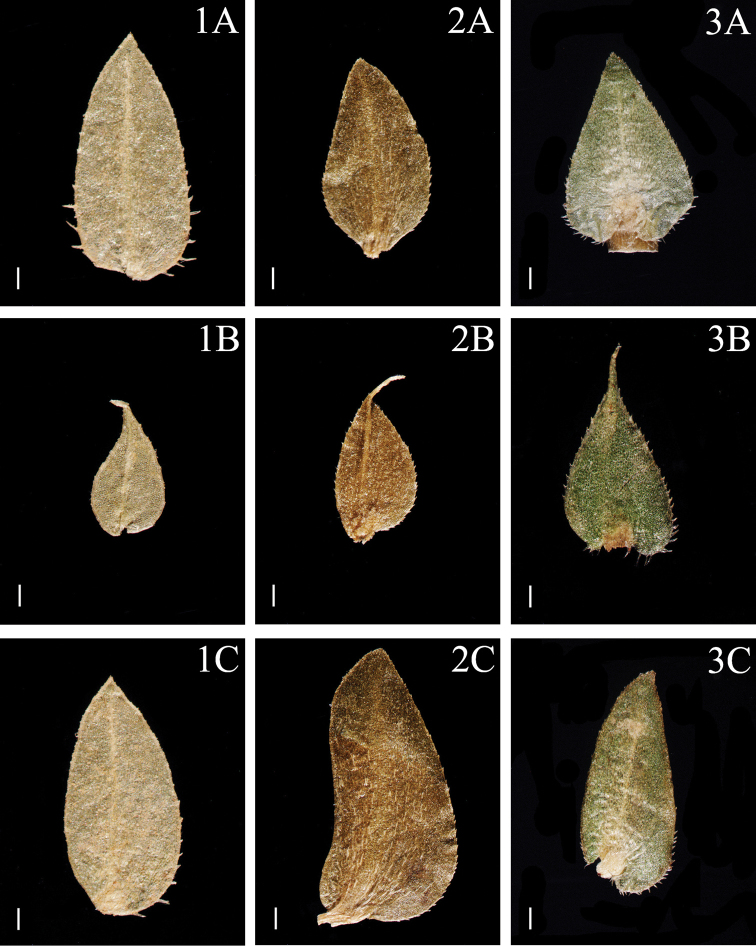
Morphological diversity of the leaves of Nepalese *Sealginella* species **1A–C***S. ciliaris* (s.n. *1225*, PE) **2A–C***S. monospora* (*Tabata* et al. *11051*, PE) **3A–C***S. trichophylla* (*Lu* & *Zhang 27625–B*, PE). A – Axillary leaves, B – Dorsal leaves, C – Ventral leaves. Scale bars: 0.2 mm.

Figs 6 (1A–C), 10E , 27

Selaginella ciliaris (Retz.) Spring, Bull. Acad. Roy. Sci. Bruxelles 10(1): 231, no. 136. 1843; Alston 1945; Panigrahi and Dixit 1968; Iwatsuki 1975; Iwatsuki 1988; Dixit 1992; Thapa 2002; Zhang 2004; Zhang et al. 2013; Fraser-Jenkins et al. 2015; Fraser-Jenkins et al. 2017.
≡ Lycopodium ciliare Retz., Observ. Bot. 5: 32. 1789.
≡ Lycopodioides ciliaris (Retz.) Kuntze, Revis. Gen. Pl. 2: 826. 1891, as “ciliare”. **Type.** SRI LANKA. E. Ceylon, *König* s.n. (holotype LD [1119541]; isotype: K).
= Lycopodium depressum Sw., Schrader. J. Bot. 1800(2): 119. 1801.
= Lycopodium belangeri Bory, Belang. Voy. Bot. 2: 12, t. 1, f. 2. 1833.
≡ Selaginella belangeri (Bory) Spring, Monogr. Lycop. 2: 242. no. 180. 1850.
= Selaginella exigua Spring, Monogr. Lycop. 2: 238. no. 175. 1850.
≡ Lycopodioides exigua (Spring) Kuntze, Revis. Gen. Pl. 2: 826. 1891. **Type.** MYANMAR. Peninsula indo-chinensi, Mergui, *W. Griffith*, *266* (H. Hooker) (holotype K [001067469]).

#### Description.

Stems 2–5(–8) cm, short-creeping, fertile stem often erect. Rhizophores restricted to lower branches or to middle of main stem. Main stems branched from throughout, branches simple to compound from base of stem, 0.3–0.4 mm in diam. in lower part. Stems terete, sulcate or not sulcate, primary leafy branches 3 or 4 pairs, simple or forked or once pinnately branched. Axillary leaves ovate-obtuse or ovate, 1.2–2 × 0.7–1.1 mm, base exauriculate, margin ciliolate in basal half, upward denticulate, apex slightly acute. Ventral leaves ovate or ovate-lanceolate, 1.4–2 × 1.4–2 mm, in base obtuse, acroscopic base enlarged, broader, margin ciliolate, subentire or minutely denticulate to apex, apex acute. Dorsal leaves ovate, 1.1–1.6 × 0.5–1 mm, slightly carinate, base subcordate or obtuse, margin minutely denticulate, apex acuminate or aristate. Strobili solitary, terminal, compact, 4.5–13 × 2–4.5 mm. Sporophylls dimorphic, ventral sporophylls ovate-triangular, margin ciliolate; dorsal sporophylls ovate-oblong, minutely denticulate and ciliolate. Megaspores yellowish, surface fine reticulate; microspores orange, surface less obviously verrucate.

#### Ecology.

On sandy and clay-slopes at the forest edge. Alt. 60–600 m.

#### Distribution in Nepal.

W, C, E.

Nepalese threatened status: not available data.

#### General distribution.

CHINA (Guangdong, Guangxi, Hainan, Taiwan, Yunnan), INDIA (Andaman and Nicobar Islands, Andhra Pradesh, Assam, Bihar, Chhattisgarh, Jharkhand, Karnataka, Kerala, Madhya Pradesh, Maharashtra, Manipur, Meghalaya, Odisha, Rajasthan, Sikkim, Tamil Nadu, Tripura, Uttarakhand, Uttar Pradesh, West Bengal), MYANMAR, SRI LANKA, BANGLADESH; INDONESIA (Java), PHILIPPINES, THAILAND, VIETNAM, NEW GUINEA, AUSTRALIA.

#### Chromosome number.

x=9; 2n=18 (Jermy et al. 1967).

Selected specimens examined:

**C Nepal: CHITAWAN**: “Tigori (near Bharatpur), alt. c. 180 m, 26 Sep 1986, *T. Nakaike 1910*” (PE 01622170).

### Selaginella monospora

Taxon classificationPlantaeSelaginellalesSelaginellaceae

Spring

39DCFBD8-E612-562A-BC9C-DD472C59EABD

Figs 6 (2A–C), 10F , 28

Selaginella monospora Spring, Mém. Acad. Roy. Sci. Belgique. 24: 135. 1850; Alston 1945; Panigrahi and Dixit 1968; Iwatsuki 1975; Iwatsuki 1988; Dixit 1992; Thapa 2002; Zhang 2004; Singh and Panigrahi 2005; Zhang et al. 2013; Fraser-Jenkins et al. 2015; Fraser-Jenkins et al.2017.
≡ Lycopodium monosporum (Spring) Hook., Bot. Misc. 9: 362. 1857.
≡ Selaginella plumosa var. monospora (Spring) Baker, J. Bot. 21: 145. 1883. **Type.** (lectotype, designated by Fraser-Jenkins et al. 2017) BHUTAN. Bootan [Khegumpa, N. of Dewangiri (Deotang)], *W. Griffith 391* Journal» [24.1.1838], Herbarium Hookerianum (K).
= Selaginella gorvalensis Spring, Mém. Acad. Roy. Sci. Belgique. 24: 256. 1850.
≡ Lycopodioides gorvalensis (Spring) Kuntze, Revis. Gen. Pl. 2: 826. 1891. **Type.** INDIA. Gorval: *Griffith* s.n., in error for Khasia (holotype: K).
= Selaginella microclada Baker, J. Bot. 22: 246. 1884. **Type.** INDIA. Sikkim, Chong-tong, alt. 4000 ft. 22 Jul 1862. *Dr. Anderson 1404* (holotype: K [001067485]).

#### Description.

Stems 35–85 cm, creeping. Rhizophores at intervals throughout length of main stem, borne on ventral side in axils of branches. Main stems branched throughout, pinnately branched. 1.5–2 mm in diam. in lower part. Axillary leaves ovate, narrowly ovate, or narrowly elliptic, 2–3 × 0.8–1.6 mm, base exauriculate, margin denticulate, apex acute. Ventral leaves ovate-triangular or oblong-falcate, 2.6–4.3 × 0.9–1.4 mm, basiscopic base decurrent, margin subentire or entire; acroscopic base enlarged, broader, overlapping stem and branches, margin denticulate, apex subacute. Dorsal leaves ovate-lanceolate or elliptic, 1–1.6 × 0.3– 0.7 mm, carinate or strongly carinate, base obtuse, not peltate, margin denticulate, apex acuminate or shortly aristate. Strobili solitary, terminal, compact, 3–15 × 1.9–5 mm, sporophylls isomorphic, slightly dimorphic to strongly dimorphic. Sporophylls dimorphic, ventral sporophylls ovate-lanceolate, carinate, base dilated, margin denticulate; dorsal sporophylls lanceolate, sharply carinate, margin minutely denticulate, apex acuminate. Megaspores brown, surface verrucate; microspores orange, surface verrucate.

#### Ecology.

On moss covered rocks or on damp slopes in forests, sparse in open slopes on edge of forest. Alt. 1650–3000 m.

#### Distribution in Nepal.

C, E.

Nepalese threatened status: not available data.

#### General distribution.

BHUTAN, CHINA (Guangdong, Guangxi, Guizhou, Hainan, Xizang, Yunnan), INDIA (Assam, Kerala, Manipur, Meghalaya, Tamil Nadu, Sikkim, West Bengal), MYANMAR, THAILAND, VIETNAM.

#### Chromosome number.

Not available data.

Selected specimens examined:

**E Nepal: SANKHUWASABHA**: “en route from Harelo to Chichila, *Castanopsis hystrix* forest, on the moist rock. alt. 1935 m, 2 Jun 1978, *H. Tabata* et al. *11051*” (PE); “Above Shinbun-Hatia Gola, alt. 1600–2100 m, 3 Aug 1977, *H. Ohashi* et al. *771954*” (TI, photo); “Seduwa, in shade under rocks, 7 VI 1965. *Banerjee*, *Upadhyay*, *Baskola* 3322” (US, photo); “Seduwa (Kasuwa Khola) prostrate, alt. 3000 ft, 6 May 1965, *Banerjee*, *Upadhyay*, *Baskola 3374*” (US, photo).

**SOLUKHUMBHU**: “Near Namche, alt. 8000 ft, 9 May 1965, *Banerjee* et al. *3418*” (US, photo); “Near Namche, alt. 8000 ft, 9 May 1965, *Banerjee* et al. *3420*” (US, photo).

**TAPLEJUNG**: “Khebang below Siling Tzokupa, 20 Nov 1963, *H. Hara* et al.” (TI, photo).

**ILAM**: “Densely forested, rocky stream-gully of Sudhung Khola, shortly below Sudhung khola, shortly below Sudhung, leading south below road, below Sundergaon, W. of Pashupatinagar on Ilam road, 9 Oct 2001, *C.R. Fraser-Jenkins 29549* (*FN 5494*)” (US, photo); “in Oak-forest on slopes above streams, between Chitregaon and Manebhanjyang, c. 4–5 km N.E of Pashupatinagar, on footpath to Manebhanjyang near indian border, N. E. of Ilam, 23 Oct 2001, *C.R. Fraser-Jenkins 29069* (*FN 5584*)” (US, photo).

**JHAPA**: “c. ½km below and S. of Kuttedara on road to Bhudabare, N. of Charali on road to Phikal and Ilam, NE of Birtamod and NW of Kakkarbhitta, forested Khola (stream-gully), 16 Aug 1998. *C.R. Fraser-Jenkins 26586* (*FN 2564*)” (US, photo).

### Selaginella trichophylla

Taxon classificationPlantaeSelaginellalesSelaginellaceae

K.H. Shing

B00FA4F1-76DF-5055-B827-E36B889A156C

Figs 6 (3A–C), 10G , 29

Selaginella trichophylla K.H. Shing, Acta Phytotax. Sin. 31(6): 569, pl. 2. 1993; Zhang et al. 2013.
≡ Selaginella monospora Spring subsp. trichophylla (K.H. Shing) X.C. Zhang, Fl. Reipubl. Popularis Sin. 6(3): 189. 2004. **Type.** CHINA. Yunnan, Gongshan, Dulongjiang River, alt. 1450–1500 m, in sylvis frondosis, 22 VIII 1982, *Qinghai-Xizang Exped. 9451* (holotype: PE! [00452190]).
= Selaginella monospora var. ciliolata W.M. Chu, Fl. Yunnan. 20: 719. 2006, syn. nov. **Type.** CHINA. Yunnan: Jingdong Xian, Ailao Shan, Xujiaba, alt. 2450 m., under evergreen broad-leaved forest, 22 Apr 1982, *J.J. He 13352* (holotype: PYU!; isotype: PE!).

#### Description.

Plants 20–35 cm, creeping. Rhizophores at intervals throughout length of main stem, borne on ventral side in axils of branches. Main stems pinnately branched throughout, stramineous, 1.0–1.5 mm in diam., in lower part, stem oval or terete, not sulcate, primary leafy branches 8–12 pairs, once or twice pinnately branched, branches sparse or thick, adjacent primary branches on main stem 1.5–2.5 cm apart. Axillary leaves on branches symmetrical, ovate or ovate triangular, 1.2–3.1 × 0.7–2.3 mm, base exauriculate or slightly subcordate, margin ciliolate at base, apex acute. Dorsal leaves ovate, 1.1–2.6 × 0.6–1.2 mm, slightly carinate, base obtuse or oblique subcordate, not peltate, margin ciliolate (more densely ciliolate at base), apex aristate. Ventral leaves ovate-triangular, 1.5–3.6 × 0.9–2.1 mm, margin denticulate; basiscopic base in base with few cilia or entire, acroscopic base enlarged, broader, overlapping stem and branches, margin ciliolate, in upper part denticulate, apex acute. Strobili solitary, terminal, compact, dorsiventrally complanate, 4.0–6.5 × 1.2–2.6 mm, sporophylls dimorphic, resupinate, not white-margined; dorsal sporophylls ovate-lanceolate, carinate, margin denticulate, apex acuminate; ventral sporophylls ovate-lanceolate, carinate, margin denticulate. Megaspores whitish surface verrucate or papillate; microspores orange, surface verrucate.

#### Ecology.

On moist cliffs, in evergreen broad-leaved forest. Alt. 1500–1600(2200) m.

#### Distribution in Nepal.

C, E, rare, requiring additional research.

Nepalese threatened status: Data-deficient (DD) according to the IUCN (2001) criteria.

#### General distribution.

BHUTAN (“Rukubi (2600) – Chendebi (2300) – Charikhachor (2250) – Neylong (2200), 14 Apr 1967, *H. Hara*, *H. Kanai*, *G. Murata*, *H. Ohashi*, *O. Tanaka & T. Yamazaki 4105*” (KYO); “Yuto La, between Bumthang and Trongsa, 8500 ft., Shady banks in deciduous forest. *F. Ludlow*, *G. Sherriff*, *J.H. Hicks 17023*” (KYO; L.4328981)), CHINA (Yunnan, Guangxi, Guizhou, Guandong, Hainan), INDIA (Sikkim (E Sikkim District. Above and S. of Penlang Bazaar, below and on way up to Namphung Peak of the Tinjure ridge, W. of Tashi View-Point, Across valley to the north of Gangtok. Just below crest on N. side of densely mixed-forest ridge. 29 Sep 1998. *C.R. Fraser-Jenkins 27054 (FN 3031)*” (L.4328985)), VIETNAM (Cao Bang).

#### Chromosome number.

Not available data.

Selected specimens examined:

**C Nepal**: **DOLAKHA**: “Jiri, Dolakha, alt. c. 2200 m, 4 Oct 1988, *T. Nakaike 3076*” (PE).

**E Nepal**: **ILAM**: “Mai Majuwa-Dhara Pani, alt. 1500–1600 m, 4 Dec 1963, *H. Hara* et al.” (KUN; L.3498103).

#### Note.

We examined the type and general collections in herbaria PE, KUN and PYU for *S. trichophylla* and S. monospora var. ciliolata: the taxon described as S. monospora var. ciliolata W.M. Chu (in Chu 2006) and listed in the “Uncertain taxa” in Flora of China (Zhang et al. 2013: 66) has many similarities in morphological features with *S. trichophylla*. The only distinct feature is the spinules on the upper side of the leaves, but these are not always present and it is likely that this trait is associated with a more humid habitat.

### Selaginella repanda

Taxon classificationPlantaeSelaginellalesSelaginellaceae

(Desv. ex Poir.) Spring

CA00E9FA-8296-5BBC-B413-96273E21559A

**Figure 7.**
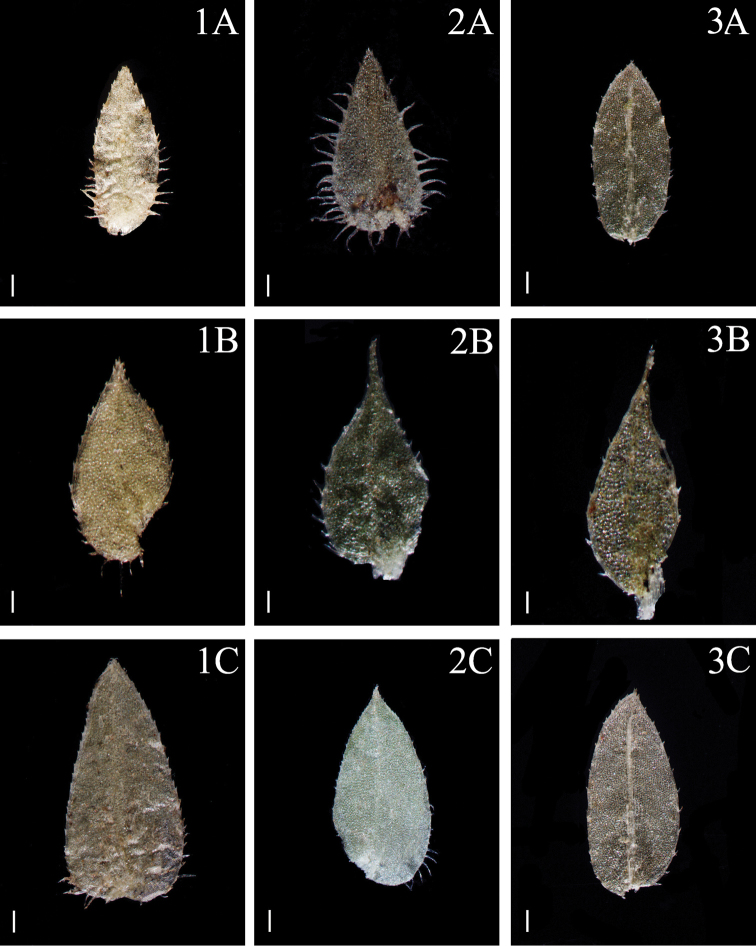
Morphological diversity of the leaves of Nepalese *Sealginella* species **1A–C***S. repanda* (*Nakaike 3708*, PE) **2A–C***S. vaginata* (*Nakaike 1102*, PE) **3A–C***S. chrysorrhizos* (*Nakaike 3708*, PE). A – Axillary leaves, B – Dorsal leaves, C – Ventral leaves. Scale bars: 0.2 mm.

Figs 7 (1A–C), 10H , 30

Selaginella repanda (Desv. ex Poir.) Spring, in Gaudich., Voy. Bonité, Bot. 3: 329. 1846; Iwatsuki 1975; Iwatsuki 1988; Dixit 1992; Thapa 2002; Zhang 2004; Zhang et al. 2013; Fraser-Jenkins et al. 2015; Fraser-Jenkins et al. 2017.
≡ Lycopodium repandum Desv. ex Poir., Encycl., Suppl. 3(2): 558. 1814. **Type.** PHILIPPINE. In insulis Philippinis. Desvaux s.n. (holotype: P).
= Lycopodium barbatum Kaulf., Enum. Filic.: 18. 1824.
≡ Selaginella barbata (Kaulf.) Spring, Bull. Acad. Roy. Sci. Bruxelles 10(1): 226. 1843. **Type.** PHILIPPINE. Isl. Manila. Chamisso s.n. (?).
= Lycopodium tetragonostachyum Wall. ex Hook. & Grev., Bot. Misc. 2: 389. no. 129. 1831.
≡ Selaginella tetragonostachya (Wall. ex Hook. & Grev.) Spring, Bull. Acad. Brux. 10: 234, no. 163. 1832. **Type.** BURMA. Mts. of Ava, *Wallich p. p.* (?).
= Lycopodium tetragonostachyum var. major Grev. & Hook., Bot. Misc. 2: 389. 1832. **Type.** INDIA. Rajemah Mts, of Hindustan. *Dr. Wallich*; Mongher, Dr. Hamilton; Hilly country of Madras. Dr. Wight = S. radicata (syntypes: E).
= Selaginella implexa J. Scott, J. Agri-Hort. Soc. Ind., N. S., 1(2): 262. 1868. **Type.** INDIA. Parasnath, 2000 ft., Bheerboom and Hills near Balasore (holotype C).
= Selaginella suberecta Baker, J. Bot. 22: 245, no. 146. 1884. **Type.** MALAYSIA. Malacca, *Griffith* s.n. (?).

#### Description.

Stems 5–30 cm, suberect to erect. Rhizophores borne from base to upper part of main stem or restricted to creeping rhizomes and stolons, on ventral side in axils of branches. Main stem branches above base, without branching part up to 15 cm, stems oval or terete. Axillary leaves more or less similar to lateral leaves, ovate or ovate-lanceolate, 2–3 × 1–1.4 mm, base exauriculate, margin ciliolate, in upper part subdentate, apex obtuse. Ventral leaves spreading, ovate, 2.2–3 × 1–1.5 mm, sub-falcate, rounded at base, basiscopic base with few cilia, acroscopic base rounded, not overlapping stem and branches, margin ciliolate in basal half, in middle and upper part dentate to denticulate, apex acute. Dorsal leaves ovate, imbricate, base cordate, in base margin ciliolate, margin in middle and upper part denticulate, apex acute to acuminate. Strobili tetragonous, submonomorphic, 3–8 × 1.5–3 mm. Sporophylls uniform, submonomorphic or sometimes dorsal sporophylls longer, ovate, margin ciliolate, apex acuminate. Megaspores yellowish orange, baculate, surface regulate or reticulate; microspores orange, surface irregular elevations.

#### Ecology.

In the open or semi-shaded places on rocks or under shrubs on soil banks. Alt. 200–400 m.

#### Distribution in Nepal.

C, E.

Nepalese threatened status: VU (Fraser-Jenkins et al. 2015).

#### General distribution.

CAMBODIA, CHINA (Guangxi, Guizhou, Hainan, Taiwan, Yunnan), INDIA (Andhra Pradesh, Assam State, Bihar, Chhattisgarh, Jharkhand, Karnataka, Madhya Pradesh, Meghalaya, Maharashtra, Mizoram, Nagaland, Odisha, Rajasthan, Sikkim, Tamil Nadu, Uttarakhand, Uttar Pradesh, West Bengal), INDONESIA, LAOS, MALAYSIA, MYANMAR, PHILLIPINES, THAILAND, VIETNAM.

#### Chromosome number.

Not available data.

Selected specimens examined:

**C Nepal: MAKAWANPUR**: “Suntari, W. of Hetauda, Makawanpur, alt. c. 200 m, 8 Nov 1988, *T. Nakaike 3719*” (PE); “l.c. *T. Nakaike 3708*” (PE); “l.c. *T. Nakaike 3703*” (PE); “l.c. *T. Nakaike 3715*” (PE).

### Selaginella vaginata

Taxon classificationPlantaeSelaginellalesSelaginellaceae

Spring

A61D46E7-4B49-5588-9E43-BC8D00C116F2

Figs 7 (2A–C), 10I , 31

Selaginella vaginata Spring, Mém. Acad. Roy. Sci. Belgique 24: 87. 1850; Iwatsuki 1975; Iwatsuki 1988; Dixit 1992; Thapa 2002; Zhang 2004; Zhang et al. 2013; Fraser-Jenkins et al. 2015; Fraser-Jenkins et al. 2017.
≡ Lycopodioides vaginata (Spring) Kuntze, Rev. Gen. Pl. 1: 827. 1891. **Type.** (lectotype, designated by Fraser-Jenkins et al. 2017) INDIA. NE India, Meghalaya, Khasiya (Khasia) [cited by Spring as “Gorval” i. e. Garhwal, Uttarakhand, in error], *W. Griffith* (K). Also cited as syntypes were Bhutan, “Bootan, W. Griffith” (K); and South India, Tamil Nadu, “Nelligheries [Nilgiris], *G.S. Perottet 642* (P [= S. radicata]).
= Selaginella thomsonii Hieron, Hedwigia 43: 38. 1904. **Type.** INDIA. India orientalis: habitat in montibus Khasia, alt. s.m. 4–6000 feet, regione temperate, *J.D. Hooker* et *T. Thomson* (holotype: K?; isotype: B [20 0176901]).

#### Description.

Stems 3.5–10 cm, creeping, fertile stems erect. Rhizophores restricted at intervals throughout length of creeping stem and branches and to lower part of erect fertile branches, borne on ventral side in axils of branches. Main stems branched throughout, 0.2–0.4 mm in diam. in lower part. Stem stramineous, terete, sulcate or not, branches few; erect fertile stems pinnately branched throughout. Axillary leaves ovate-triangular, 1.2–2.5 × 0.5–1.5 mm, base exauriculate, margin ciliolate in basal part, subentire in middle and upper part. Ventral leaves ovate-lanceolate or oblong-falcate, 1.6–3.2 × 0.8–1.5 mm, basiscopic base rounded, margin denticulate in basal half, denticulate upward; acroscopic base endlanged, broadly overlapping stem and branches, margin ciliolate, sparsely long ciliolate at base, apex acute. Dorsal leaves ovate-lanceolate, 0.8–2.3 × 0.4–1.1 mm, imbricate, base subcordate, cuneate, or obtuse, not peltate, margin long ciliolate at base, shortly ciliolate (rarely long ciliolate) upward, apex acuminate or aristate. Strobili solitary or in pairs, terminal, 10–15(–40) × 2–3.5 mm. Sporophylls dimorphic or slightly dimorphic, dorsal sporophylls ovate-lanceolate, margin ciliolate or denticulate, apex acuminate; ventral sporophylls ovate-lanceolate, margin denticulate or ciliolate, apex acuminate. Megaspores yellowish, surface verrucate; microspores orange, surface verrucate and rugate.

#### Ecology.

Terrestrial or epilithic, forming a carpet on vertical banks and rocks evergreen or seasonally green. Alt. 500–2900 m.

#### Distribution in Nepal.

W, C, E.

Nepalese threatened status: not available data.

#### General distribution.

BANGLADESH, BHUTAN, CAMBODIA, CHINA (Beijing, Chongqing, S Gansu, Guangxi, Guizhou, Henan, Shaanxi, Sichuan, Xizang, Yunnan), INDIA (Assam State, Chhattisgarh, Himachal Pradesh, Jammu and Kashmir, Jharkhand, Madhya Pradesh, Manipur, Meghalaya, Nagaland, Odisha, Sikkim, Tripura, Uttarakhand, West Bengal), LAOS, MYANMAR, PAKISTAN, THAILAND, VIETNAM.

#### Chromosome number.

Not available data.

Selected specimens examined:

**C Nepal: KASKI**: “Mahendra Cave, Pokhara, alt. c. 700 m, 11 Nov 1988, *T. Nakaike 3829*” (PE).

**MAKAWANPUR**: “[Pisulin] Fishling, near Mugling, Gorkha, alt. c. 300 m, 12 Nov 1988, *T. Nakaike 3830*” (PE).

**PALPA/SYANGJA**: “Angahora, between Butwal and Pokhara, alt. c. 650 m, 9 Nov 1988, *T. Nakaike 3732*” (PE); “Bategora, between Butwal and Pokhara, alt. c. 700 m, 9 Nov 1988, *T. Nakaike 3736*” (PE).

**RAMECHAP**: “between Bhandar and Kenja, alt. 2100–1700 m, 7 Oct 1988, *T. Nakaike 3160*” (PE).

**KATHMANDU**: “near Tribhuwan Airport, alt. c. 1300 m, 15 Sep 1986, *T. Nakaike 1506*” (PE); “Nagarjun, alt. c. 1400 m, 26 Aug 1986, *T. Nakaike 1102*” (PE); “Swayambhunath, alt. c. 1400 m, 7 Oct 1986, *T. Nakaike 2429*” (PE).

**CHITAWAN**: “Muglin [Mugling] (between Kathmandu and Pokhara), alt. c. 280 m, 26 Sep 1986, *T. Nakaike 1929*” (PE).

**E Nepal: SOLUKHUMBU**: “near Junbesi, Solukhumbu, alt. c. 2900 m, 20 Oct 1988, *T. Nakaike 3453*” (PE); “Karodo, near Kenja, Solukumbu, alt. c. 1750 m, 22 Oct 1988, *T. Nakaike* 3505” (PE); “Karodo, near Kenja, Solukumbu, alt. c. 1750 m, 22 Oct 1988, *T. Nakaike 3495*” (PE).

**TAPLEJUNG**: “Shewaden (2600 m)–Mewa Khola (2100 m)–Papung (2000 m), along path in light shade, alt. c. 2200 m, 26 Jun 1972, *H. Kanai* et al. *725351*” (TI, photo; KYO, photo); “Ghatte-Khebang, 19 Nov 1963, *H. Hara* et al.” (TI, photo); “Bharomdin-Tharpu, 25 Nov 1963, *H. Hara* et al.” (KYO, photo); “Selap-Zongi-Walunchung Gola, 10 Nov 1963, *H. Kanai* et al. s.n.” (KYO, photo); “Ghatte-Khebang, 19 Nov 1963, *H. Hara* et al.”(KYO, photo).

**SANKHUWASABHA**: “Papung-Bir Gaon, along path in light shade, alt. 1600–2000 m, 30 Jun 1972, *H. Kanai* et al. *7253393*” (TI); “Papung (2000 m)–Bir Gaon (1600 m)–Sangrati Pati (1050 m), alt. 1300 m, 26 Aug 1977, *H. Ohashi* et al. *772767*” (TI, photo); “Papung 2000 m-Bir Gaon 1600 m, 30 Jun 1972, *H. Kanai* et al. *725393* [*873274*]” (KYO, photo).

**ILAM**: “Mai Majuwa-Mai Pokhari-Dhara Pani, 4 Dec 1963, *H. Hara* et al.” (KYO, photo); “Bilbatay Bhanjang-Tinjuray-Hati Sar, 27 Oct 1963, *H. Hara* et al. s.n.” (KYO, photo);

**BHOJPUR**: “Birgaon 1600 m-Suju Khola 1400 m-Dingla 1000 m, 1 Jul 1972, *H. Kanai* et al. *725426*” (KYO, photo); “Dingla 1000 m-Doban 800 m, on muddy rock along path in shade, 2 Jul 1972, *H. Kanai* et al. *725456*” (KYO, photo); “Birgaon-Dingla, alt. 1600–1000 m, 01 Jul 1972, *K. Ohashi* et al. *725426*” (E00670675).

**DHANKUTA**: “Teku Nala 800 m-Tamur Bridge 300 m, 9 Jul 1972, *H. Kanai* et al. *725511*” (KYO, photo); “Dhankuta, 26°50'N, 87°20'E, alt. 400 m, 11 Oct 1971, *J.F. Dobremez* DBR NEP *1370*” (E00670683).

**SUNSARI**: “Dharan, 26°49'N, 87°18'E, alt. 600 m, 13 Aug 1972, *J.F. Dobremez* DBR NEP *1442*” (E00670587).

### Selaginella chrysorrhizos

Taxon classificationPlantaeSelaginellalesSelaginellaceae

Spring

000E4F3F-4E55-5417-938A-3EA17449CDDB

Figs 7 (3A–C), 11A , 32

Selaginella chrysorrhizos Spring, Monogr. Lycop. 2: 251, no. 189. 1850, p. p.; Iwatsuki 1975; Dixit 1992; Thapa 2002; Fraser-Jenkins et al. 2015; Fraser-Jenkins et al. 2017. **Type.** (lectotype, designated by Fraser-Jenkins et al. 2017) Assam, *Griffith 141* (K).
= Selaginella panchghaniana R.D. Dixit, Bull. Bot. Surv. India 25(1–4): 226, t. 1, f. 3. 1985; Dixit 1992. **Type.** INDIA. Maharashtra-Mahabaleshwar: Panchghani, 7 Nov 1966, *Panigrahi 11739* (holotype: BSA).

#### Description.

Stems 8–12 cm, evergreen or seasonally green, suberect. Rhizophores restricted to base of stem. Main stems branched from near base or from lower part upward, in basal part main stem 0.5–1.1 mm in diam. Stems glabrous, glossy yellow, sulcate, primary leafy branches 5–8 pairs, forked or once or twice pinnately branched. Axillary leaves ovate-oblong, 1.0–2.0 × 0.5–0.8 in base slightly cuneate, margin denticulate, apex obtuse. Ventral leaves ovate-oblong, 0.6–0.8 × 1–2 mm, ascending, acroscopic base ovate-oblong, slightly dilated, imbricate, distantly rotundate at the base, margin denticulate, basiscopic base, entire, except apices, apex obtuse. Dorsal leaves ovate, 1.8–2 × 0.5–0.8 mm, subfalcate, margin denticulate, apex shortly cuspidate. Strobili solitary, terminal, compact, 3–7 × 1.5–2 mm. Sporophylls dimorphic, ventral sporophylls ovate, aristate, margin ciliate; dorsal sporophylls oblong, obtuse, margin ciliate-dentate. Megaspores dark-brown, surface verrucate; microspores pale-brown, surface verrucate.

#### Ecology.

On banks and on large stones. Alt. 200–2000 m.

#### Distribution in Nepal.

W, C, E.

Nepalese threatened status: not available data.

#### General distribution.

BANGLADESH, BHUTAN, INDIA (Assam, Kerala, Madhya Pradesh, Manipur, Meghalaya, Mizoram, Sikkim, West Bengal), LAOS, MYANMAR, THAILAND, VIETNAM.

#### Chromosome number.

Not available data.

Selected specimens examined:

**C Nepal**: KASKI: “Bhoot Bridge, between Butwal and Pokhara, alt. c. 300 m, 9 Nov 1988, *T. Nakaike 3723*” (PE).

**MAKAWANPUR**: “Pisulin [Fishling], near Muglin, Gorkha [Makawanpur District], alt. c. 300 m, 12 Nov 1988, *T. Nakaike 3831*” (PE).

**E Nepal: SOLUKHUMBHU**: “between Junbesi and Rachowa, Solukumbu, alt. 2600–3400 m, 12 Dec 1988, *T. Nakaike 3304*” (PE).

**SANKHUWASABHA**: Simbu, 27°22'N, 87°47'E, alt. 1800 m, 5 Dec 1971, *J.F. Dobremez* DBR NEP *1335*” (E00754784); “Sunaturi, W. of Hetauda, Makawanpur, alt. c. 200 m, 8 Nov 1988, *T. Nakaike 3702*” (PE); “ l.c. *3698*” (PE); “l.c. *3720*” (PE); “l.c. *3706*” (PE); “Simbu, 27°22'N, 87°47'E, alt. 2000 m, 5 Dec 1971, *J.F. Dobremez* DBR NEP *1333*” (E00670601, E00754800); “Simbu, 27°22'N, 87°47'E, alt. 1800 m, 5 Oct 1971, *J.F. Dobremez* DBR NEP *1335*” (E00670602).

### Selaginella reticulata

Taxon classificationPlantaeSelaginellalesSelaginellaceae

(Hook. & Grev.) Spring

D2F6A679-4427-55BC-8DCA-CF493CC768F6

**Figure 8.**
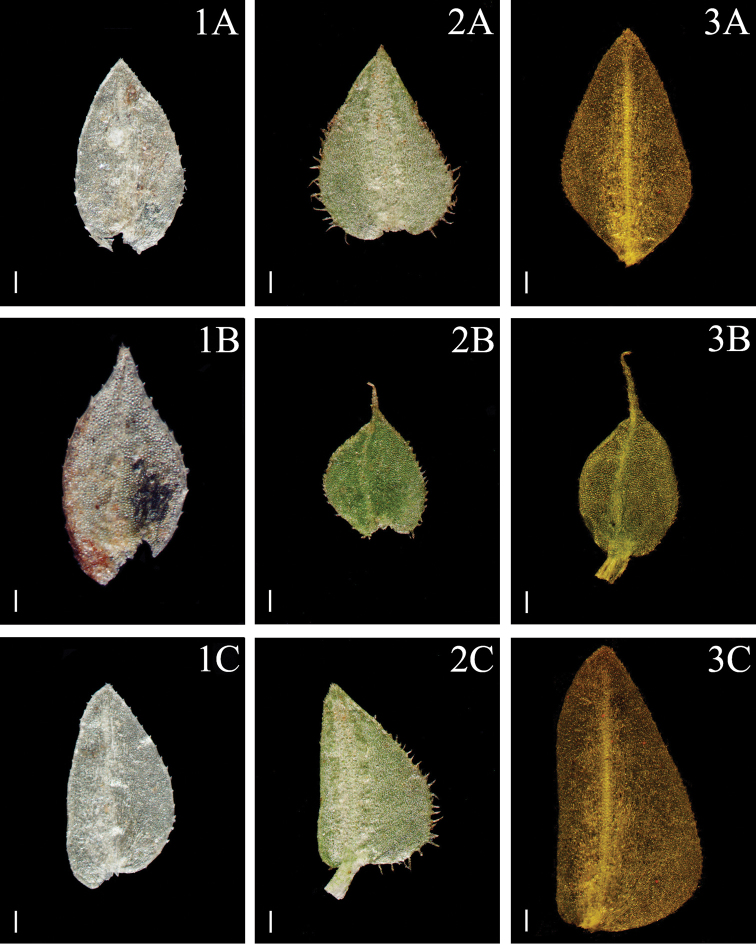
Morphological diversity of the leaves of Nepalese *Sealginella* species **1A–C***S. reticulata* (*Nakaike 1760*, PE) **2A–C***S. subdiaphana* (*Zhang 5*, PE) **3A–C***S. tenuifolia* (*PE-Xizang Exped. PE6280*, PE). A – Axillary leaves, B – Dorsal leaves, C – Ventral leaves. Scale bars: 0.2 mm.

Figs 8 (1A–C), 11B , 33

Selaginella reticulata (Hook. & Grev.) Spring, Bull. Acad. Roy. Sci. Bruxelles 10(1): 233. 1843; Alston 1945; Dixit 1992; Fraser-Jenkins et al. 2015; Fraser-Jenkins et al. 2017.
≡ Lycopodium reticulatum Hook. & Grev., Bot. Misc. 2: 402. 1831. **Type.** MYANMAR. Mt. Ava. *Dr. Wallich* s.n. (holotype: K [001067446]).
= S. nudicaulis Spring, Monogr. Lyc. II: 235. 1850.
= S. rajasthanensis Gena, Bhardwaja & A.K. Yadav, Amer. Fern J. 69(4): 119. 1979. **Type.** INDIA. Kundakhon, Shahabad, Kota, Rajasthan, growing on an isolated moist rock, Sep 1977, *C.B. Gena*, *A.K. Yadav* (holotype: PUN (PYN 2610); isotypes: Pteridophyte Biology Lab. Govt. College, Ajmer, India (N° PBL/77/S1-6/28/671); B; BM [001038102]; CAL; K [001067491]; LWF; NY; US [00134391]).
= Selaginella jainii R.D. Dixit, Bull. Bot. Surv. India 25(4-Jan): 225, f. 2 A–H. 1985. **Type.** INDIA. Madhya Pradesh, Bilaspur: Siang, 22 Feb 1972, *Panigrahi 16838 A*, *Plant number-2* (holotype: CAL); Madhya Pradesh, Bilaspur: Siang, 22 Feb 1972, *Panigrahi 16838 B* (isotype: BSA).
= Selaginella panigrahii R.D. Dixit, Bull. Bot. Surv. India 25(4-Jan): 226, f. 4. A–H. 1985; Dixit 1992. **Type.** INDIA. Madhya Pradesh, Bastar-Kutumsar: Kanger valley, 19 Fed 1963, *Panigrahi 1119* (holotype: CAL; isotype: BSA).
= S. nairii R.D. Dixit, Bull. Bot. Surv. India 26(2-Jan): 106. 1985; Dixit 1992. **Type.** INDIA. Orissa, Jeypore, c. 850 m., 17 Sep 1970, *Nair 40637*” (holotype: CAL); INDIA. Orissa, Jeypore, c. 850 m., 17 Sep 1970, *Nair 40637A* (isotype: CAL); INDIA. Orissa, Devighat, 900 m., 22 Sep 1970, *Nair 40692* (paratype: CAL).

#### Description.

Stems 6–15 cm, erect or suberect. Rhizophores in basal part or one-third creeping stem and branches, on ventral side in axils of stem branches. Main stems, much branched, slender, primary branches on intervals, 0.8–1.2 mm in diam. in lower part, second branches simple or forked. Axillary leaves, ovate, 0.9–1.3 × 0.5–0.8 mm, base rotundate, margin dentate, apex subacute. Ventral leaves ovate, 1.7–2 × 0.7–1 mm, base rotundate, basiscopic base slightly denticulate, acroscopic base rounded, not overlapping stem and branches, margin denticulate, apex subobtuse to subacute. Dorsal leaves ovate, 0.7–1 × 0.3–0.5 mm, oblique, margin thickened, distantly serrulate, apex acute to very slightly acuminate. Strobili solitary, terminal, compact, 4–7 × 1.5–3 mm. Sporophylls dimorphic, dorsal sporophylls ovate-oblong, margin ciliolate, apex acuminate; ventral sporophylls ovate, sub-pellucid, margin ciliolate, apex shortly acute. Megaspore yellow or dark-brown, surface granulose; microspores orange, surface smooth granulose.

#### Ecology.

Growing in groups on moist shaded rocks and banks at the bases of hills. Alt. 1100–3700 m.

#### Distribution in Nepal.

W, C, E.

Nepalese threatened status: not available data.

#### General distribution.

BANGLADESH, BHUTAN, INDIA (Assam State, Chhattisgarh, Jammu and Kashmir, Kerala, Madhya Pradesh, Meghalaya, Odisha, Rajasthan, Sikkim, Tamil Nadu, Uttarakhand, West Bengal), MYANMAR.

#### Chromosome number.

Not available data.

Selected specimens examined:

**C Nepal: KASKI**: “below Mahendra Pul Power-house, N part of Pokhara. Among rocks and below cliffs on both sides of river at N entrance to Seti river gorge, 5 Jan 1998, *C.R. Fraser-Jenkins*, *L.B. Tamang*, *G. Pariyar 25841* (*FN 1819*)” (US, photo).

**DOLAKHA**: “Jiri, Dolakha, alt. 2000–2500 m, 3 Oct 1988, *T. Nakaike 3064*” (PE); “Jiri, Dolakha, alt. c. 2200 m, 4 Oct 1988, *T. Nakaike 3075*” “l.c. *3076*” (PE). “near Pashupatinath, alt. c. 1340 m, 5 Oct 1979, *T. Nakaike 117*” (PE); “Pasupatinath. 21 Sep 1986, *T. Nakaike 1760*” (PE).

### Selaginella subdiaphana

Taxon classificationPlantaeSelaginellalesSelaginellaceae

(Wall. ex Hook. & Grev.) Spring

7BBC7955-D989-53A2-AC41-948819EA8FF5

Figs 8 (2A–C), 11C , 34

Selaginella subdiaphana (Wall. ex Hook. & Grev.) Spring, Bull. Acad. Roy. Soc. Brux. 10: 232. 1843; Iwatsuki 1988; Dixit 1992; Thapa 2002; Fraser-Jenkins et al. 2015; Fraser-Jenkins et al. 2017; Zhang 2018.
≡ Lycopodium subdiaphanum Wall. ex Hook. & Grev., Bot. Misc. 2: 401. 1831.
– Lycopodium subdiaphanum Wall. nom. nud. **Type.** INDIA. Montains of Sylhet and Kamoon. *Dr. Wallich n. 136* (syntypes: K; B [20 0147161–A]).
= Selaginella aggesta Spring, Monogr. Lyc. II: 89, no. 31. 1850. **Type.** INDIA. Gorval, *Griffith* (holotype: K [001067489]).
= Selaginella glauca Spring, Mém. Acad. Roy. Sci. Belgique 24(2): 252. 1850. **Type.** INDIA. Imperio Assam. Mack (H. Hooker) (holotype: K?).
= Selaginella schlagintweitii Hieron., Bot. Jahrb. Engl. 50: 2, 41, n. 17. 1913. **Type.** INDIA. Khasia, X 1855, *Schlagintweit 117* (holotype: B?).
= Selaginella namdaphaensis Sarn. Singh & Panigrahi, Ferns Fern-Allies Arunachal Pradesh 1: 64. 2005. **Type**. INDIA. Tirap District “[now Changlang District]”, Miao-Vijaynagar, 40^th^-41^st^ mile, 800 m., 30 Sep 1980, *S. Singh 74317* (holotype: CAL; isotype: ASSAM).

#### Description.

Stems 10–35 cm, creeping or suberect. Rhizophores restricted to lower one-third part of main stems, 0.8–2.1 mm in diam. in lower part. Stem slender, sulcate. Axillary leaves ovate, 1.5–2.8 × 0.5–1.8 mm, in basal part cordate, margin in basal part ciliolate, in middle and upper dentate to denticulate, apex acuminate. Ventral leaves ovate to ovate-lanceolate, 1.7–3.2 × 0.8–1.7 mm, in base slightly auriculate, basiscopic base entire, acroscopic base endlanged, broadly overlapping stem and branches, margin ciliate-dentate at base, entire towards apex, apex subobtuse. Dorsal leaves ovate, 1.2–1.6 × 0.5–0.7 mm base obtuse or slightly subcordate, margin ciliolate to denticulate, apex acute to short acuminate. Strobili solitary, terminal, compact, 4.0–8.2 × 2.0–4.0 mm. Sporophylls dimorphic, dorsal sporophylls ovate, margin denticulate, sub-acute; ventral sporophylls ovate, margin ciliolate, apex acute. Megaspores bright red, surface warty; microspore slightly-orangy red, surface warty.

#### Ecology.

On damp sheltered earth banks. Alt. 350–2500 m.

#### Distribution in Nepal.

W, C, E.

Nepalese threatened status: not available data.

#### General distribution.

BHUTAN, CHINA (Yunnan, Xizang (Naramu County)), INDIA (Assam State, Himachal Pradesh, Jammu and Kashmir, Manipur, Meghalaya, Nagaland, ?Odisha, Punjab, Sikkim, Uttarakhand, West Bengal).

#### Chromosome number.

2n=16 (Loyal 1976; Loyal and Kumar 1984).

Selected specimens examined:

**W Nepal: DANG**: “Between Kurpani and Ghorai, alt. 4000 ft, 4 Sep 1952, *O. Polunin*, *W.R. Sykes & L.H.J. Williams 1331*” (KYO, photo).

**C Nepal: RASUWA**: “Langtang: between Ramche and Betrawati, 800–1800 m, 9 Sep 1986, *T. Nakaike 1427*” (PE).

**KASKI**: “en route from Huenda to Naudanda, alt. 1100–1300 m, 21 Sep 1976, *Y. Suehiro 190*” (KYO, photo); “l.c. *Y. Suehiro 184* (*II-1*)” (PE); “Chomrong, alt. 2200 m, 26 Sep 1976, *Y. Suehiro 2298* (*Q4-I*)” (KYO, photo).

**SYANGJA**: “en route from Hyenda to Naudanda, alt. 1100–1300 m, 21 Sep 1976, *Y. Suehiro 204*” (TI, photo).

**KATHMANDU**: “Gokarna Ban, Kathmandu, alt. 1350 m, 29 Oct 1988, *T. Nakaike 3559*” (PE); “l.c. *3551*” (PE); “Kathmandu, alt. 1350 m, 3 Sep 1954, *A. Zimmermann 1005*” (KYO, photo).

**MAKAWANPUR**: “Balephi Khola, 27°50'N, 85°46'E, alt. 1000 m, 22 Aug 1971, *J.F. Dobremez* DBR NEP *829*” (E00670681).

**NUWAKOT**: “Berdawati [Betrawati], alt. 850 m, 15 Sep 1972, *A. Maire* AMA *450*” (E00670578);

**E Nepal: TAPLEJUNG**: “Shewaden (2600 m)–Mewa Khola (2100 m)–Papung (2000 m), alt. c. 2400 m, 29 Jun 1972, *H. Kanai* et al. *725350B* [*873274*]” (KYO, photo).

**SANKHUWASABHA**: “Telok, 27°22'N, 87°50'E, alt. 1200 m, *J.F. Dobremez* DBR NEP *1323*”(E00754785); “Sankhuwasabha Distr.: Khandbari (1150 m)–Mani Bhanjyang (1150 m)–Sekaha (1450 m)–Botebus (1800 m), 27 Jul 1977, *H. Ohashi* et al. *771545*” (TI, photo).

**DHANKUTA**: “Dhankuta, 26°50'N, 87°20'E, alt. 400 m, 11 Oct 1971, *J.F. Dobremez* DBR NEP *1371*” (E00670582, E00670586); “Chittre, 27°06'N, 87°25'E, alt. 2200 m, *J.F. Dobremez* DBR NEP *1483*”(E00668256).

**SUNSARI**: “Dharan-Sanguri Bhanjyang, alt. 1300 m, 2 Jun 1972, *H. Kanai* et al. *725032*” (E00670676); “Dharan, 26°49'N, 87°18'E, alt. 800 m, 04 Sep 1971, *J.F. Dobremez* DBR NEP *1779*” (E00670604); “Dharan 400 m-Sanguri Bhanjyang 1300 m, 2 Jun 1972, *H. Kanai* et al. *725032* [*872266*]” (KYO, photo).

**MORANG**: “Chisapini, alt. 500 m, 26°50'N, 87°55'E, *J.F. Dobremez* DBR NEP *1170*”(E00670677 & E00754782).

### Selaginella tenuifolia

Taxon classificationPlantaeSelaginellalesSelaginellaceae

Spring

1FEECE0F-7CD5-5FD3-B766-69BDE5C887D4

Figs 8 (3A–C), 11D , 35

Selaginella tenuifolia Spring, Mém. Bull. Acad. Roy. Sci. Belgique 24(2): 253, n. 192. 1850; Alston 1945; Iwatsuki 1975; Iwatsuki 1988; Dixit 1992; Thapa 2002; Singh and Panigrahi 2005; Fraser-Jenkins et al. 2015; Fraser-Jenkins et al. 2017; Zhang 2018. **Type.** “Mishmi Hills, *Griffith*” (syntype: K), INDIA. Khasi Hills (cited by Spring as “Gorval” in error), *Griffith* (syntype: K).
= Selaginella aureola Spring, Mém. Acad. Sci. Brux. 24: 244. no. 182. 1850; Alston 1945. **Type.** (lectotype, designated by Fraser-Jenkins et al. 2015) INDIA. Churra-Punjee, Khasya, [W.] *Griffith* (*182*), [in 1835], Herbarium Hookerianum 1867 (K).

#### Description.

Stems 10–20 cm, erect. Rhizophores restricted to lower one-third part of main stems, very slender, long, 0.9–1.3 mm in diam. in lower part, lateral branches forked a few times. Stem slender, glabrous, stramineous, pinnately branched. Axillary leaves ovate or slightly ovate-lanceolate, 2.5–3.5 × 1.5–2.0 mm, margin denticulate in basal part, apex acute. Ventral leaves ovate, 3.5–4.5 × 2.0–2.8 mm oblique, in base cordate, acroscopic base endlanged, broadly overlapping stem and branches, margin denticulate, basiscopic base rounded, margin entire, apex sub-obtuse. Dorsal leaves ovate, 1.4–2.0 × 1.0–1.5 mm, oblique, in base subrounded, margin minutely dentate, apex aristate. Strobili solitary, terminal, compact, 4–8 × 1–2.5 mm. Sporophylls dimorphic, dorsal sporophylls ovate-oblong, spreading, in basal part slightly longer than apical, margin denticulate; ventral sporophylls ovate, with round base, margin denticulate, apex aristate. Megaspores brownish, surface verrucate; microspores yellowish-brown, surface irregularly verrucate.

#### Ecology.

Terrestrial or epilithic, seasonally green, scattered in moist shady places or clayey soils in forest. Alt. 700–2200 m.

#### Distribution in Nepal.

C, E.

Nepalese threatened status: NT (Fraser-Jenkins et al. 2015).

#### General distribution.

CHINA (Xizang), INDIA (Assam State, Meghalaya, Sikkim, West Bengal), MYANMAR, THAILAND.

#### Chromosome number.

Not available data.

Selected specimens examined:

**C Nepal: DOLAKHA**: “Jiri, Dolakha, alt. 2000–2500 m, 3 Oct 1988, *T. Nakaike 3065*” (PE); “Jiri, Dolakha, alt. 2000–2500 m, 3 Oct 1988, *T. Nakaike 3061*” (PE).

**MAKAWANPUR**: “above Liot village, Basmari, c. 5 km W of Hetauda, off Narayanghat road. Densely sal-forested and rocky stream-gully on slope of first range of foothills beyond (N of) the Churiya Ghats. On rocks in forest, 24 Sep 1997, *C.R. Fraser-Jenkins* et al. *25756 (FN 1734)*” (US, photo).

**E Nepal: TAPLEJUNG**: “Bhandukay-Yamphodin-Ghatte, 16 Nov 1963, *H. Hara* et al.” (TI, photo); “Khebang-below Siling Tzokupa, 20 Nov 1963, *H. Hara* et al.” (KUN, TI photo).

#### Note.

As reported by Fraser-Jenkins et al. (2015), *S. tenuifolia* is a rather uncommon low to mid altitude species, and widespread from Himalaya to Thailand. In our study (data not published) two collections were included, one from Nepal (*T. Nakaike 3065*), and another from SW Xizang (*PE-Xizang Expedition PT6280*). Examined samples were studied on three grounds: gross morphology, morphology of spores and molecular data. Results of gross morphology did not show big differentiation in morphology features for examined samples (incl. observation of ventral and dorsal leaves, shape of leaf margin, strobili, ventral and dorsal sporophylls. Ventral leaves broadly overlapping stem and branches, margin denticulate; Dorsal leaves: ovate, at apex aristate, margin denticulate. Strobili oval in shape; ventral and dorsal sporophylls at margin denticulate.

In both examined collections megaspores on the proximal and distal surfaces are covered with irregularly sized verrucae, the main surface is vermiculate, micro-sculptures are dense spinulose. Microspores on the proximal and distal surfaces are covered with irregularly sized verrucae, micro-sculptures are echinulate.

The molecular data also support the results of morphological studies.

As a result, we consider the distribution of the species not only at low and medium altitudes but also in the highlands.

**Figure 9.**
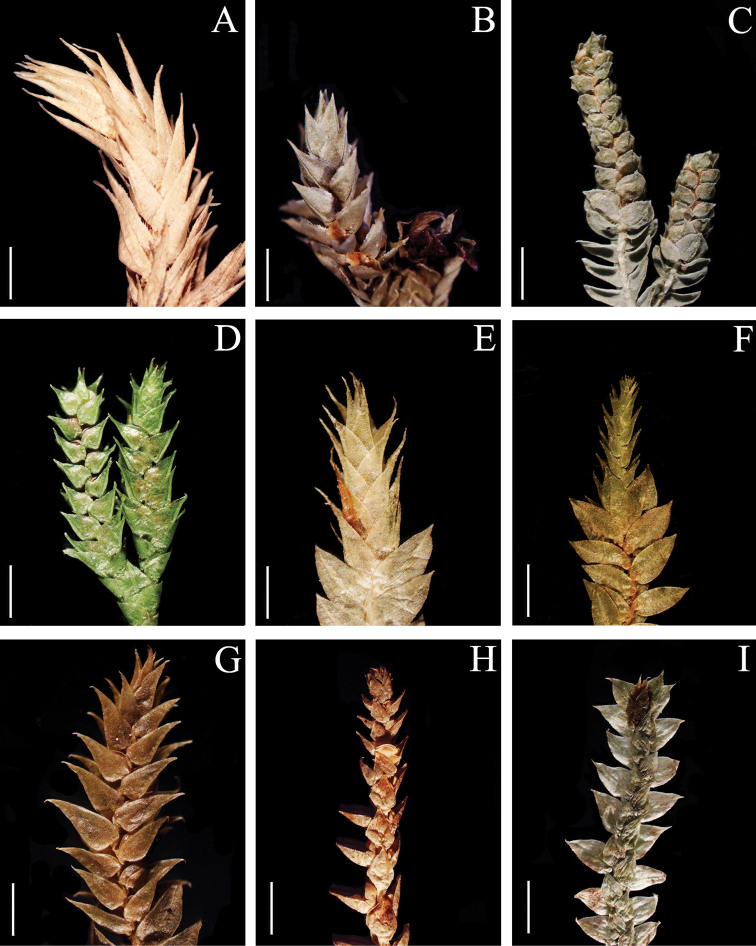
Diversity strobili of Nepalese *Selaginella* species **A***S. indica* (*Nakaike 1325*, PE) **B***S. bryopteris* (*Tabata* et al. *11989*, PE) **C***S. fulcrata* (*Nakaike 1923*, PE) **D***S. involvens* (*Zhang 345*, PE) **E***S. pallida* (*Nakaike 3740*, PE) **F***S. remotifolia* (*Nakaike 3522*, PE) **G***S. semicordata* (*Jenkins* s.n., PE) **H***S. helvetica* (*Zhang 0638*, PE) **I***S. pallidissima* (*Zhang 2746*, PE). Scale bars: 1 mm (**A–G**), 2 mm (**F, H–I**).

**Figure 10.**
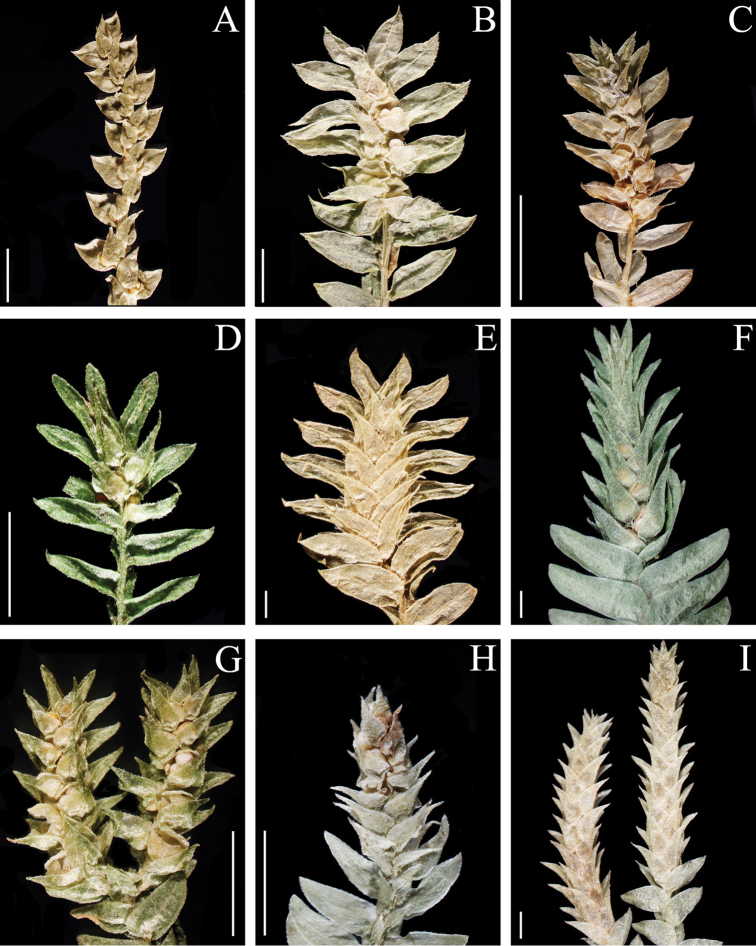
Diversity strobili of Nepalese *Selaginella* species **A***S. laxistrobila* (*Nakaike 1319*, PE) **B***S. bisulcata* (*Nakaike 3786*, PE) **C***S. pennata* (*Nakaike 3507*, PE) **D***S. chrysocaulos* (*Nakaike 1058*, PE) **E***S. ciliaris* (s.n. *1225*, PE) **F***S. monospora* (*Tabata* et al. *11051*, PE) **G***S. trichophylla* (*Lu* & *Zhang 27625-B*, PE) **H***S. repanda* (*Nakaike 3708*, PE) **I***S. vaginata* (*Nakaike 1102*, PE). Scale bars: 2 mm (**A, B**), 2 mm (**C, D, G, H**), 1 mm (**E, F, I**).

**Figure 11.**
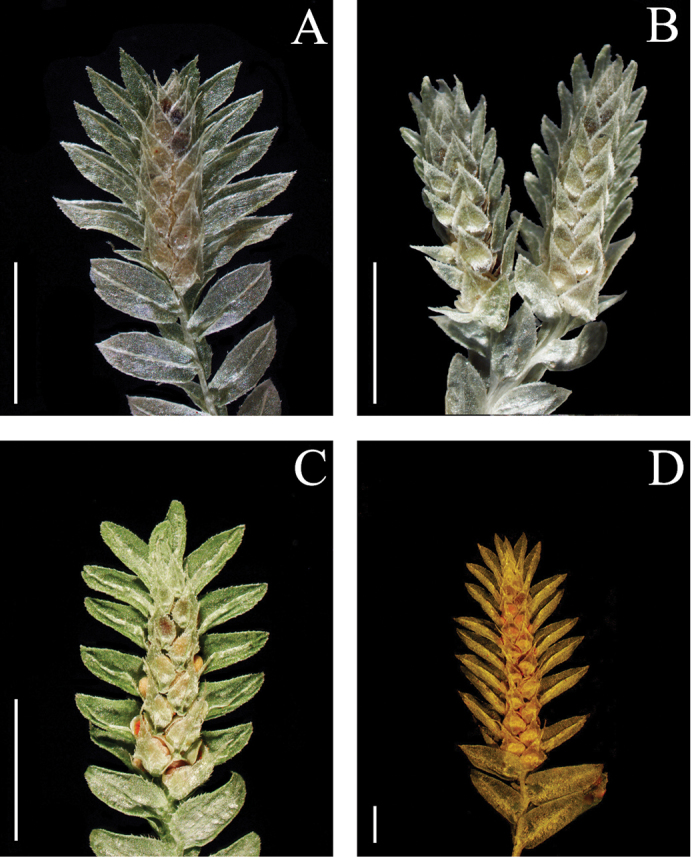
Diversity strobili of Nepalese *Selaginella* species **A***S. chrysorrhizos* (*Nakaike 3708*, PE) **B***S. reticulata* (*Nakaike 1760*, PE) **C***S. subdiaphana* (*Zhang 5*, PE) **D***S. tenuifolia* (*PE-Xizang Exped. PE6280*, PE). Scale bars: 1 mm (**A, D**), 2 mm (**B, C**).

**Figure 12.**
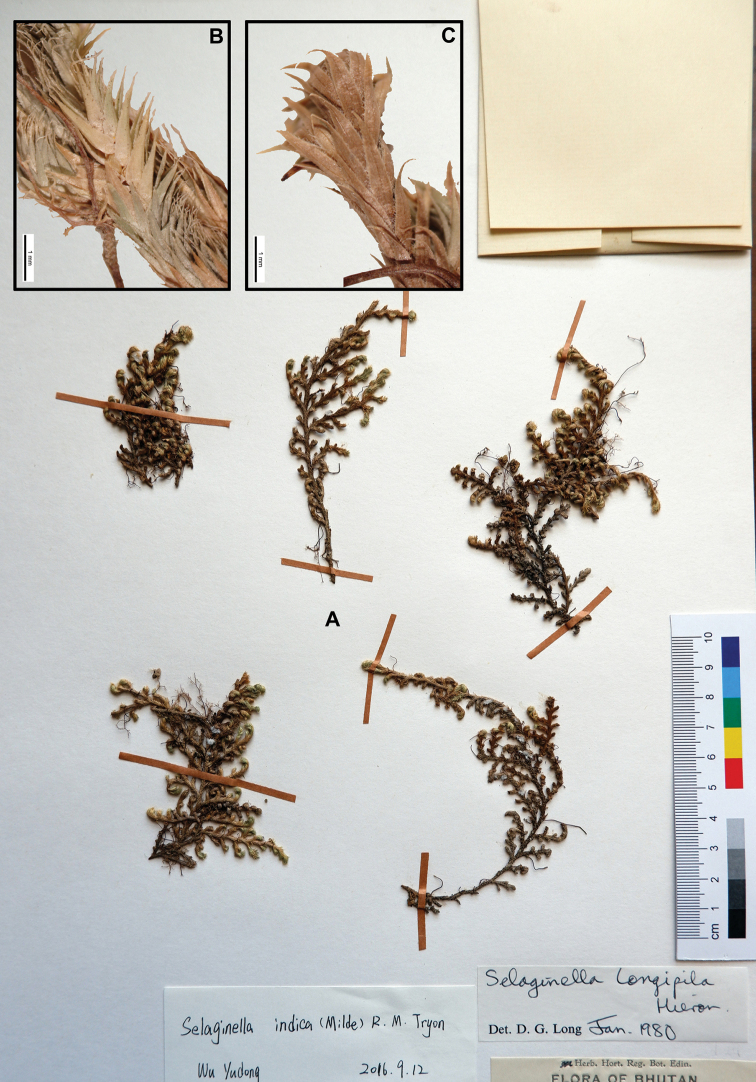
*Selaginella indica* R.M. Tryon. **A** Habit **B** lateral branches with compact tetragonal strobilus **C** lateral branches with spirally arranged monomorphic leaves (*Cooper 4866*, E).

**Figure 13.**
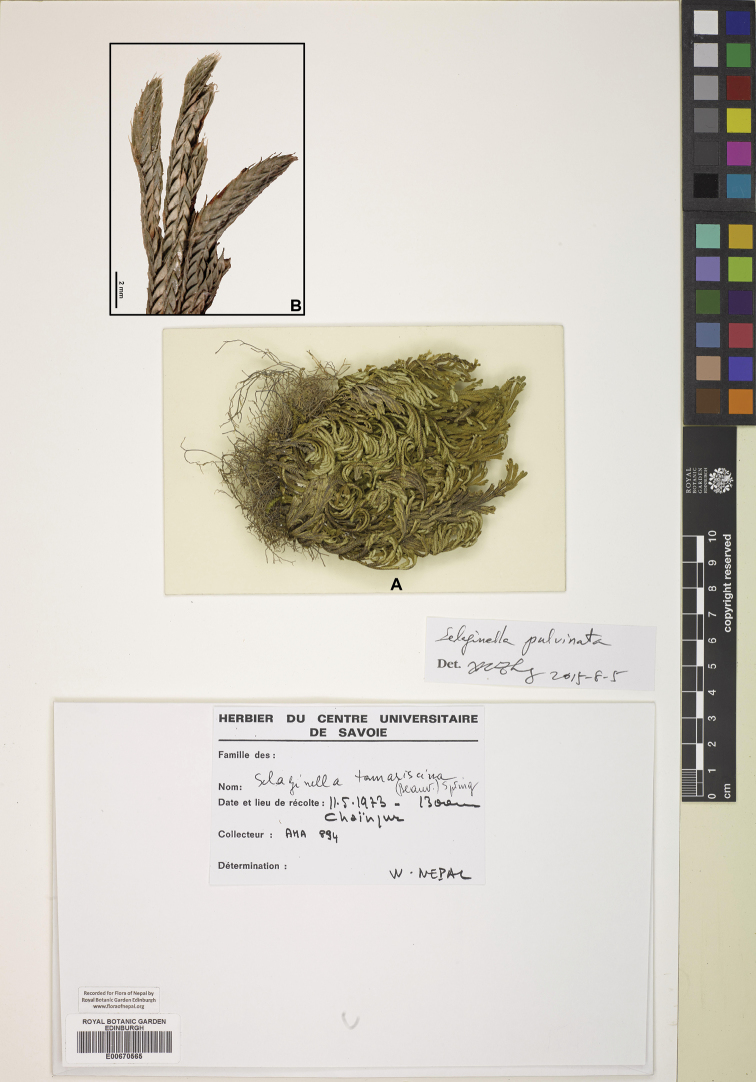
*Selaginella pulvinata* (Hook. & Grev.) Maxim. **A** Habit **B** fragment of the upper surface of the lateral branch showing dorsal leaves imbricate at branch (**A***Maire* AMA *894*, E; **B***Tabata* et al. *3520*, PE). Link: (http://data.rbge.org.uk/herb/E00670565).

**Figure 14.**
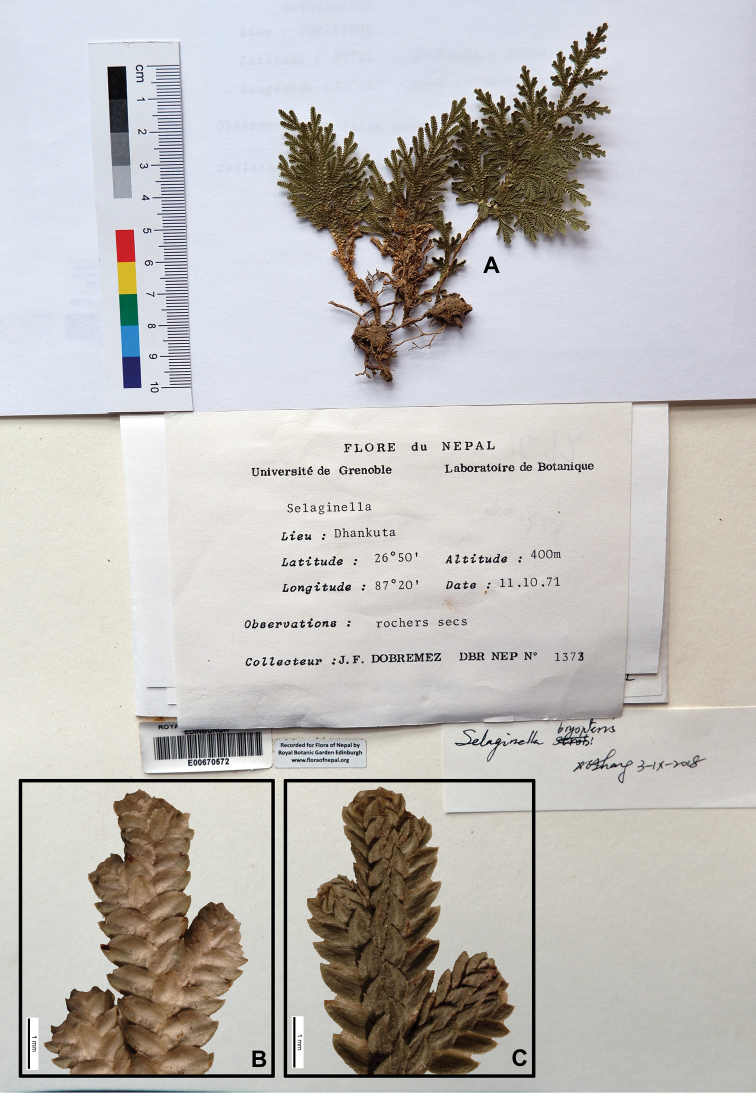
*Selaginella bryopteris* (L.) Baker. **A** Habit, upper surface **B** fragment of the lateral branches showing imbricate ventral leaves **C** fragment of the upper surface of the lateral branch showing dorsal leaves imbricate at branch (*J.F. Dobremez* DBR NEP *1373*, E). Link: (http://data.rbge.org.uk/herb/E00670572).

**Figure 15.**
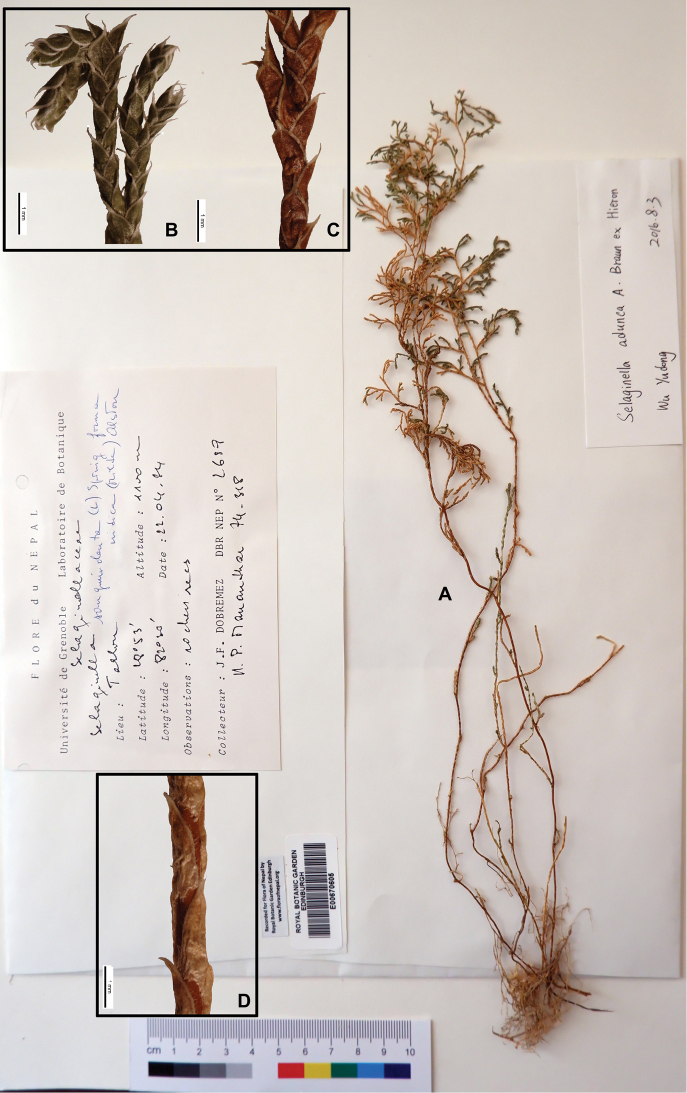
*Selaginella adunca* A. Braun ex Hieron. **A** Habit **B, C** fragment of the upper surface of the lateral branches showing imbricate at branch apices (**A**) and medial part lateral branches (**B**) **D** fragment of the main stem showing shape of leaves (*J.F. Dobremez* DBR NEP *2689*, E). Link: (http://data.rbge.org.uk/herb/E00670605).

**Figure 16.**
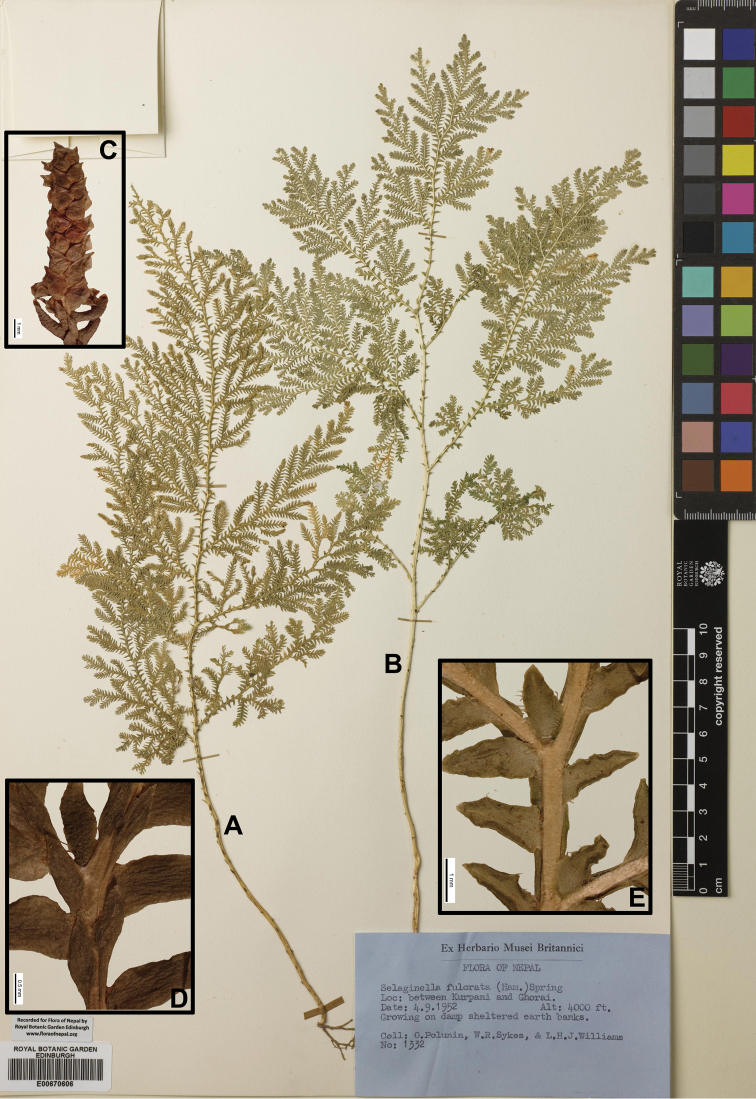
*Selaginella fulcrata* (Buch.-Ham. ex D. Don) Spring. **A** Habit, upper surface **B** habit, lower surface **C** strobilus, upper surface **D** fragment of the upper surface of the lateral branches **E** fragment of the lower surface of the lateral branches (*O. Polunin, W.R. Sykes, L.H.J. Williams 1332*, E). Link: (http://data.rbge.org.uk/herb/E00670606).

**Figure 17.**
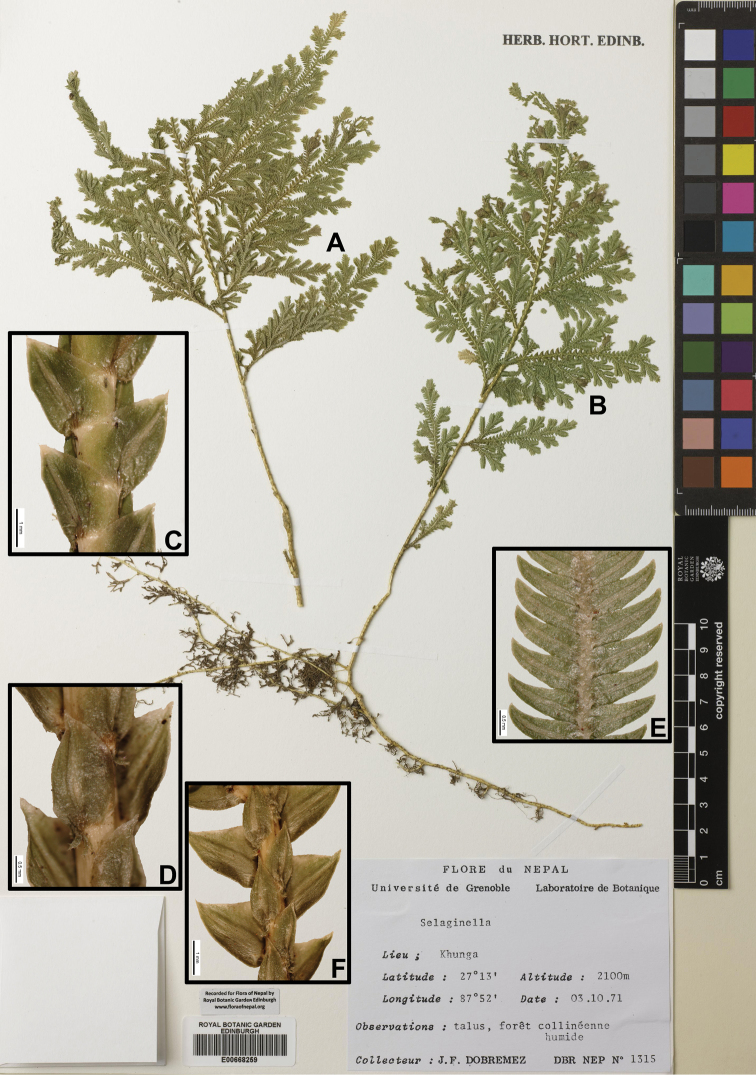
*Selaginella involvens* (Sw.) Spring. **A** Habit, upper surface of stem **B** habit, lower surface of stem **C** lower surface of the main stem **D** upper surface of the main stem **E** lower surface of the lateral branches **F** upper surface of the lateral branches (*J.F. Dobremez* DBR NEP *1315*, E). Link: (http://data.rbge.org.uk/herb/E00668259).

**Figure 18.**
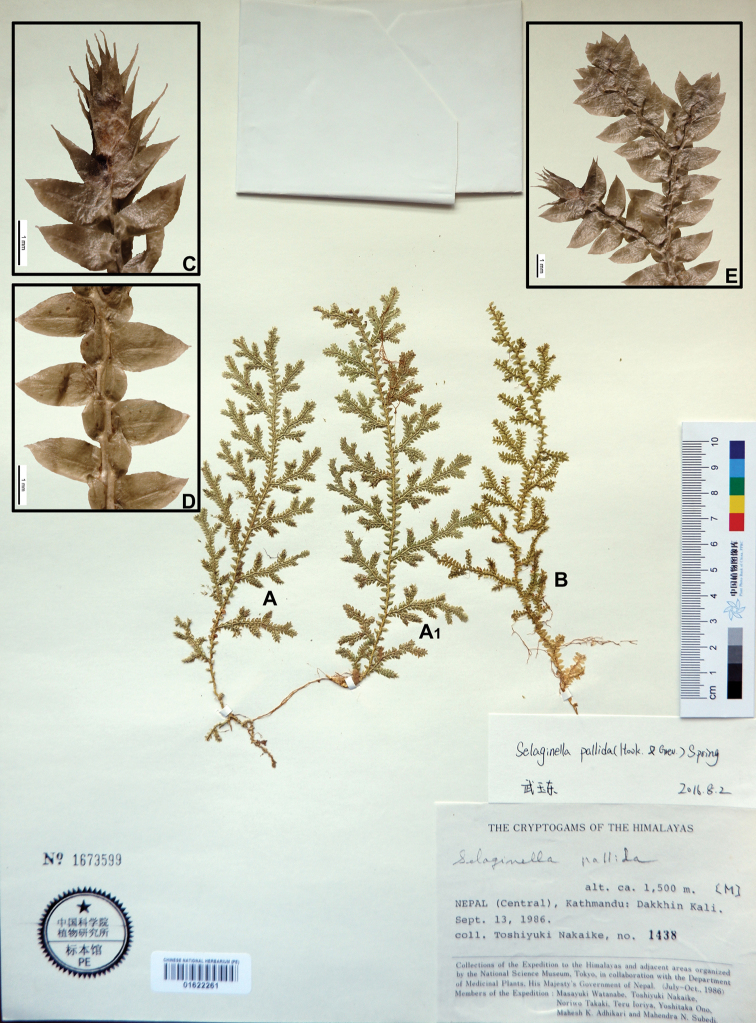
*Selaginella pallida* Spring. **A** (**A1**) Habit, lower surface **B** habit, upper surface **C** strobilus, lower surface **D** fragment of the upper surface of the lateral branches **E** fragment of the lower surface of the lateral branches (*T. Nakaike 1438*, PE).

**Figure 19.**
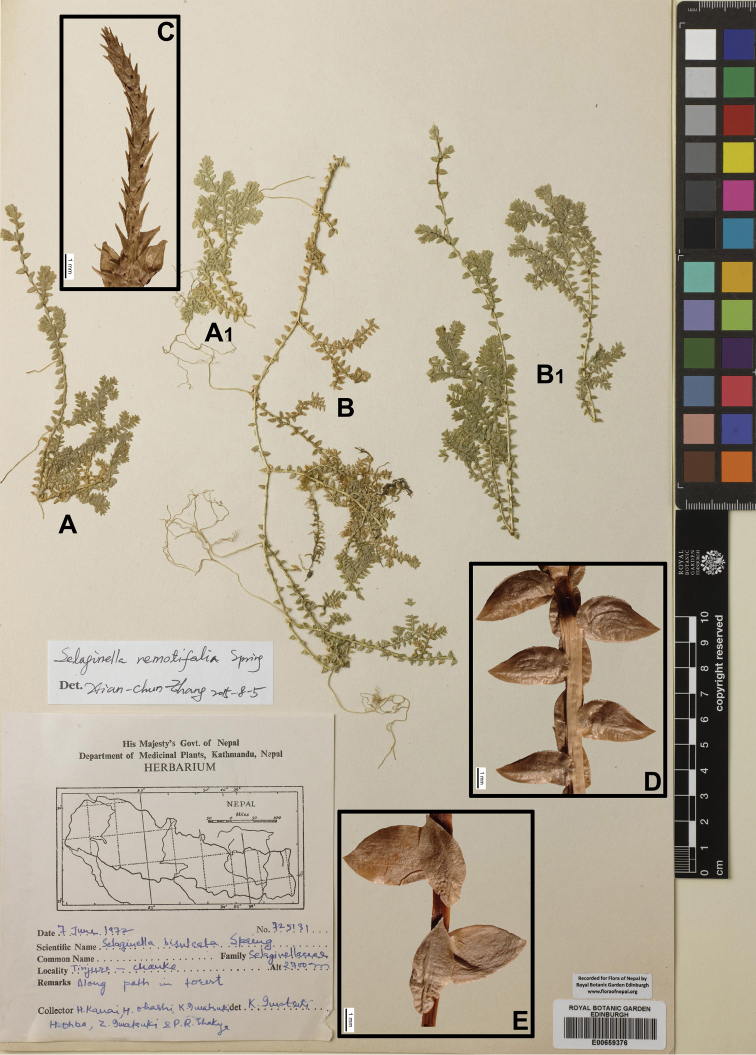
*Selaginella remotifolia* Spring. **A** (**A1**) Habit, upper surface of stem **B** (**B1**) habit, lower surface of stem **C** strobilus, upper surface **D** fragment of the lower surface of the lateral branches **E** Fragment of the upper surface of the lateral branches (**A, B, D, E***H. Kanai* et al. *725131*, E; **C***R.C. Ching 2192*, PE). Link: (http://data.rbge.org.uk/herb/E00659376).

**Figure 20.**
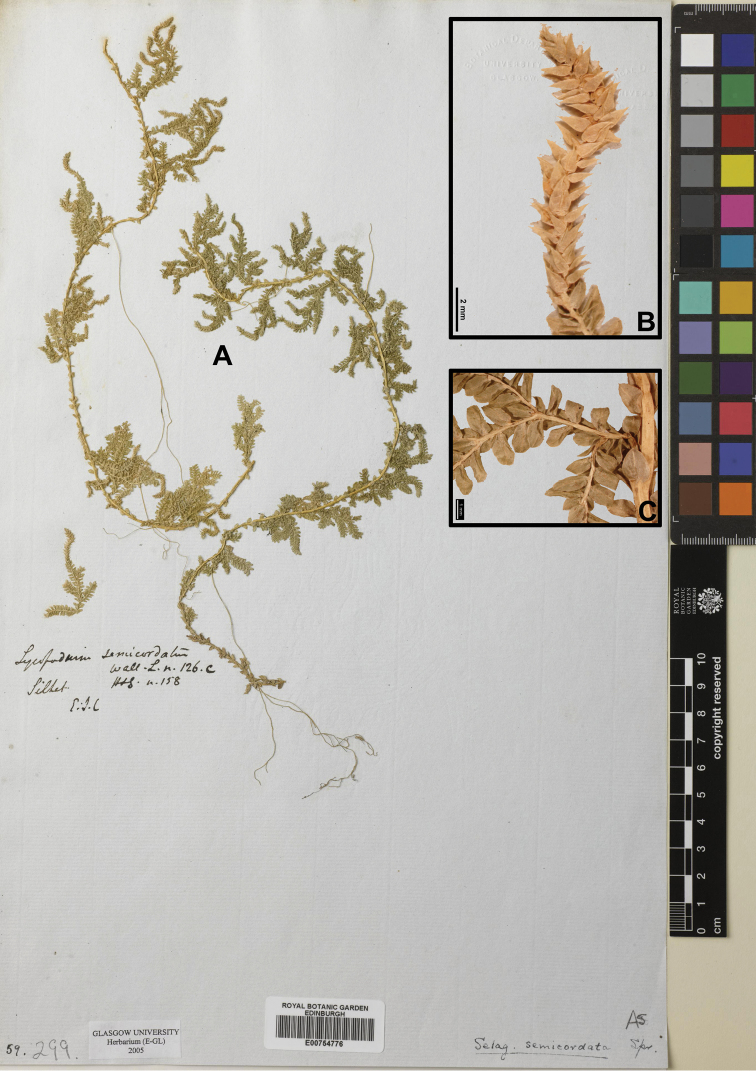
*Selaginella semicordata* (Wall. ex Hook. & Grev.) Spring. **A** Habit, lower surface **B** strobilus, lower surface **C** fragment of the lower surface of the lateral branches (*Wallich* n. *126.c*, E). Link: (http://data.rbge.org.uk/herb/E00754776).

**Figure 21.**
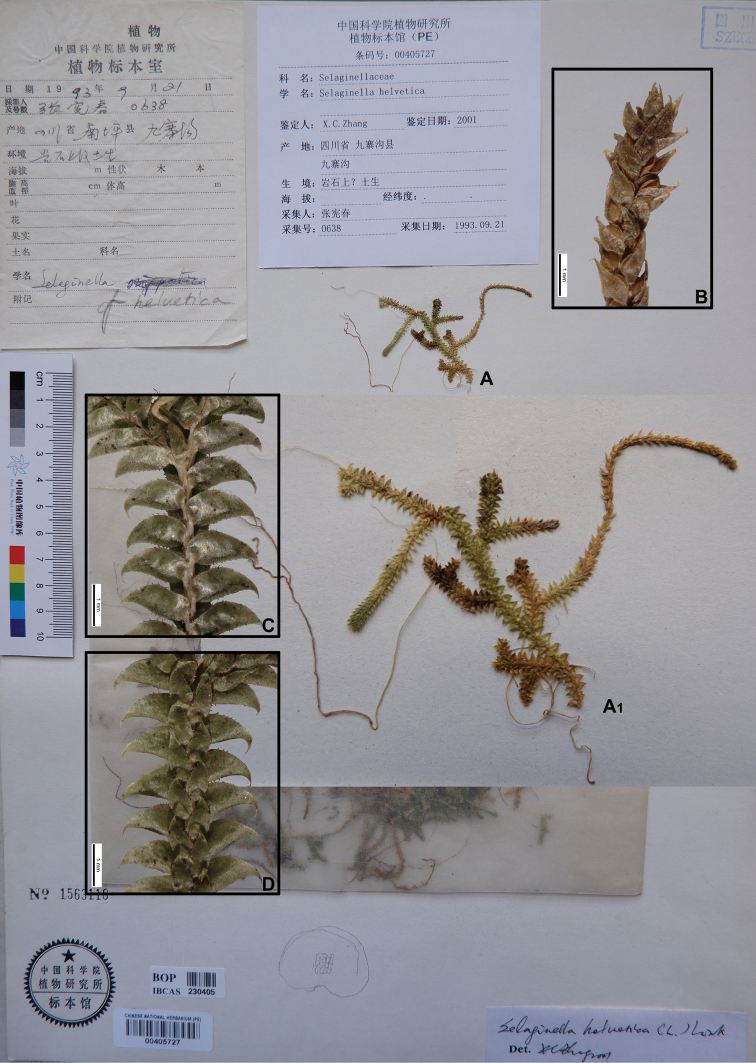
*Selaginella helvetica* (L.) Spring. **A** (**A1**) Habit, upper surface **B** fragment of the upper part strobilus **C** fragment of the lower surface of stem **D** fragment of the upper surface of stem (*X.C. Zhang 0638*, PE).

**Figure 22.**
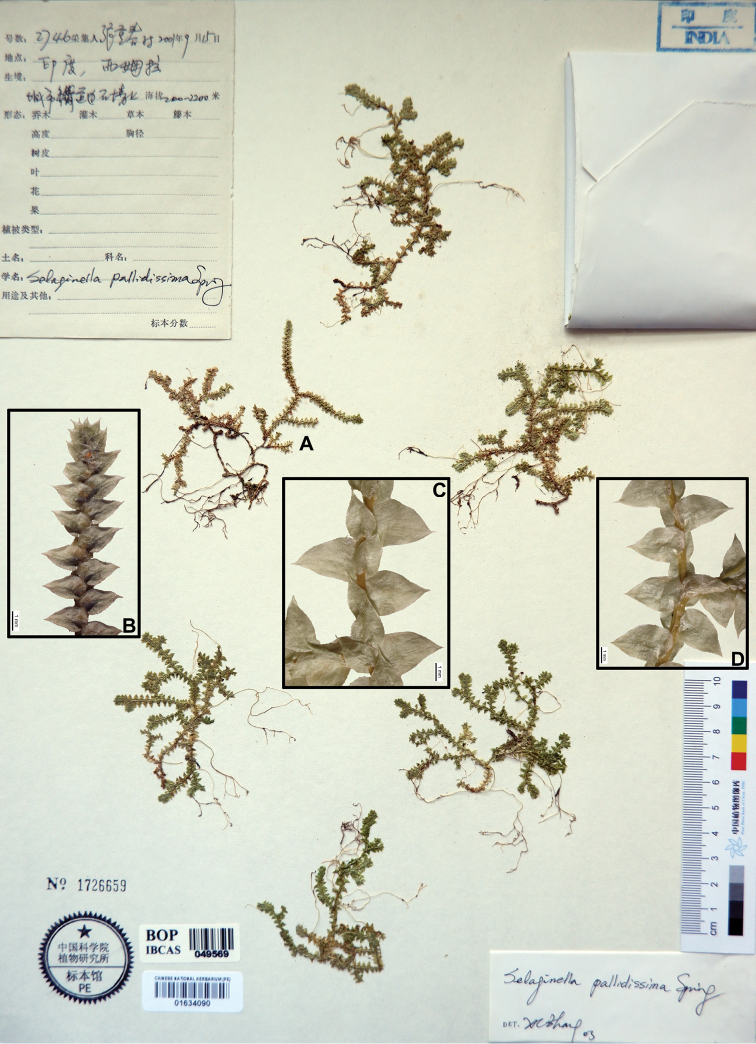
*Selaginella pallidissima* Spring, **A** Habit **B** strobilus, lower surface **C** fragment of the upper surface of the lateral branches **D** fragment of the lower surface of the lateral branches (*X.C. Zhang 2746*, PE).

**Figure 23.**
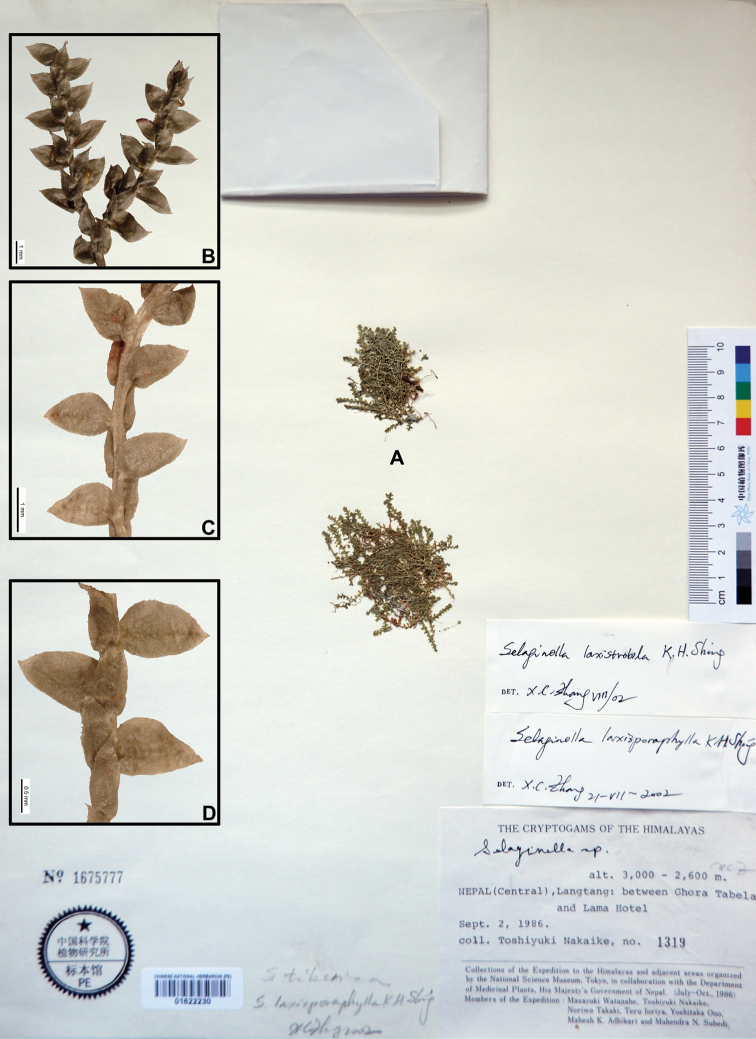
*Selaginella laxistrobila* K.H. Shing **A** Habit **B** Upper surface of strobilus **C** Lower surface of branches **D** Upper surface of branches (*T. Nakaike 1319*, PE).

**Figure 24.**
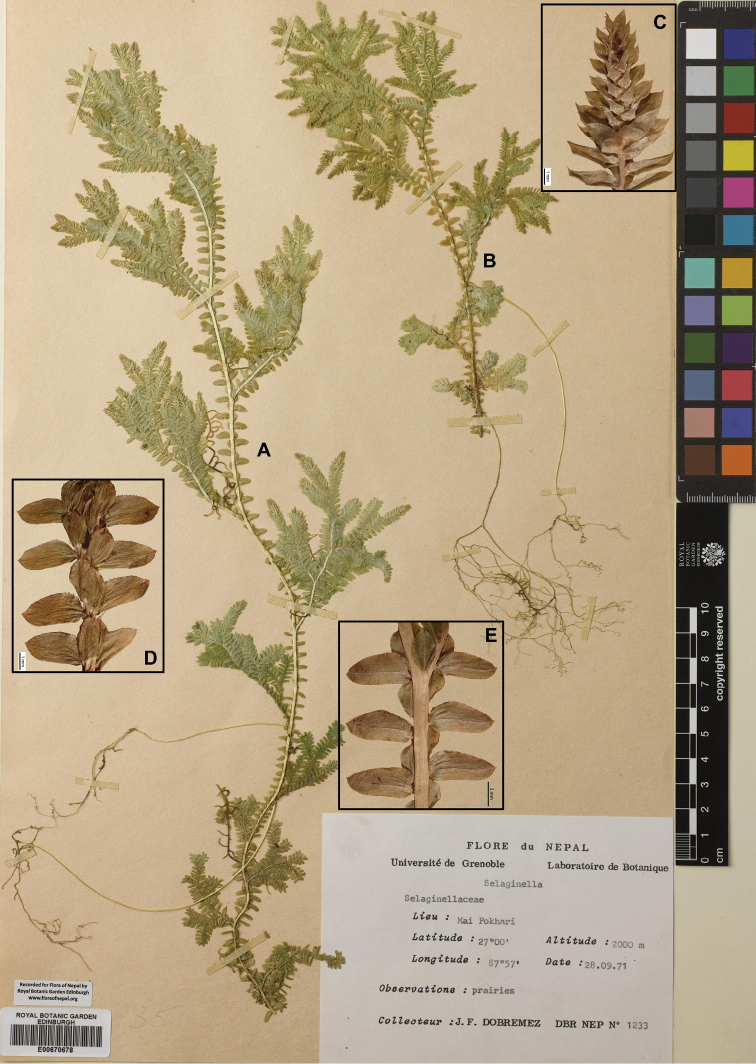
*Selaginella bisulcata* Spring. **A** Habit, lower surface **B** habit, upper surface **C** strobilus, lower surface **D** fragment of the upper surface of the lateral branches **E** fragment of the lower surface of the lateral branches (*J.F. Dobremez* DBR NEP *1233*, E). Link: (http://data.rbge.org.uk/herb/E00670678).

**Figure 25.**
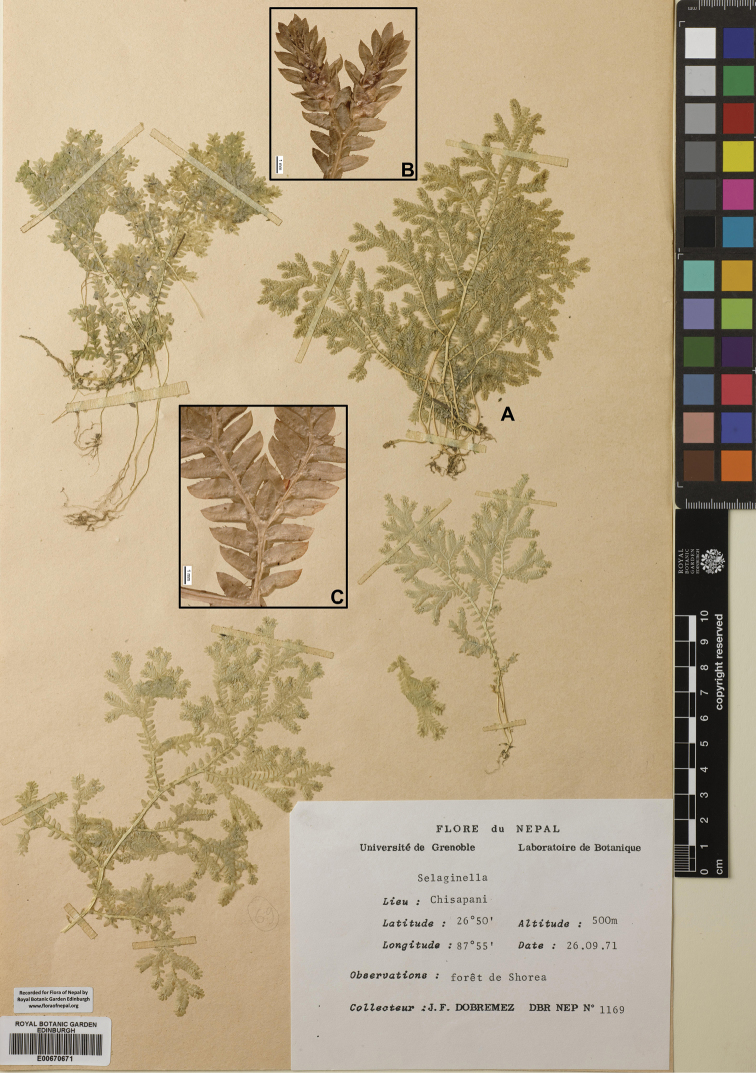
*Selaginella pennata* (D. Don) Spring. **A** Habit, lower surface **B** strobilus, lower surface **C** fragment of the lower surface of the lateral branches (*J.F. Dobremez* DBR NEP *1169*, E). Link: (http://data.rbge.org.uk/herb/E00670671).

**Figure 26.**
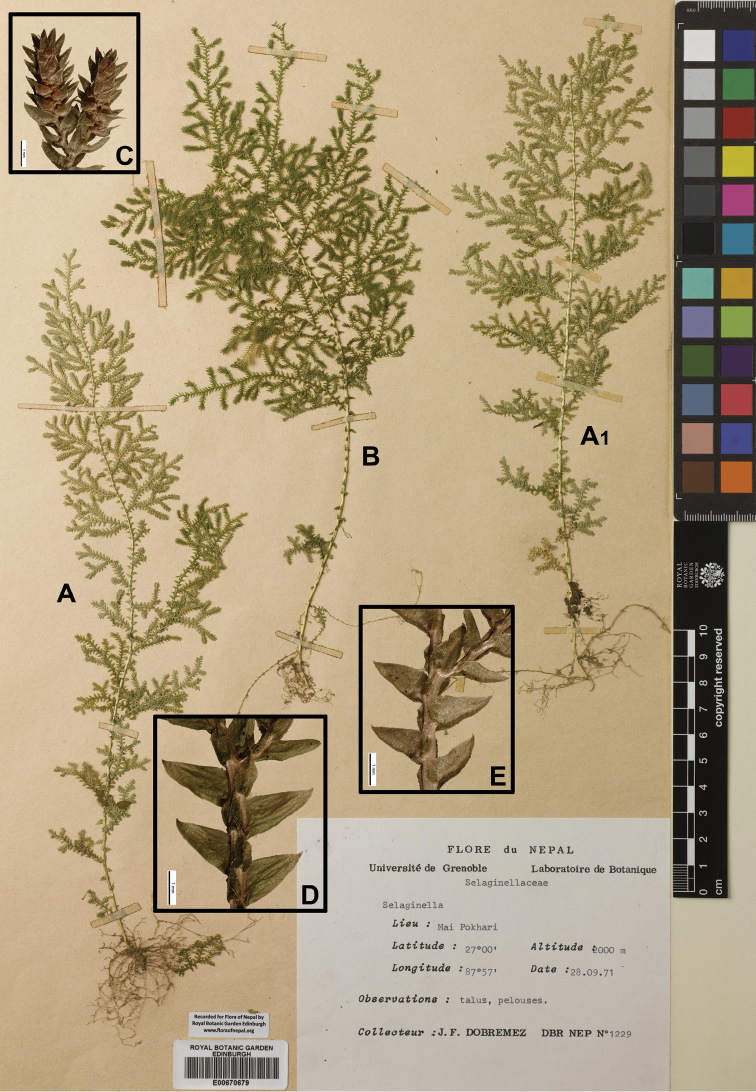
*Selaginella chrysocaulos* (Hook. & Grev.) Spring. **A** (**A1**) Habit, lower surface **B** habit, upper surface **C** strobilus, lower surface **D** fragment of the upper surface of the lateral branches **E** fragment of the lower surface of the lateral branches (*J. F. Dobremez* DBR NEP *1229*, E). Link: (http://data.rbge.org.uk/herb/E00670679).

**Figure 27.**
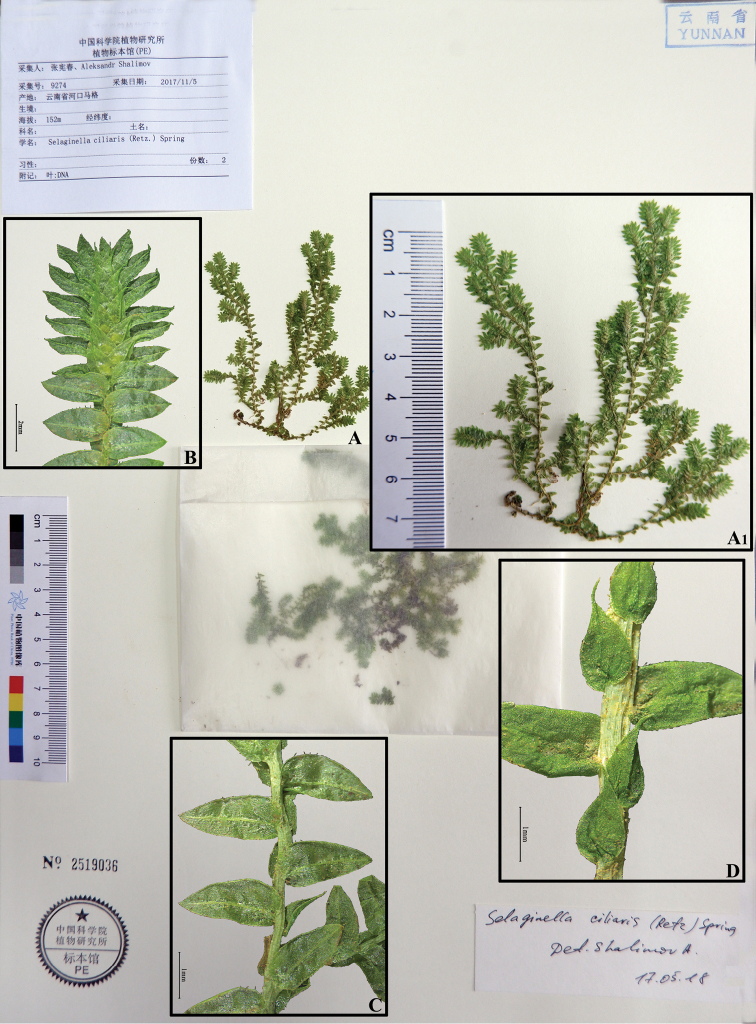
*Selaginella ciliaris* (Retz.) Spring **A** (**A1**) Habit, lower surface **B** Strobilus, lower surface **C** Fragment of the lower surface of the main stem **D** Fragment of the upper surface of the main stem (*X.C. Zhang* & *A. Shalimov 9274*, PE).

**Figure 28.**
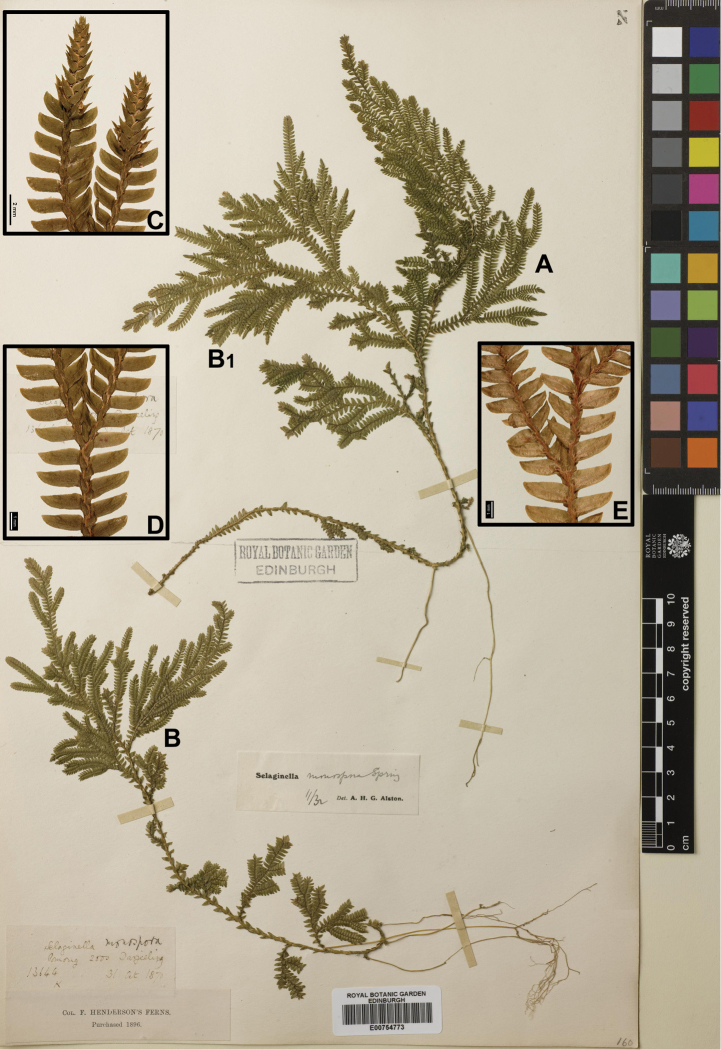
*Selaginella monospora* Spring **A** Habit, upper surface **B** (**B1**) habit, lower surface **C** strobilus, upper surface **D** fragment of the upper surface of the lateral branches **E** fragment of the lower surface of the lateral branches (*F. Henderson 13644*, E). Link: (http://data.rbge.org.uk/herb/E00754773).

**Figure 29.**
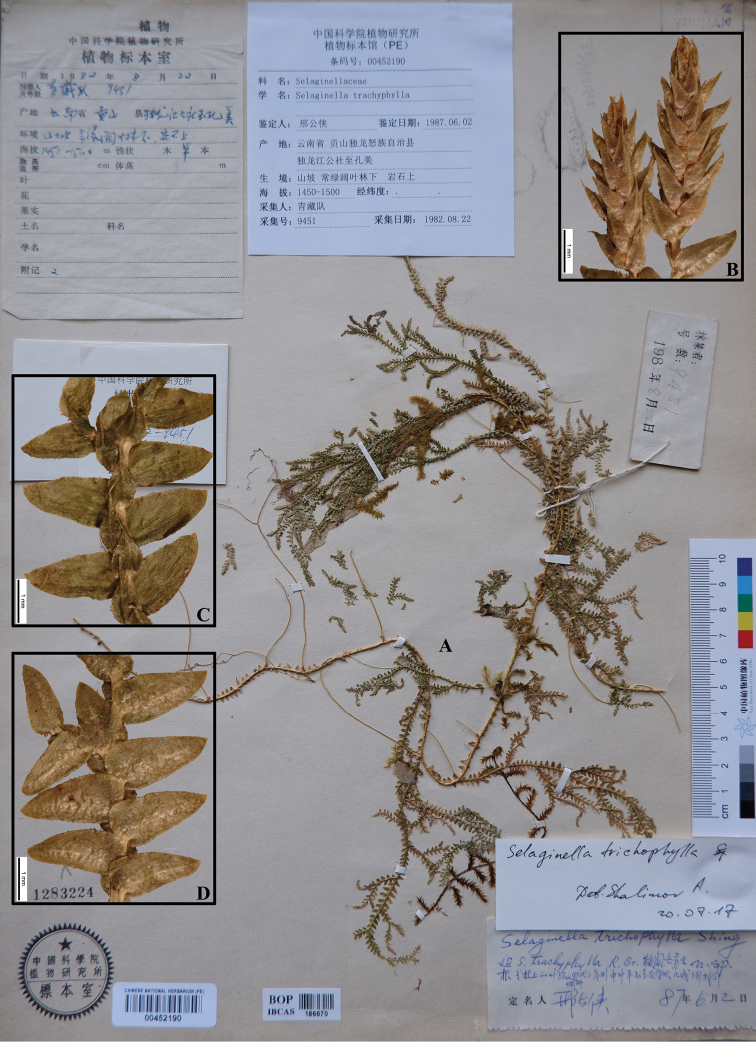
*Selaginella trichophylla* K. H. Shing **A** Habit **B** strobilus, lower surface **C** fragment of the upper surface of the lateral branches **D** fragment of the lower surface of the lateral branches (*Qinghai-Xizang Exped. 9451*, holotype: PE).

**Figure 30.**
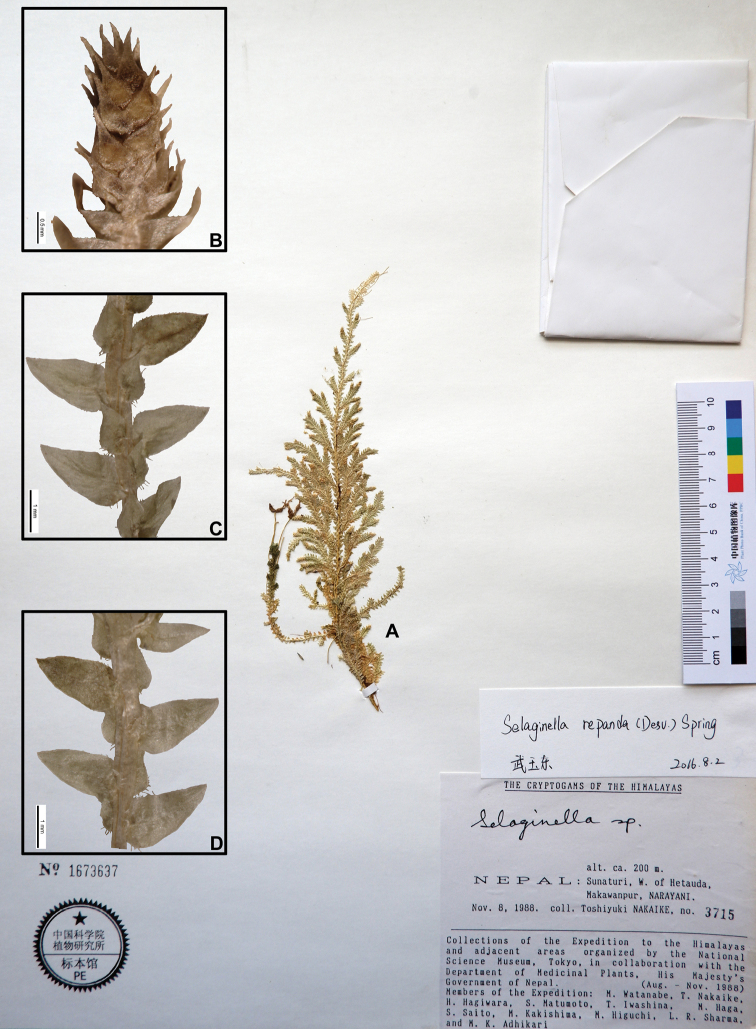
*Selaginella repanda* (Desv. ex Poir.) Spring **A** Habit, lower surface **B** strobilus, lower surface **C** fragment of the upper surface of the lateral branches **D** fragment of the lower surface of the lateral branches (*T. Nakaike 3715*, PE).

**Figure 31.**
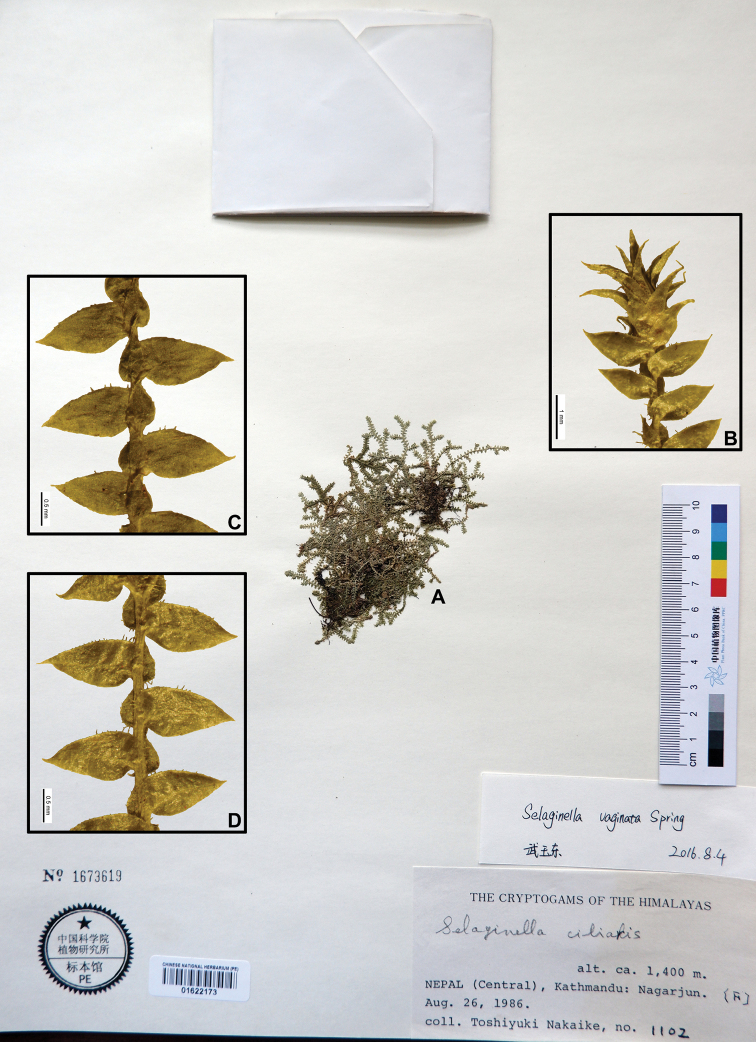
*Selaginella vaginata* Spring **A** Habit **B** strobilus, lower surface **C** fragment of the upper surface of the lateral branches **D** fragment of the lower surface of the lateral branches (*T. Nakaike 1102*, PE).

**Figure 32.**
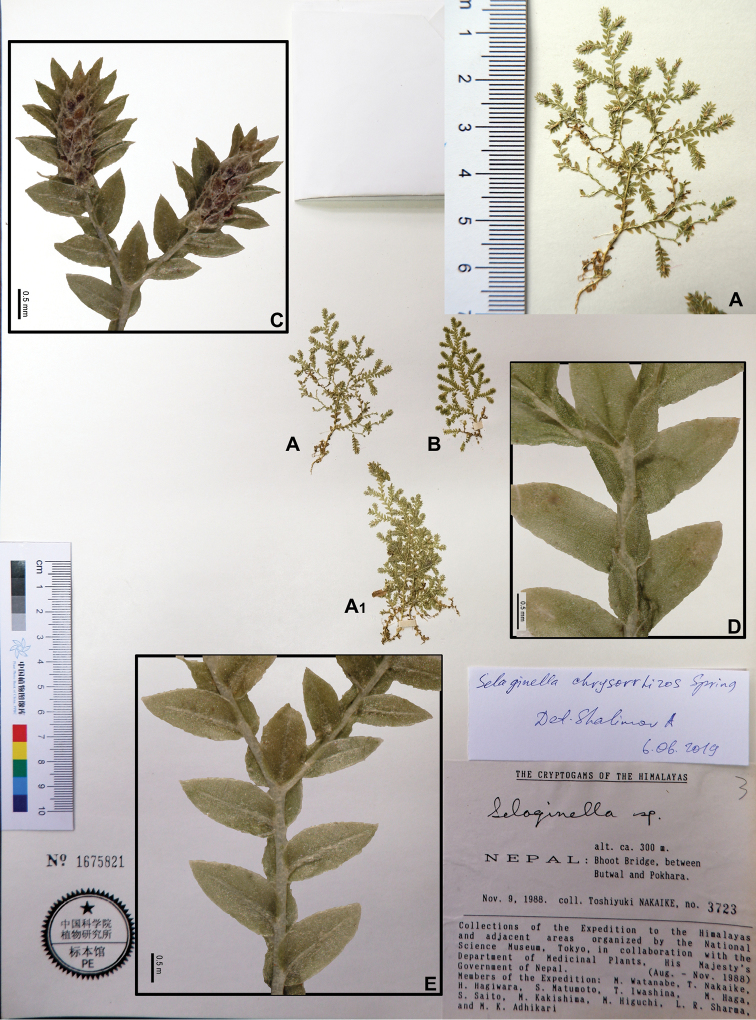
*Selaginella reticulata* (Hook. & Grev.) Spring **A** (**A1**) Habit, lower surface **B** strobilus, lower surface **C** fragment of the lower surface of the lateral branches **D** fragment of the upper surface of the lateral branches (*T. Nakaike 1760*, PE).

**Figure 33.**
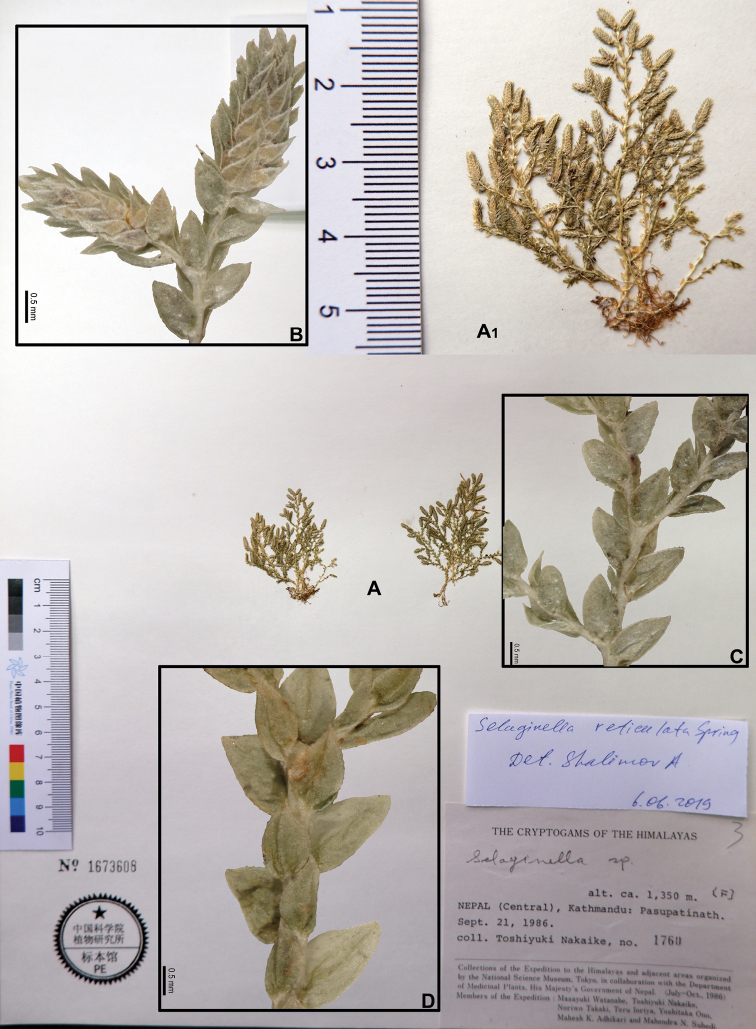
*Selaginella reticulata* (Hook. & Grev.) Spring **A** (**A1**) Habit, lower surface **B** strobilus, lower surface **C** fragment of the lower surface of the lateral branches **D** fragment of the upper surface of the lateral branches (*T. Nakaike 1760*, PE).

**Figure 34.**
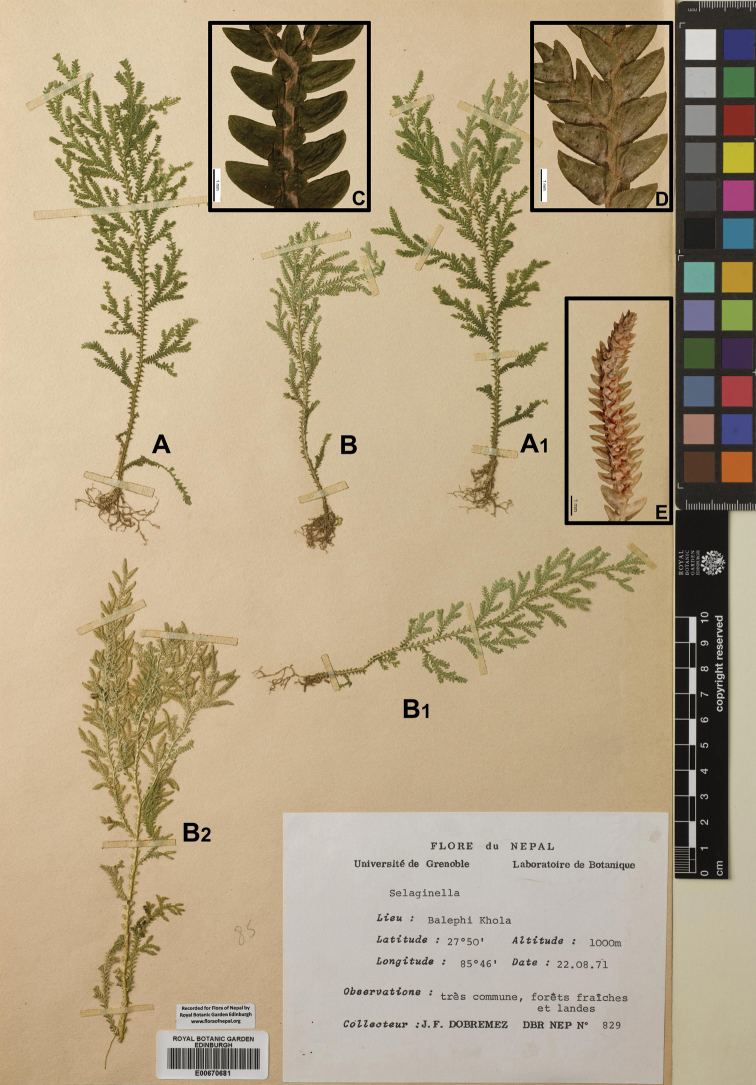
*Selaginella subdiaphana* (Wall. ex Hook. & Grev.) Spring **A** (**A1**) Habit, upper surface **B** (**B1, B2**) habit, lower surface **C** fragment of the upper surface of the lateral branches **D** fragment of the lower surface of the lateral branches **E** Strobilus, lower surface (*J.F. Dobremez DBR NEP 829*, E). Link: (http://data.rbge.org.uk/herb/E00670681).

**Figure 35.**
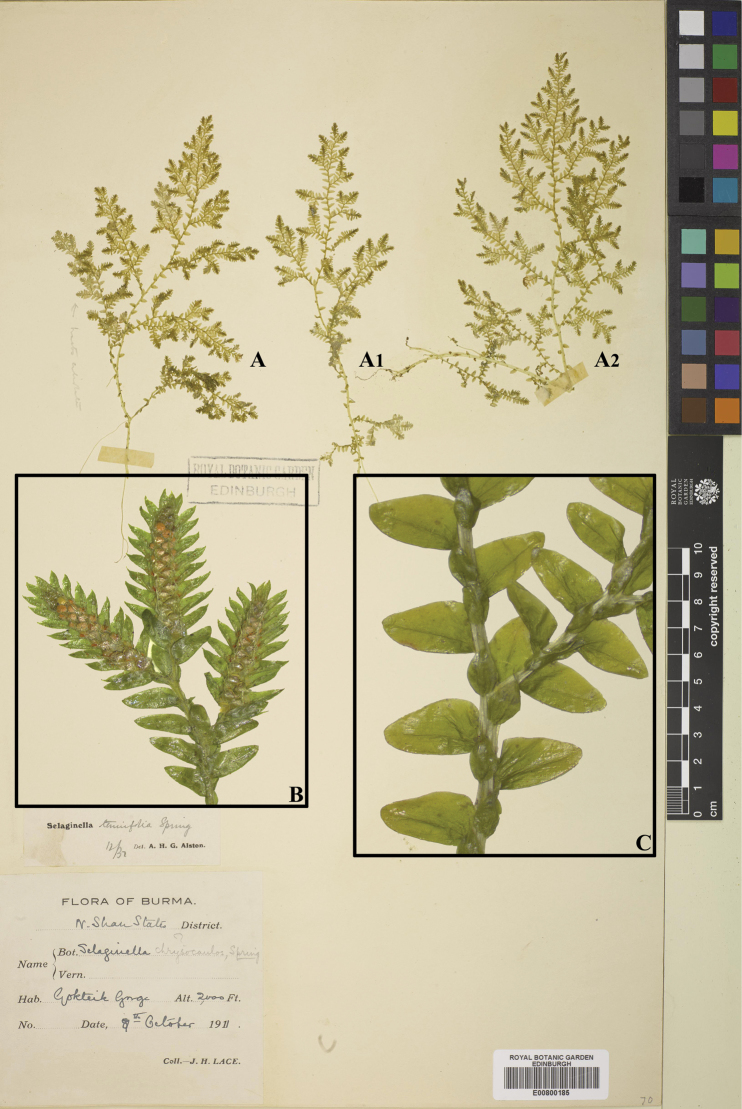
*Selaginella tenuifolia* Spring **A** (**A1, A2**) Habit, upper surface (J.H. Lance s.n., E) **B** Strobilus, lower surface **C** Fragment of the upper surface of the lateral branches (**A** (**A1, A2**) J.H. Lace s.n., E; **B, C** PE-Xizang Expedition PE 6280, PE) Link: (http://data.rbge.org.uk/herb/E00800185).

## Supplementary Material

XML Treatment for Selaginella indica

XML Treatment for Selaginella pulvinata

XML Treatment for Selaginella bryopteris

XML Treatment for Selaginella adunca

XML Treatment for Selaginella aitchisonii

XML Treatment for Selaginella fulcrata

XML Treatment for Selaginella involvens

XML Treatment for Selaginella pallida

XML Treatment for Selaginella remotifolia

XML Treatment for Selaginella semicordata

XML Treatment for Selaginella helvetica

XML Treatment for Selaginella pallidissima

XML Treatment for Selaginella laxistrobila

XML Treatment for Selaginella bisulcata

XML Treatment for Selaginella pennata

XML Treatment for Selaginella chrysocaulos

XML Treatment for Selaginella ciliaris

XML Treatment for Selaginella monospora

XML Treatment for Selaginella trichophylla

XML Treatment for Selaginella repanda

XML Treatment for Selaginella vaginata

XML Treatment for Selaginella chrysorrhizos

XML Treatment for Selaginella reticulata

XML Treatment for Selaginella subdiaphana

XML Treatment for Selaginella tenuifolia
